# H3K9me3 controls epidermis morphogenesis by regulating RNA Pol II dynamics at developmental promoters and enhancers

**DOI:** 10.1038/s41467-026-73308-5

**Published:** 2026-05-15

**Authors:** Chris Ke Bai, Gopal Chovatiya, Emily Janine Pollack, Yu-Ching Liao, Ashley Nayeon Kim, Tudorita Tumbar

**Affiliations:** 1 Department of Molecular Biology and Genetics, Cornell University, Ithaca, NY USA; 2 Present Address: Department of Pathology, Yale School of Medicine, New Haven, CT USA

**Keywords:** Cell lineage, Gene silencing, Skin stem cells, Transcriptional regulatory elements

## Abstract

Histone H3K9me3 silences repetitive elements and represses non-lineage genes during early development, but its role in organogenesis is understudied. Here, we show that H3K9me3 deposition is dynamic during epidermis morphogenesis and essential for lineage diversification. We ablate Suv39h1, Suv39h2, and Setdb1 histone methyltransferases, in the embryonic mouse epidermis, to induce H3K9me3 loss. This causes complete failure of keratinocyte differentiation, skin barrier formation, hair follicle development, and Merkel cell specification. Single-cell transcriptomics reveals aberrant cell fates with mixed epidermal subtype identities and dysregulated non-lineage and lineage-specific transcription programs. Affected pathways include differentiation, metabolism, cell cycle, cytoskeletal organization, and extracellular matrix. H3K9me3 primarily restricts RNA Pol II transcription initiation at key developmental promoters and enhancers and has minimal direct effect on promoter-proximal pause release. We uncover a cooperative and indispensable role for Suv39h1, Suv39h2, and Setdb1 in gene expression control of epidermal morphogenesis, establishing H3K9me3 as a critical developmental determinant of skin organogenesis.

## Abstract (web summary)

This study reveals that H3K9me3 dynamically regulates lineage-related genes during epidermis development by repressing RNA polymerase II activity at key enhancers and promoters. Loss of H3K9me3 results in mixed cell identity and failure of skin morphogenesis.

## Introduction

Histone modifications are important and widely studied epigenetic regulators during development, aging, reprogramming, and cancer^1,2^. Among these, tri-methylation of histone H3 at lysine 9 (H3K9me3) has been traditionally associated with constitutive heterochromatin and silencing of transposable elements^3,4^. However, in the past several years, increasing evidence suggests an additional role of H3K9me3 in repressing non-lineage genes^5,6^.

In mice, H3K9me3 global patterns are dynamically and progressively established de novo in a lineage-specific manner during embryogenesis^7^. Three histone methyltransferases (HMTs) are primarily responsible for the formation and spreading of H3K9me3 heterochromatic domains in mice, namely Suv39h1, Suv39h2, and Setdb1^8^. Suv39h1 and Suv39h2 are largely redundant and were initially thought to maintain constitutive heterochromatin and genomic stability and have little function in gene regulation^9^. Mice with double deletion of the two HMTs develop normally to mid-gestation, and can survive to adulthood, when they display increased DNA damage and susceptibility to cancer^9^. However, more recent studies suggested potential context-dependent roles of Suv39h enzymes in gene regulation in several tissues^10–12^.

On the other hand, Setdb1 was originally thought to primarily repress genes and transposable elements within euchromatin; its ubiquitous deletion led to developmental arrest of mouse embryos at the blastocyst stage^13^. Furthermore, cell-type-specific and/or inducible *Setdb1* single knockout mouse models revealed its role in adult stem cells in tissues such as the hair follicle, muscle, and blood^14–18^. In these reports, Setdb1 loss disrupted tissue homeostasis and regeneration, primarily through aberrant activation of transposable elements and the resulting inflammation, and in some cases by de-repressing non-lineage genes^14–18^. However, Setdb1 was also shown to affect pericentromeric H3K9me3 in spermatocytes, thus regulating meiosis through mechanisms related to constitutive heterochromatin maintenance^14^.

These studies are challenging the notion that Setdb1 and Suv39h enzymes occupy distinct spatial and functional roles, instead suggesting more nuanced and context-dependent regulation of H3K9me3 functions. Examining the role of H3K9me3 in organogenesis and accounting for any functional redundancies require simultaneous targeting of all three HMTs (e.g., *Suv39h1*, *Suv39h2,* and *Setdb1*) in a cell type specific manner.

Triple knockout (TKO) of all three H3K9me3 HMTs in specific cell types in vivo has only been performed in a single study to date, targeting the endoderm^19^. This revealed an important role of the three HMTs in repressing non-lineage genes at the onset of liver and pancreas specification, prior to organogenesis. Triple knockout of the H3K9me3 HMTs during mouse organogenesis, when tissues undergo extensive lineage diversification and morphogenesis, has not yet been performed.

In addition, the transcription silencing mechanisms associated with this essential histone mark are incompletely understood. Notably, previous genetic studies targeting individual HMTs in mice primarily measured changes in steady-state RNA levels, which reflect both transcriptional and post-transcriptional effects and can confound interpretation. Moreover, it remains unclear which specific steps of RNA Pol II regulation are influenced by H3K9me3 and whether it affects Pol II promoter-recruitment and transcription initiation or promoter-proximal pause-release into productive elongation^20^.

We address these fundamental gaps in the field using mouse skin epidermal development as a model system for mammalian organogenesis. Epidermis is composed of the epithelial or keratinocyte lineage, which arises from a single layer of ectoderm that covers the body at mouse embryonic day 9.5 (E9.5). Through sequential steps of cellular diversification and morphogenetic rearrangements that culminate at E18.5 prior to mouse birth, the keratinocyte lineage forms the stratified epidermis and its appendage, the hair follicle^21,22^. Epidermis also harbors specialized innervated structures, the touch domes, that contain sensory epithelial Merkel cells^23^. As skin matures into adulthood, the hair follicle relies on hair follicle stem cells to orchestrate cycles of hair growth, regression, and rest during tissue homeostasis^24,25^.

In this work, we delete all three HMTs during skin development with an inducible epithelial-specific TKO mouse model, which results in depletion of H3K9me3 and profound defects in epidermis morphogenesis. Using PReCIS-seq, we map keratinocyte-specific, transcriptionally engaged RNA Pol II in intact tissue^26^. With complementary genomic approaches, we reveal dynamic H3K9me3 narrow islands that repress RNA Pol II activity at specific enhancers and promoters to regulate cell adhesion, differentiation, proliferation, and metabolism during epidermal development. In contrast, DNA damage, apoptosis, and inflammation are largely unaffected at this embryonic stage, contrary to expectation from previous work. We demonstrate that H3K9me3 is indispensable for lineage progression and diversification by limiting Pol II activity primarily at recruitment/initiation step with minimal direct effects on pause-release. We uncover here an essential role for H3K9me3 as a key regulator during mammalian organogenesis.

## Results

### H3K9me3 dynamically marks lineage promoters and enhancers

**Fig. 1:**
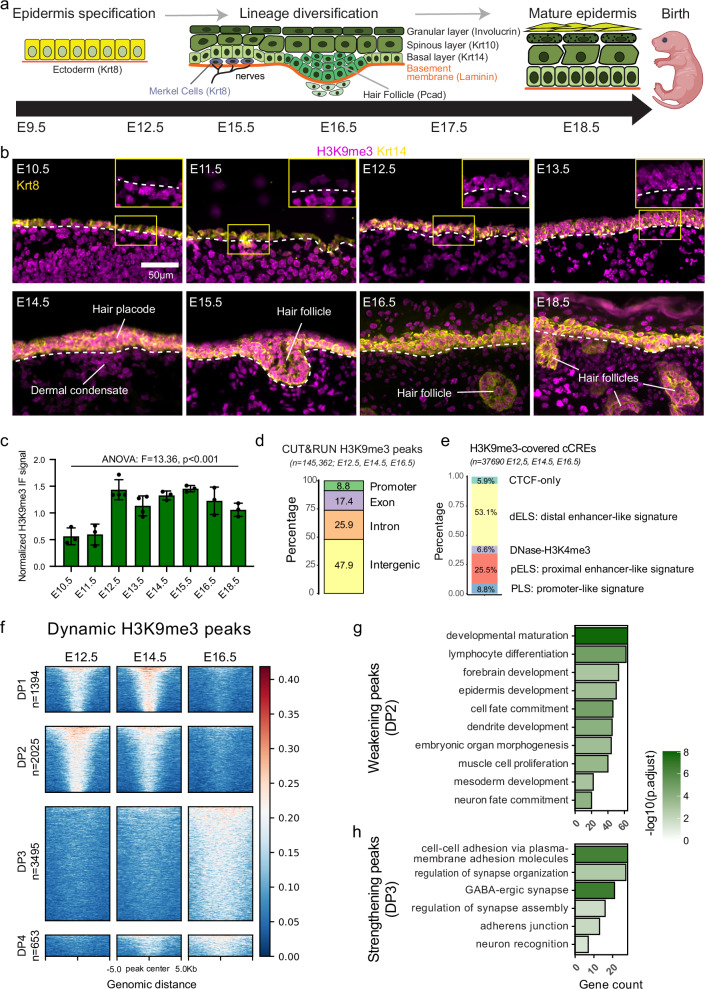
Dynamic H3K9me3 landscapes mark promoters and enhancers of lineage-related genes during epidermis development. **a** Schematics and timeline of epidermal morphogenesis during skin development. **b** Embryonic (E) skin at days indicated was immunofluorescence (IF) stained for H3K9me3 (magenta), Krt8 (yellow, ectoderm), or Krt14 (yellow, basal layer). Images are representative. **c** Quantifications for images like those in (**b**), showing H3K9me3 hypomethylation of the basal layer prior to lineage diversification. *n* = 4 embryos for E12.5, E13.5, and *n* = 3 embryos for all other time points. Mean H3K9me3 signal intensity of Krt8/Krt14 positive basal cells was quantified and normalized to mean H3K9me3 intensity from adjacent dermal cells. One-way ANOVA with pairwise comparisons (Tukey’s method) was performed for all time points. Data are presented as mean ± s.d. See Supplementary Fig. 1a for exact *p* values for all pairwise comparisons. **d** Pie chart showing the distribution of functional genomic features overlapping called H3K9me3 peaks. If a peak overlaps multiple genomic features due to alternative TSS or alternative splicing, feature types are assigned following this order: promoter, exon, intron and intergenic. **e** Types of ENCODE-defined candidate cis-regulatory elements (cCREs) covered by H3K9me3. Classification criteria for cCREs can be found from UCSC website. **f** Heatmap showing genome-wide dynamic H3K9me3 peaks across epidermis development. Selected GO terms enriched in genes associated with dynamic peaks, **g** for weakening peaks (DP2) and **h** for strengthening peaks (DP3). Gene-peak association is defined if H3K9me3 peak(s) overlaps gene body or ±10 kb flanking region of a gene. GO analysis was performed using clusterProfiler based on a one-sided hypergeometric test with Benjamini–Hochberg correction for multiple comparisons. Full lists of enriched GO terms are reported in Supplementary Data 2. Created in BioRender. Bai, C. (2026) https://BioRender.com/m3uab5j.

We first assessed H3K9me3 levels and genome-wide landscapes during keratinocyte-lineage diversification. The epidermis arises from an ectodermal K8^+^ (Keratin 8, Krt8) layer at embryonic day 9.5 (E9.5), which subsequently matures into K14^+^ (keratin 14, Krt14) basal progenitors that diversify to multiple keratinocyte subtypes^27^, including suprabasal cells, hair follicles, and Merkel cells (Fig. 1a). Immunofluorescence (IF) staining revealed that global H3K9me3 levels start low in the ectodermal K8^+^ layer but significantly increase around E12.5 and maintain relatively stable thereafter during skin morphogenesis (Fig. 1b, c, and Supplementary Fig. 1a).

We then isolated epidermal cells from E12.5, E14.5, and E16.5 mouse embryos using fluorescence-activated cell sorting (FACS) (Supplementary Fig. 1b, c) and profiled the genome-wide H3K9me3 distribution by CUT&RUN. Using Epic2, a peak caller optimized for broad domains^28^, we identified around 37,000–54,000 H3K9me3 peaks from each developmental timepoint, with their sizes ranging from 500 bp to 1.8 Mb (Supplementary Fig. 1d). Around half of these peaks (52.1%) overlap genic regions including promoters (8.8%), exons (17.6%) and introns (25.9%), while the other half reside in intergenic regions (Fig. 1d). A subset of peaks overlaps ENCODE-defined candidate cis-regulatory elements (cCREs), with enrichment in proximal and distal enhancer-like signatures (78.6%) (Fig. 1e). This suggests that H3K9me3 islands mark select promoters and enhancers in epidermis.

Using the union of peaks from all timepoints as reference (Supplementary Fig. 1e), we counted H3K9me3 signals and defined dynamic peaks (DP1–4) across development (Fig. 1f). Among them, DP1 and DP2 are weakened from E12.5 to E16.5, while DP3 and DP4 are strengthened. To assess whether dynamic peaks regulate developmental genes, we define that a gene is H3K9me3-associated if a peak overlaps its gene body or ±10 kb flanking regions. We then performed GO analysis and found both strengthening and weakening peaks are associated with genes enriched for development-related terms, such as “epidermis development,” “embryonic organ morphogenesis,” and so on (Fig. 1g, h, and Supplementary Fig. 1f, g). Interestingly, constant peaks, which did not significantly change, are also associated with genes enriched for development-related terms, indicating repression of genes from non-epidermal lineages (Supplementary Fig. 1h).

Together, these data show that H3K9me3 increases in the ectoderm prior to skin morphogenesis and then dynamically marks select promoters and putative enhancers during epidermal lineage diversification. A subset of peaks associates with developmental genes and likely regulates their expression by repressing promoters and enhancers during skin organogenesis.

### H3K9me3 loss impairs epidermal lineage diversification

**Fig. 2:**
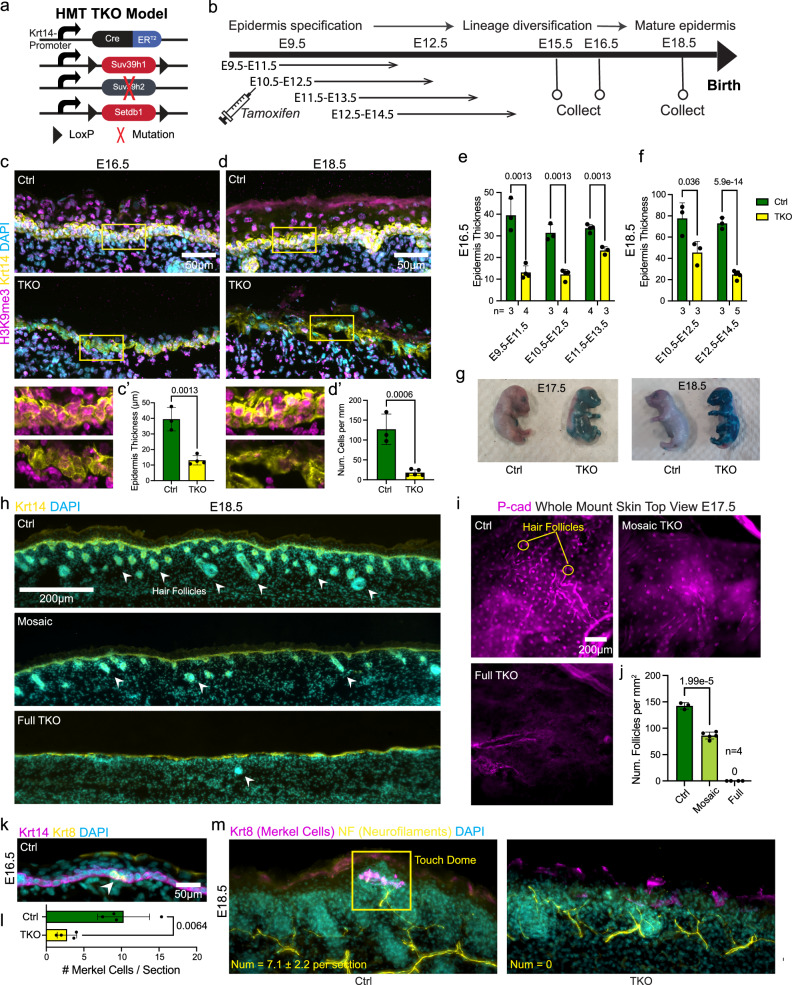
Loss of H3K9me3 disrupts epidermal lineage diversification and skin morphogenesis. **a** Schematic showing the construct of the keratinocyte-specific triple knockout (TKO) line. **b** Timeline of TM injection and sample collection. IF staining of H3K9me3 (magenta) and Krt14 (yellow) showing reduced stratification at E16.5 (**c**) and reduced cellularity at E18.5 (**d**). Insets highlight loss of H3K9me3. **c’** Average thickness quantified by dividing the total area of epidermis by basement membrane (BM) length. TM E10.5–12.5; *n* = 3 Ctrl and *n* = 4 TKO embryos. **d’** Basal cell density quantified as Krt14+ cells per millimeter TM E12.5–14.5; *n* = 3 Ctrl and *n* = 5 TKO embryos. Quantifications of epidermis thickness at E16.5 (**e**) and E18.5 (**f**), showing different tamoxifen induction timelines. The exact *n* values are shown on plots. *P* values were corrected for multiple comparison using the Holm–Sidak method. **g** X-gal barrier assay results showing impaired skin barrier function. TM E10.5–12.5; *n* = 2 embryos per group. **h** IF staining showing defective hair follicle development. Arrow heads exemplify hair follicles. TM E10.5–12.5. Images are representative from *n* = 3 embryos per group. **i** Whole-mount staining for P-cad (magenta) showing hair follicle as dots (see yellow circles). Note the complete absence of hair follicles in full TKO. TM E10.5–12.5; **j** Quantification of hair follicle numbers. Ctrl, *n* = 3 embryos; mosaic, *n* = 5 embryos; full, *n* = 4 embryos. **k**, **l** IF staining and quantification for Merkel cells (Krt8+/Krt14+, arrow heads) in Ctrl at E16.5. TM from E9.5–11.5; *n* = 4 embryos per group. Three sections per embryo were quantified. **m** IF staining for Merkel cells (Krt8+) and sensory nerves (NF+) showing touch domes in E18.5 embryos. Note that touch domes fail to form in TKO, and random regions were imaged for comparison. Quantification of dorsal touch dome number is shown on the images. TM from E12.5–14.5; *n* = 4 embryos per group; 2 sections per embryo. All images are representative. Statistical significance was assessed using two-sided unpaired t-tests. Data are presented as mean ± s.d.

We next tested whether H3K9me3 is functionally required for epidermal lineage diversification during development (Fig. 1a). To investigate this, we generated a tamoxifen-inducible epithelial-specific triple knockout (TKO) model by crossing *Suv39h1*^*flox/flox*^*; Suv39h2*^*KO/KO*^*; Setdb1*^*flox/flox*^ mice^19^ with the *K14-CreER*^*T2*^ driver line^29^ (Fig. 2a, b).

As constitutive *Suv39h2* deletion shows no overt phenotypes as reported^19^, we use the Cre-negative littermates as controls (Ctrl) unless otherwise noted. The Cre-mediated ablation of *Suv39h1* and *Setdb1* was confirmed by IF staining for their proteins (Supplementary Fig. 2a–d). Interestingly, Ctrl epidermis expressed Suv39h1 in basal cells only, whereas Setdb1 was broadly expressed, suggesting their nonredundant roles.

HMT loss induced at all examined time points eventually caused H3K9me3 depletion (Fig. 2c, d, and Supplementary Fig. 2e–j). Notably, H3K9me3 loss is often mosaic due to incomplete Cre targeting and takes several days to full depletion, displaying ‘all-or-none’ patterns in the epidermis at E16.5 or E18.5 (but not E15.5) (Supplementary Fig. 2e–j). A summary of embryos analyzed across time points and induction schemes is provided in Supplementary Data 1.

With successful knockout at E16.5 or E18.5, TKO embryos exhibited a marked reduction in epidermal thickness and impaired stratification, in all the induction schemes tested (Fig. 2c–f). By E18.5, the epidermis also displayed a substantial reduction in basal cell number and loss of the skin barrier function (Fig. 2d’, g).

Beyond stratification defects, TKO embryos showed a profound impairment in hair follicle morphogenesis in both embryo skin sections stained for Krt14 (Fig. 2h) and in whole-mount skin with hair follicles marked by P-cadherin (Fig. 2i, j). In addition, Merkel cells were significantly reduced by E16.5 and the innervated touch domes that they make failed to form by E18.5 in TKO skin (Fig. 2k–m).

Together, these findings demonstrate that H3K9me3 is essential for keratinocyte lineage diversification and skin barrier formation, with its loss severely disrupting epidermis morphogenesis.

### H3K9me3 regulates cell proliferation and differentiation

To assess the functional consequences of H3K9me3 loss during epidermal development on specific cellular functions, we examined DNA integrity, cell survival, proliferation, and differentiation in embryonic skin of TKO and Ctrl mice.

**Fig. 3:**
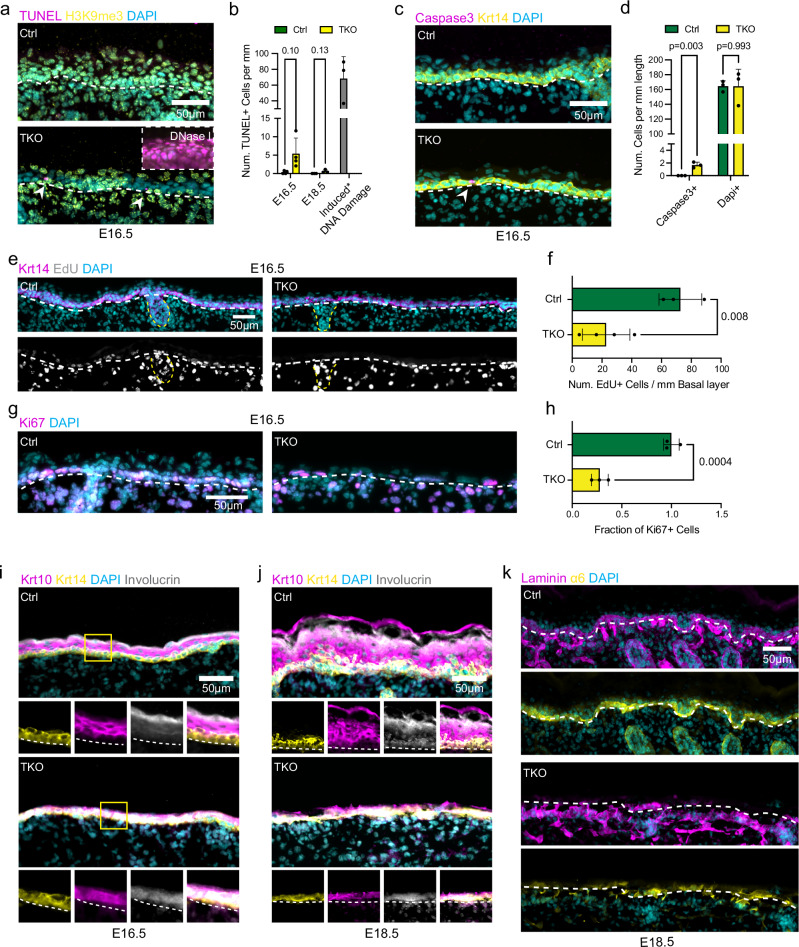
H3K9me3 loss impairs cellular functions essential for tissue development without inducing significant genomic instability. **a** Images of terminal deoxynucleotidyl transferase dUTP nick-end labeling (TUNEL) assay showing marginal increase of DNA damage in TKO epidermis. White dashed line marks basal membrane. TUNEL+ cells are indicated with arrow heads. Inset shows an image from positive control created by treating skin sections with DNase I. **b** Quantification of TUNEL+ cell density (per mm) for images like those in (**a**). See also Supplementary Fig. 4e for images of E18.5 samples and UVB irradiated skin labeled as Induced DNA damage (*), which was used as positive staining control. E16.5, TM from E9.5–11.5, *n* = 4 embryos per group; E18.5, TM from E12.5–14.5, *n* = 3 embryos per group; *n* = 3 mice for the induced control. **c**, **d** IF staining for active caspase3 (magenta) and quantification (**d**) of caspase3+ cell density (per mm), showing sparse apoptotic cells in E16.5 TKO epidermis. *N* = 3 embryos per group, TM E9.5–11.5. **e**, **f** IF staining for EdU (gray) and quantification (**f**) showing the proliferation arrest of TKO basal cells. TM E9.5–11.5; *n* = 3 Ctrl and *n* = 4 TKO embryos. **g**, **h** IF staining for Ki67 (magenta) and quantification (**h**) showing reduced proliferation in TKO basal cells. TM from E9.5–11.5; *n* = 3 embryos per group. **i**,** j** IF staining showing defective epidermis stratification in TKO epidermis. Krt14 (yellow) marks basal layer; Krt10 (magenta) marks spinous and granular layers; involucrin (gray) marks granular layer. Cornified envelope is also visible as auto-fluorescence in the red channel. Insets show enlarged, single-channel images highlighting the structural positions of different layers. E16.5, TM E10.5–12.5; E18.5, TM E12.5–14.5. *N* = 3 embryos per group. **k** IF staining for laminin-α1 and integrin-α6 in E18.5 embryos showing basement membrane defects in TKO samples. TM from E12.5–14.5. *N* = 3 embryos per group. White dashed lines mark basement membrane; yellow dashed lines mark hair follicles. All images are representative. Statistical significance was assessed using two-sided unpaired t-tests. Data are presented as mean ± s.d.

Previously, long-term H3K9me3 reduction implicated this mark in proper heterochromatin organization and genome integrity maintenance^4,9,30^. We therefore first asked whether its depletion perturbs chromatin organization and triggers DNA damage or apoptosis. IF for heterochromatin protein 1α (Hp1a), a known H3K9me3 interactor, revealed loss of nuclear Hp1a foci in many TKO basal cells, suggesting impaired heterochromatin function (Supplementary Fig. 3a, b). Additionally, TKO nuclei often became flatter and more elongated (Supplementary Fig. 3c, d), reminiscent of heterochromatin function in nuclear shape regulation^31^. However, TUNEL assays at E16.5 and E18.5 showed minimal DNA damage (Fig. 3a, b, and Supplementary Fig. 3e), and cleaved Caspase-3 staining indicated low apoptosis rates in TKO epidermis (Fig. 3c, d, and Supplementary Fig. 3f), suggesting cell survival is largely unaffected by H3K9me3 loss.

We next tested whether cell proliferation is reduced in TKO epidermis. EdU incorporation assays show fewer S-phase basal cells in TKO skin at both E16.5 (Fig. 3e, f) and E18.5 (Supplementary Fig. 3g, h), which is corroborated by reduced Ki67 staining (Fig. 3g, h). Thus, H3K9me3 loss impairs epithelial progenitor proliferation during epidermal morphogenesis.

We then asked whether epidermal differentiation was also affected. Co-staining for K14 (basal layer), K10 (spinous layer), and Involucrin (granular layer) at E16.5 showed well-ordered stratification in Ctrl but not TKO epidermis, despite the latter expressing all three markers (Fig. 3i). Notably, K14^+^ cells in TKO epidermis aberrantly co-express K10 and Involucrin, suggesting mis-regulated differentiation (Fig. 3i). By E18.5, basal cells remain disorganized and display irregular morphology (Fig. 3j), suggesting potential cytoskeletal defects.

We also examined basement membrane integrity, which is a well-documented critical factor for epidermal tissue polarity, differentiation, and signaling^32^. Although laminin-α1 expression appeared normal at E16.5 (Supplementary Fig. 3i), both laminin-α1 and integrin-α6 were disrupted across large areas of E18.5 TKO skin (Fig. 3k), indicating that H3K9me3-depleted cells fail to maintain the basement membrane architecture.

Together, these findings show that H3K9me3 loss in developing epidermis does not elicit notable genomic instability or apoptosis. Instead, we observe defects in cell proliferation, differentiation, cytoskeletal organization, and basement membrane maintenance, which together underlie the failure in keratinocyte lineage diversification.

### Reduced H3K9me3 mildly affects hair follicle regeneration

We also briefly examined H3K9me3’s role in adult epidermal homeostasis and hair regeneration by inducing the TKO at postnatal day 36. After hair plucking, no obvious epidermis or hair regeneration defects were observed in the H3K9me3 TKO mice (Supplementary Fig. 4a–c). Only 5–10% of regenerating follicles showed full H3K9me3 depletion, likely due to inefficiency of knockout at this stage. These follicles were smaller, but unlike TKO embryonic progenitors, which arrested proliferation and differentiation (Fig. 3), adult hair follicle progenitors remained proliferative and differentiated normally despite loss of H3K9me3 (Supplementary Fig. 4d–f). Consistent with embryonic findings, H3K9me3 loss did not increase DNA damage in the adult hair follicles (Supplementary Fig. 4g). These data suggests that mature progenitors were less susceptible to H3K9me3 loss, but due to ineffective H3K9me3 depletion at this stage the adult phenotypes may have been under-developed.

### H3K9me3 loss alters epidermal development gene programs

**Fig. 4:**
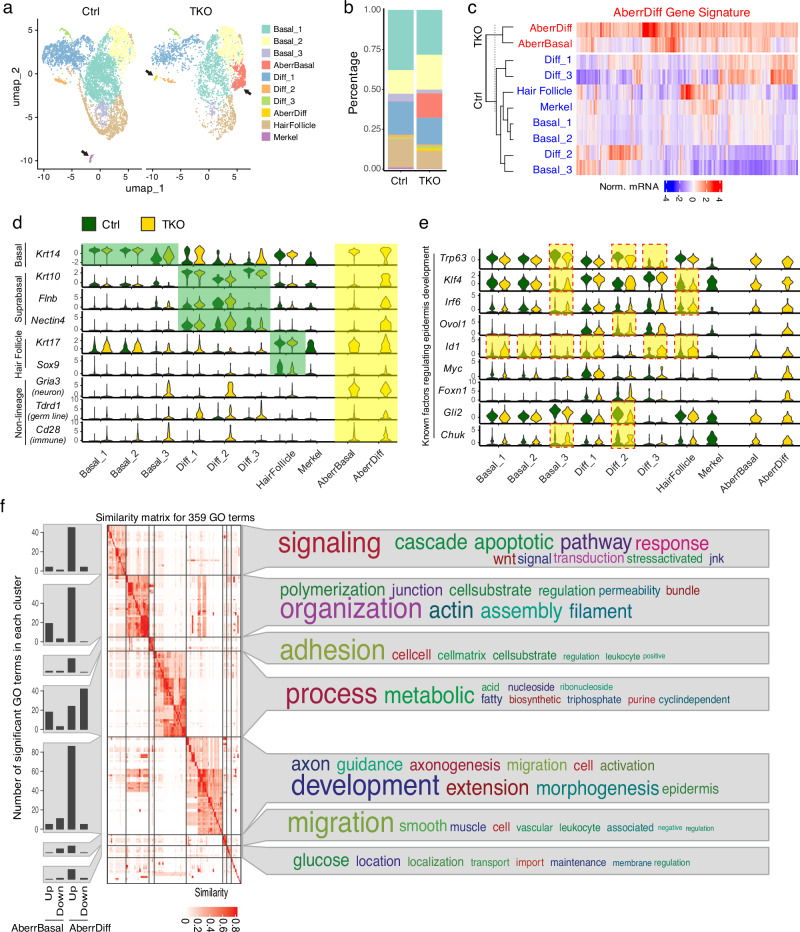
H3K9me3 depletion leads to aberrant cell identity with dysregulated cellular functions essential for epidermis morphogenesis. **a** Visualization of epithelial cell clusters by uniform manifold approximation and projection (UMAP), using scRNA-seq data of sorted epidermal cells from E16.5 embryos. *N* = 2 biologically independent samples per group. Arrows highlight the aberrant populations in TKO and the Merkel cell population in Ctrl. **b** Fraction of each cell cluster in (**a**). **c** Heatmap showing that TKO aberrant populations co-express genes from different keratinocyte subtypes. The AberrDiff gene signature is defined as the top 500 AberrDiff marker genes plus marker genes for other regular populations that are also expressed in AberrDiff. **d** Violin plots highlighting that AberrDiff population express marker genes from diverse lineages, including both epithelial and non-epithelial cells. Green regions highlight properly expressed genes, and yellow regions highlight gene expression in aberrant populations. **e** Violin plots showing dysregulation of factors known to control epidermal development. Yellow regions highlight significantly changed genes in each cluster. Note that some genes are dysregulated in both the aberrant and the regular TKO clusters. **f** Cluster analysis of GO terms enriched in differentially expressed genes when comparing AberrBasal vs. basal, AberrDiff vs. differentiating populations. The heatmap shows the similarity matrix for the 359 GO terms enriched, with each row/column represents one GO term. GO terms are clustered by the “binary cut” method. Overrepresented words from each cluster are shown on the right, and the word sizes reflect frequency of occurrence. Note the enrichment for development, morphogenesis, cytoskeleton organization (actin assembly and filament), cell substrate, and signaling. The full list of GO terms are reported in Supplementary Data 2.

The enrichment of H3K9me3 peaks at developmental genes and putative enhancers (Fig. 1), along with the impaired morphogenesis upon its loss (Figs. 2 and 3), prompted us to investigate whether H3K9me3 regulates gene programs critical for keratinocyte lineage diversification. To this end, we FACS-isolated epidermal cells (EpCam+) from Ctrl and mosaic TKO embryos at E16.5 (Supplementary Fig. 5a) and performed single-cell RNA sequencing (scRNA-seq). Unsupervised clustering following quality control and data filtering grouped 9748 high-quality single cell transcriptomes in distinct expected cell clusters: three basal, three differentiating, hair follicle and Merkel cell (Fig. 4a, b, and Supplementary Fig. 5b–d). The Merkel cell cluster was absent in TKO samples, confirming our IF data (Fig. 2k–m). Intriguingly, two unique TKO-specific clusters emerged, which we termed aberrant basal (aberrBasal) and aberrant differentiating populations (aberrDiff) cells (Fig. 4a, b).

The aberrant TKO populations co-expressed lineage-specific markers normally restricted to distinct epidermal subtypes (e.g., clusters). For example, aberrDiff cells expressed basal (*Krt14*), suprabasal (*Krt10*, *Flnb*), and hair follicle (*Sox9*) markers, along with non-lineage genes, such as *Gria3* (neuronal), *Tdrd1* (germline), and *Cd28* (immune) (Fig. 4c, d). These findings are consistent with co-expression of Krt14, Krt10, and Involucrin in the same basal cells shown by IF (Fig. 3i, j). Unlike the antiviral and DNA damage responses that develop later in adult skin following *Setdb1* knockout via *K14-Cre*^16^, these processes were not significantly altered in our embryonic model with short-term induction via *K14-CreER*^*T2*^ (Supplementary Fig. 6a–h).

Numerous genes previously implicated in epidermis development^33,34^, were mis-regulated not only in the aberrant clusters but also in other TKO clusters (Fig. 4e and Supplementary Fig. 7a). GO enrichment analysis of differentially expressed (DE) genes in aberrant populations relative to regular cell populations revealed functional enrichment for processes including development, morphogenesis, signaling, metabolism, cytoskeletal organization, extracellular matrix, and cell cycle (Fig. 4f and Supplementary Fig. 7b–g). These changes align with TKO phenotypes observed by immunostaining, such as proliferation defects, flattened cell shape, and basement membrane abnormalities (Fig. 3 and Supplementary Fig. 3).

Aside from the aberrant clusters unique to TKO, comparison of clusters common to Ctrl and TKO samples revealed widespread, keratinocyte subtype (or cell cluster) specific transcriptomic changes in programs regulating epidermis morphogenesis, metabolism, cell adhesion, mitosis, chromosome segregation, and many more (Supplementary Fig. 8a, b, Supplementary Data 2 and 3). These TKO populations likely arise from mosaic recombination and variable levels of demethylation and may occupy intermediate states in the aberrant fate trajectory. Notably, analysis of Ctrl scRNA-seq clusters revealed that *Suv39h1* and *Suv39h2* expression was enriched in basal and hair follicle clusters, whereas *Setdb1* was broadly expressed (Supplementary Fig. 8c), consistent with IF results (Supplementary Fig. 2a, c). This differential expression may partially account for the specific transcriptomic responses observed in the different keratinocyte subtypes.

Finally, sub-clustering of hair follicle cells revealed reduced representation of late-stage *Lrig1+ Sox9+* subpopulations and increased representation of *Shh+ Edar+* early placode-stage cells in TKO samples (Supplementary Fig. 8d–f), explaining the impaired hair follicle morphogenesis (Fig. 2h–j).

Together, these findings demonstrate that H3K9me3 regulates epidermal lineage diversification both by repressing non-lineage genes and by orchestrating keratinocyte subtype-specific gene expression programs essential for skin development.

### H3K9me3-regulated genes localize near H3K9me3 peaks

**Fig. 5:**
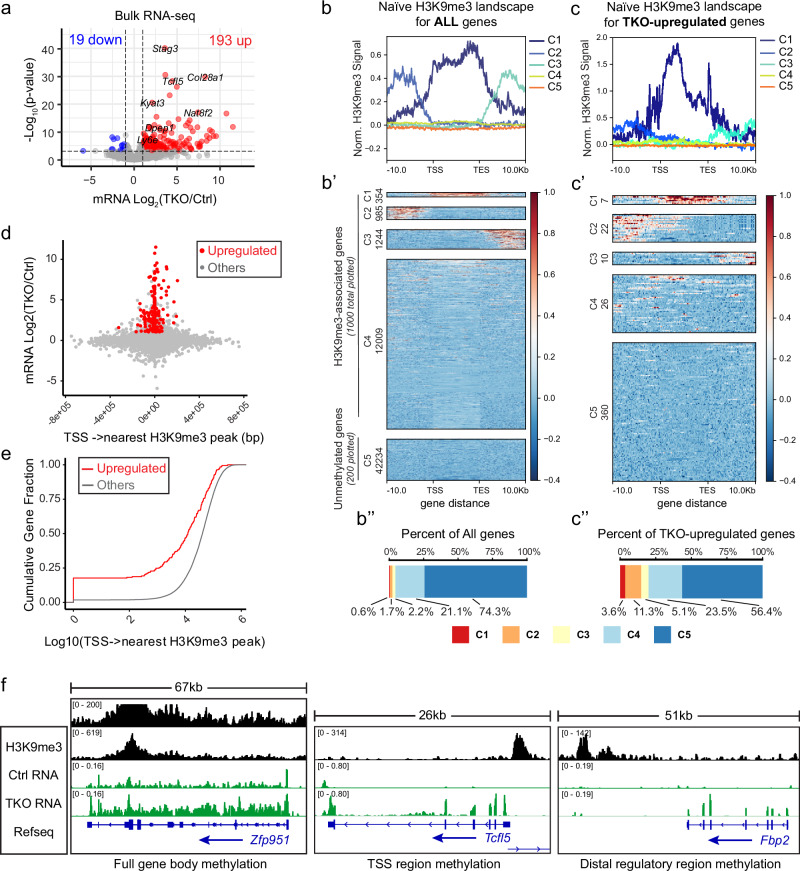
Genes upregulated upon H3K9me3 loss associate preferentially with nearby H3K9me3 peaks. **a** Volcano plot showing differentially expressed (DE) genes between TKO vs. Ctrl by bulk RNA-seq analysis of E16.5 epidermis. DE analysis was performed using DESeq2 based on a two-sided Wald test, with *P* values adjusted for multiple comparisons using the Benjamini–Hochberg method. **b**-**b”** Metaplot (**b**) and heatmap (**b’**) showing naïve H3K9me3 landscape (in E15.5 Ctrl epidermis) on gene body and ±10 kb flanking regions of all genes. Gene body regions are scaled to 10 kb for visualization. Five gene clusters (C1–C5) with distinct H3K9me3 patterns were obtained using k-means method. Numbers immediately left of panels in (**b’**) represent total number of genes in each cluster. The indicated number of genes were randomly selected for visualization. Barplot (**b”**) shows the percentage of all genes belonging to each cluster. **c**-**c’’** Same metaplot (**c**), heatmap (**c’**) and bar graph (**c”**) showing naïve H3K9me3 landscape for TKO-upregulated genes obtained from (**a**). Dot plot (**d**) and cumulative plot (**e**) showing that TKO upregulated genes from (**a**) are generally closer to H3K9me3 peaks when compared to other genes. TSS: transcription start sites. **f** Integrative genomics viewer (IGV) tracks showing examples of genes with different methylation patterns: full gene body methylation (*Zfp951*), TSS region methylation (*Tcfl5*) and distal regulatory region methylation (*Fbp2*). For *Zfp951*, additional scale of 0–200 is shown to highlight large H3K9me3 domains. H3K9me3 signals in all panels represent signals above background noise, with IgG control signals subtracted at corresponding loci.

We next examined how genome-wide H3K9me3 deposition might regulate gene expression in the epidermal lineage. Since H3K9me3 landscapes were profiled by CUT&RUN in bulk FACS-sorted epidermal cells, we performed a matching bulk RNA-seq of Ctrl and TKO cells at E16.5. We captured 212 dysregulated genes, of which 193 were upregulated (Fig. 5a and Supplementary Data 4). These genes were primarily associated with germline, muscle, cardiac, and neuronal programs (Supplementary Fig. 9a), reflecting de-repression of select non-lineage genes. Interestingly, 151 of 193 upregulated genes are associated with “constant” H3K9me3 peaks maintained throughout normal epidermal development (Supplementary Fig. 9b), consistent with roles in non-lineage gene repression (Supplementary Fig. 1h).

To define the naïve H3K9me3 landscape immediately preceding mRNA changes at E16.5, we performed CUT&RUN on Ctrl epidermal cells at E15.5 (Supplementary Fig. 9c). Epic2 peak calling revealed a wide size distribution (Supplementary Fig. 9d), identifying genes associated with either broad domains or narrow discrete peaks (Supplementary Fig. 9e, f). Genes associated with narrow peaks are enriched for development-related GO terms, while genes with broad domains are not (Supplementary Fig. 9g, h), suggesting that narrow peaks are more pertinent to lineage regulation.

Next, we examined H3K9me3 methylation patterns around genes and extrapolated them to the TKO upregulated genes. Clustering analysis across 56,884 annotated gene loci with ±10 kb flanking DNA regions revealed five distinct patterns: broad intragenic H3K9me3 coverage (cluster 1, C1), upstream (C2) or downstream (C3) H3K9me3 broad domains, dispersed H3K9me3 islands (C4), and minimal methylation (C5) (Fig. 5b, b’, and Supplementary Data 5). Similar H3K9me3 patterns were obtained by analyzing ±50 kb flanking regions and genes belonging to each cluster largely overlap with those obtained using ±10 kb flanks (Supplementary Fig. 10a, b, and Supplementary Data 5). C1 and C2 were enriched in GO functions important for transcriptional repression and heterochromatin organization, while C4 genes were associated with developmental pathways including cell fate, metabolism, and morphogenesis (Supplementary Fig. 10c). These analyses revealed defined H3K9me3 patterns associated with specific gene functional groups, suggesting distinct H3K9me3-driven mechanisms may underlie specific tissue functions.

We then analyzed the Ctrl naïve H3K9me3 methylation patterns at E15.5 for genes upregulated in TKO epidermis by E16.5. We found that 43.6% of the bulk RNA-seq TKO-upregulated genes were originally marked by H3K9me3 (C1–C4 clusters) and 20% were in the C1–C3 clusters, a four-fold enrichment compared to genome-wide averages (Fig. 5c-c”). Thus, many genes upregulated upon H3K9me3 loss are likely directly repressed by this mark, while others may involve distal H3K9me3-marked elements, such as long-range enhancers.

To further examine this, we calculated distances between each upregulated gene’s transcription start site (TSS) and its nearest H3K9me3 peak. Upregulated genes had significantly shorter distances than background genes (Fig. 5d, e), a trend mirrored in DE genes from scRNA-seq (Supplementary Fig. 10d). Indeed, about 20% of upregulated genes overlapped an H3K9me3 peak at the TSS (Fig. 5e), covering either the gene body or promoter regions (Fig. 5f, *Zfp951* and *Tcfl5*), indicating potential direct repression of RNA Pol II activity. Other upregulated genes with more distal H3K9me3 peaks (Fig. 5f, *Fbp2*) may be transcriptionally regulated through enhancer silencing.

To directly investigate Pol II activity and regulatory steps at promoters and enhancers, events not captured by the traditional steady-state RNA assays used thus far, we next employed our recently developed PReCIS-seq methodology^26^.

### H3K9me3 loss alters Pol II regulation at select promoters

**Fig. 6:**
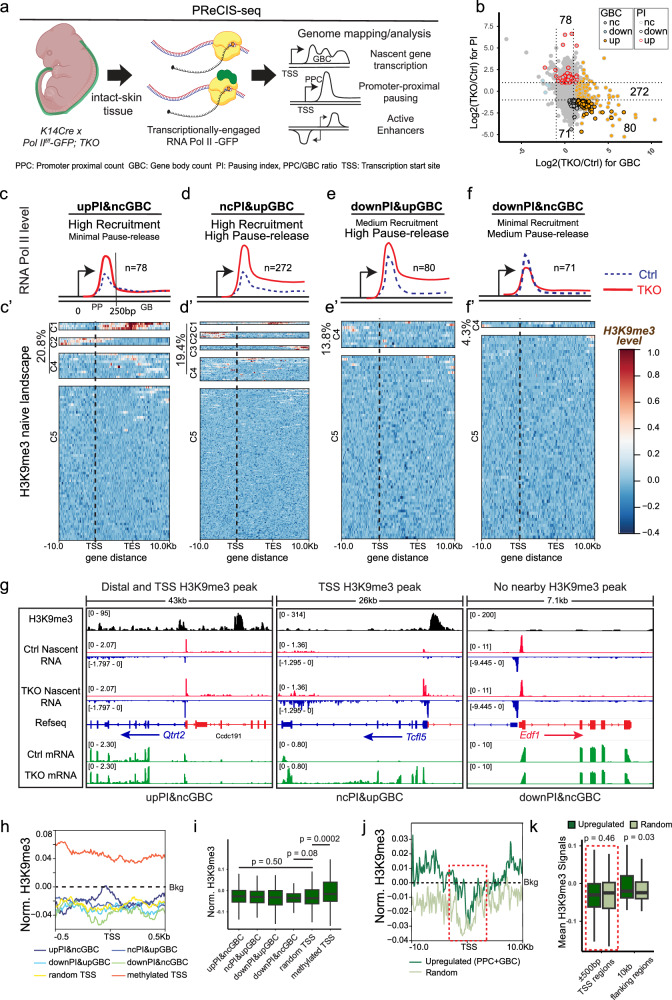
H3K9me3 loss up-regulates transcription at specific genes by increasing RNA Pol II promoter recruitment and pause-release. **a** Schematics showing the concept of PReCIS-seq methodology. For more detailed information, see Chovatiya et al.^26^. **b** Dot plot showing changes of pausing index (PI) versus gene body transcription (GBC) upon TKO. Up: upregulated; down: downregulated; nc: not significantly changed. **c–f** Profile plots of TKO dysregulated genes categorized based on PI vs. GBC variation in (**b**). Engaged-RNA Pol II occupancy in TKO vs. Ctrl is drawn based on PReCIS-seq results from Supplementary Fig. 12a. PI and PPC increase reflect RNA Pol II promoter-recruitment, or transcriptional initiation. PI decrease and GBC increase indicates Pol II pause-release into elongation. Number of genes in each category is indicated on plot. **c’–f’** Heatmap showing H3K9me3 cluster patterns (C1–C5) within genes and ±10 kb flanking regions for gene categories in (**c**–**f**). Note that genes regulated by high Pol II promoter-recruitment associate more with H3K9me3-enriched clusters (C1–C4) compared to genes dominated by pause-release. **g** IGV tracks showing examples of TKO-dysregulated genes with respect to nearby H3K9me3 peaks. **h, i** Metaplot and quantification showing that TKO dysregulated gene categories from **b** are unmethylated in proximal regions flanking TSS (±500 bp). Signals below the dashed line are considered background based on IgG control. *n* = 78, 71, 150, and 150 genes for upPI&ncGBC, downPI&ncGBC, random TSS and methylated TSS, respectively. **j**, **k** Metaplot and quantification showing that TKO-upregulated genes possess more H3K9me3 methylation than random control genes in distal regions (±10 kb) rather than proximal regions (±500 bp) around TSS. For both comparisons, *n* = 232 genes for upregulated group, and *n* = 300 genes for random group. Signals below the dashed lines are considered background based on IgG control. Box plots show the median (center line), interquartile range (box; 25th–75th percentiles), and whiskers extending to the minimum and maximum values no further than 1.5× the interquartile range. Statistical significance was assessed using two-sided unpaired t-tests. Created in BioRender. Bai, C. (2026) https://BioRender.com/omfbxi1.

PReCIS-seq^26^ is a derivative of precision transcriptional run-on and sequencing (PRO-seq)^35^. It can uniquely identify active promoters and enhancers, quantify nascent transcription, and distinguish between Pol II regulatory steps, such as promoter recruitment (initiation) and promoter-proximal pause-release (elongation) of a specific cell lineage in its developmental context (Fig. 6a)^26^. We applied this method to investigate RNA Pol II regulation in embryonic skin keratinocytes upon H3K9me3 loss.

We generated *K14-Cre; Suv39h1*^*f/f*^*; Suv39h2*^*KO/KO*^*; Setdb1*^*f/f*^*; Polr2b*^*f/f*^*-GFP* mice (Supplementary Fig. 11a, b), allowing for Cre-inducible fusion of GFP to the endogenous RBP2 subunit of RNA Pol II, as described^26^. Rapid snap-freezing of embryonic back skin preserves in vivo RNA Pol II location at active transcription sites. Transcription run-on labels the ongoing nascent transcript bound to Pol II with biotin, followed by sequential anti-GFP and streptavidin pull-down to enrich for nascent RNA specifically from keratinocytes^26^. Sequencing these transcripts produces base-pair-resolution profiles of transcriptionally engaged RNA Pol II genome-wide.

PReCIS-seq on E16.5 TKO skin, using HMT-heterozygous embryos as controls (Ctrl), generated epidermis-specific nascent transcription profiles (Supplementary Fig. 11c). The TKO nascent transcriptome was globally more active (Supplementary Fig. 11d), according to spike-in normalized total counts (see “Methods”). We then performed DE analysis on full nascent transcripts and identified 235 dysregulated genes (Supplementary Fig. 11e and Supplementary Data 6). PReCIS-seq data more directly reflect H3K9me3 regulation on transcription and rule out potential confounding effects from indirect or post-transcriptional processes that steady-state (mature) RNAs from RNA-seq cannot exclude. Indeed, bulk RNA-seq and PReCIS-seq data overlap only to a limited extent (Supplementary Fig. 11f). Common upregulated genes are enriched for GO-terms relating to chromosomal segregation and meiosis (Supplementary Fig. 11g).

Next, we examined if H3K9me3 loss impacts RNA Pol II promoter-recruitment (e.g., initiation) vs. proximal-promoter pause-release (e.g., elongation). These two Pol II regulatory steps employ distinct mechanisms and may serve distinct purposes in development^20,36^. Using the ratio of promoter-proximal count (PPC) to gene body count (GBC) from PReCIS-seq, we define the Pol II pausing index (PI), a metric of RNA Pol II stalling ~60 bp downstream of the TSS. Changes in GBC and PI across conditions reveal which Pol II regulatory step, recruitment or pause-release, is impacted (Fig. 6a). When combining genes with significant changes of GBC, PI, or both we identified 451 dysregulated genes (Fig. 6b, Supplementary Fig. 12a, and Supplementary Data 6).

We defined four gene categories based on PI and GBC changes:

1. upPI&ncGBC: increased PI with no significant changes in GBC
2. ncPI&upGBC: no change in PI while GBC increased
3. downPI&upGBC: PI decreased, but GBC increased
4. downPI&ncGBC: PI decreased while GBC did not change

Genes from the first two categories (upPI&ncGBC, ncPI&upGBC) are dominated by high RNA Pol II promoter recruitment and transcription initiation. Notably, upPI&ncGBC displays increased Pol II initiation at promoters but does not display productive release into transcription elongation (Fig. 6c, d). The remaining two categories (downPI&upGBC, downPI&ncGBC) were dominated by promoter proximal pause-release (Fig. 6e, f). Notably, only ncPI&upGBC and downPI&upGBC could be detected as upregulated with bulk RNA-seq data (Supplementary Fig. 12b). The first two gene categories, regulated by RNA Pol II recruitment, showed ~20% enrichment in methylation-rich clusters (Fig. 6c’, d’), and a subset (C1–C2) have promoter methylation, suggesting direct repression. In contrast, the two gene categories regulated by pause-release showed little to no such enrichment over the promoter or gene body regions and were in fact largely unmethylated (Fig. 6e’, f’). Genome browser views confirmed distinct RNA Pol II engagement and methylation patterns among these categories (Fig. 6g and Supplementary Fig. 12c). Overall, H3K9me3 levels near TSS sites were low across all groups (Fig. 6h, i). Instead, genes with strong upregulation in TKO (assessed by GBC + PPC) showed significantly high H3K9me3 levels in regions distal to the TSS (Fig. 6j, k). These data suggest that H3K9me3 mainly affects RNA Pol II transcription initiation at promoters with some marginal and likely indirect effects on Pol II pause release into productive elongation (see “Discussion”).

As a side note, some genes activated in TKO used non-canonical TSSs. These sites were covered by H3K9me3 peaks in the naïve chromatin (Supplementary Fig. 12d, e). Some were annotated as alternative promoters, while others were ectopic. Nonetheless, some of these transcripts were processed into correct mature mRNAs, as seen for the *Fabp4* gene (Supplementary Fig. 12d).

Together, these findings suggest that H3K9me3 may silence a select set of genes through direct promoter repression. When removed, many genes activate transcription via enhanced RNA Pol II promoter-recruitment and transcription initiation. However, only a subset of genes gains elongation competence, implicating additional requirements for Pol II pause-release and productive transcription. A smaller subset of genes is regulated mainly at the pause-release step upon H3K9me3 loss, likely through indirect mechanisms. Importantly, most genes upregulated upon H3K9me3 loss are associated with distal, not proximal, H3K9me3 peaks. This supports a hypothesis where H3K9me3 primarily regulates gene expression via repression of enhancer elements, rather than promoter silencing, a possibility that we will examine next.

### H3K9me3 loss activates enhancers in developing epidermis

Active enhancers generate short, bidirectionally transcribed, unstable RNAs—enhancer RNAs (eRNAs)—which are more predictive of enhancer activity than histone modifications^37,38^. Because eRNAs are difficult to detect via standard steady-state RNA-seq techniques, nascent RNA profiling techniques, such as PRO-seq^35^ are typically used. PReCIS-seq also effectively identifies enhancer activity via bidirectional transcription, and these elements strongly overlaps with histone marks of active enhancers^26^.

**Fig. 7:**
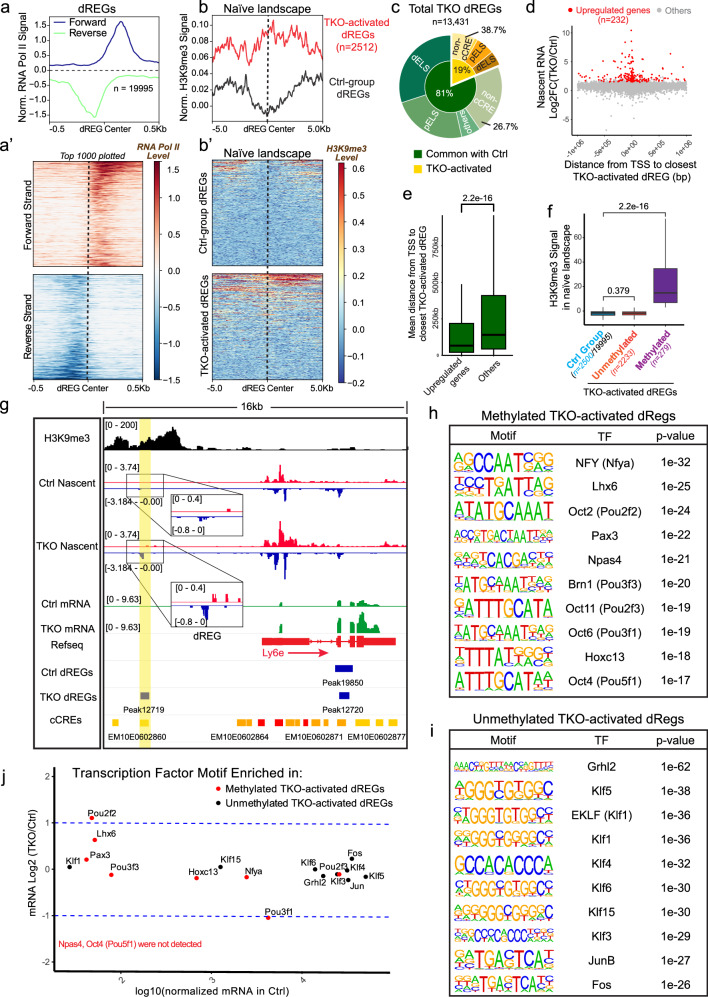
H3K9me3 loss promotes RNA Pol II activity at distal enhancers. **a**, **a’** Metaplot and heatmaps of Ctrl RNA Pol II signals showing bidirectional transcription for dREG elements. Top 1000 out of 19995 dREGs detected in Ctrl are plotted. **b**, **b’** Metaplot and heatmaps showing that subset of TKO-activated dREGs are methylated in the naïve H3K9me3 landscape (13431 dREGs in total; 279 out of 2512 TKO-activated dREGs are methylated), while dREGs from Ctrl (2500 out of 19995 were randomly selected for visualization) are unmethylated. **c** Pie chart showing the percentage of TKO dREGs overlapping ENCODE-defined cCRE elements (predicted enhancers) or non-cCRE representing newly identified enhancers. **d**, **e** TSS of upregulated genes (by PReCIS-seq) are closer to TKO-activated dREGs than other genes. *n* = 232 Upregulated genes and *n* = 37573 other genes. **f** Quantification of H3K9me3 CUT&RUN signal within ±1 kb of dREG peak center, summed for analyses. Two thousand five hundred out of 19995 Ctrl dREGs were randomly selected to match the number of TKO-activated dREGs (*n* = 2512) for statistics. Methylated TKO-activated dREGs (*n* = 279) were identified by testing each TKO dREG against the distribution of background methylation based on Ctrl dREGs. **g** IGV tracks showing the *Ly6e* gene locus. *Ly6e* is upregulated in both mRNA level and nascent transcription level. A TKO-activated enhancer is located upstream of *Ly6e* locus and covered by H3K9me3. Yellow columns highlight the activated dREG regions. List of transcription factor motifs enriched in methylated (**h**) or unmethylated (**i**) TKO-activated dREGs. **j** Expression by bulk RNA-seq for transcription factors (TFs) corresponding to motifs enriched in TKO-activated dREGs, split based on the methylation status of their loci in the naïve chromatin landscape. Note that most TFs are expressed in Ctrl and do not change expression in TKO samples. Box plots show the median (center line), interquartile range (box; 25th–75th percentiles), and whiskers extending to the minimum and maximum values no further than 1.5× the interquartile range. Statistical significance was assessed using two-sided unpaired t-tests.

To define active enhancers from our PReCIS-seq data and correlate it with H3K9me3 loss, we applied dREG, a method developed for detecting bidirectionally transcribing DNA regulatory elements using transcriptional run-on assays^39^ (Fig. 7a, a′). After removing promoter-associated regions, we identified 13,431 distal bidirectionally transcribed elements (denoted as dREGs hereafter). Of these, 2512 (19%) were specific to the TKO, with the remainder also present in Ctrl. Ctrl dREGs generally lacked H3K9me3 signal, whereas many TKO-specific dREGs were originally marked by H3K9me3 peaks in the naïve chromatin landscape of Ctrl samples (Fig. 7b, b′). Over 70% of all dREGs overlapped with annotated candidate cis-regulatory elements (cCREs), supporting their enhancer identity (Fig. 7c). Thus, active enhancers are generally devoid of H3K9me3, and loss of H3K9me3 at a subset of silent enhancers upon TKO leads to their ectopic activation.

To understand whether the enhancers activated by H3K9me3 loss contribute to subsequent transcription activation of nearby genes, we performed gene-enhancer association analysis. Indeed, TKO-upregulated genes, on average, were located closer to TKO-specific dREGs than other genes (Fig. 7d, e). For example, *Ly6e*, a TKO-upregulated gene, lies downstream of a predicted enhancer annotated as cCRE, which was marked by H3K9me3 in the naïve chromatin and subsequently became bidirectionally transcribed in TKO (Fig. 7g). These findings support functional enhancer–promoter relationships and suggest that H3K9me3 loss at specific enhancers can trigger their activation along with that of adjacent genes.

To further understand how TKO-specific enhancers are activated, we asked what transcription factor (TF) motifs they harbor. TKO-specific enhancers could be divided into two major classes: a small subset (~10%) that was originally methylated in the naïve chromatin landscape, and a larger group that was largely unmethylated (Fig. 7b′, f). This distinction strikingly correlated with TF motif enrichment. Methylated enhancers were enriched for NFY and POU-domain TF motifs not found in unmethylated enhancers (Fig. 7h and Supplementary Fig. 13a). In contrast, unmethylated enhancers showed motif compositions similar to enhancers active in controls (Fig. 7i and Supplementary Fig. 13b), including motifs for GRHL2 and KLF factors—TFs known to regulate keratinocyte identity^40–42^. Most of the TFs identified in the analysis are expressed in both TKO and Ctrl keratinocytes (Fig. 7j), suggesting that their activity at enhancers is normally restricted by H3K9me3 deposition. Thus, H3K9me3 covers a subset of silent enhancers with distinct TF motifs and physically blocks TF access to these loci. In contrast, inactivity of unmethylated enhancers in naïve chromatin may not be directly governed by H3K9me3 deposition at these sites; their activation in TKO might be attributed to indirect effects caused by H3K9me3 loss at other loci (see Discussion).

Lastly, we inspected the function of genes regulated by H3K9me3-covered enhancers or promoters. For enhancers, we examined dysregulated genes residing within ±10 kb from TKO-activated dREGs. Via GO analysis complemented by literature search, we found that many of these genes regulate important cellular processes, and some have known functions in organogenesis and cell differentiation (Supplementary Fig. 13c, d). We also checked genes directly regulated by promoter methylation and found most of them have known lineage regulatory functions (Supplementary Fig. 13e). Among them, Atp7ip is a well-studied Setdb1 co-factor that regulates lineage differentiation in many systems, including hematopoiesis^43^, suggesting a negative feedback loop.

Together, these results indicate that H3K9me3 both directly and indirectly silences a subset of enhancers in epidermis, thereby safeguarding lineage-specific gene expression. Upon H3K9me3 loss, these enhancers become activated and contribute to aberrant transcriptional programs during epidermis morphogenesis.

## Discussion

**Fig. 8:**
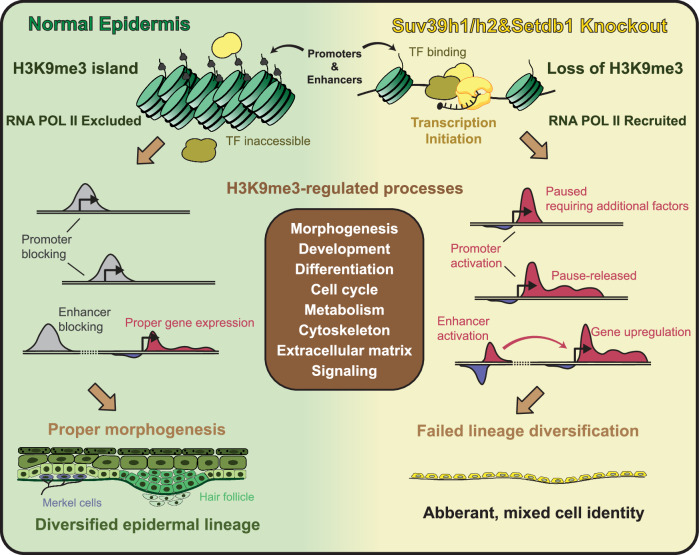
H3K9me3 regulates epidermis morphogenesis by repressing RNA Pol II activity on promoters and enhancers. Model illustrating the role of H3K9me3 in epidermal lineage regulation. In normal epidermis (left), H3K9me3 contributes to heterochromatin formation at lineage-related promoters and enhancers, which blocks binding of transcription factors (TFs) and RNA Pol II recruitment. This represses expression of lineage-inappropriate genes and ensures proper epidermis morphogenesis. By contrast, in Suv39h1/Suv39h2/Setdb1 triple knockout epidermis (right), loss of H3K9me3 opens the chromatin, allowing TF binding and RNA Pol II transcription initiation, which leads to aberrant activation of both promoters and enhancers, eventually causing failed lineage diversification and mixed cell identity. The cellular processes regulated by H3K9me3 is summarized in the brown box. Created in BioRender. Bai, C. (2026) https://BioRender.com/4enj4zw.

In this study, we showed that the heterochromatin mark H3K9me3 is dynamically re-distributed genome-wide during epidermis development and is essential for skin morphogenesis. By genetically ablating Suv39h1, Suv39h2 and Setdb1, the three histone methyltransferases (HMTs), which deposit H3K9me3, we uncovered its essential role in epidermal lineage diversification. Loss of H3K9me3 in epidermis impairs the formation of specialized keratinocyte-derived cell types, including hair follicle cells, Merkel cells, and suprabasal differentiated cells, resulting in aberrant cell fates with mixed cell identity. H3K9me3 regulates gene expression programs vital for skin development and tissue morphogenesis, including specific pathways governing differentiation, cell cycle, signaling, metabolism, extracellular matrix organization, and cytoskeletal dynamics, which were disrupted in its absence. Mechanistically, H3K9me3 loss selectively impairs RNA Pol II recruitment to promoters (initiation) and divergent transcription at developmentally regulated enhancers, with minimal and rather indirect effects on Pol II promoter-proximal pause release (elongation). These findings demonstrate that H3K9me3 is indispensable for mouse skin organogenesis, acting through modulation of RNA Pol II dynamics in a lineage-specific manner (Fig. 8).

Although H3K9me3’s role in early embryogenesis is well-established, particularly before gastrulation in both *Drosophila* and mice^13,44^, its function in late development during organogenesis remained poorly defined until now. Prior to this study, complete knockout of all three H3K9me3 HMTs in mice had only been achieved in the endoderm, prior to organ formation, revealing that H3K9me3 represses non-lineage genes^19^. In *C. elegans*, combined deletion of *met-2* and *set-25* (homologs of *Suv39h1/2* and *Setdb1*) eliminates H3K9 methylation without impairing tissue development during embryogenesis, with only mild effects on adult muscle integrity^6,45,46^. In contrast, loss of H3K9me3 in our model caused changes in lineage-specific gene expression with pronounced defects in epidermis morphogenesis. Thus, H3K9me3’s developmental role in organogenesis likely evolved to better support the greater cellular complexity of mammals.

Long-term deletion of individual HMTs in mouse tissues, including in skin via constitutive *K14-Cre*, eventually induces genomic instability, apoptosis, and inflammation later in the adult tissues, largely due to activation of transposons^15,16,18,47^. In stark contrast, our inducible embryonic knockout model (*K14-CreER*^*T2*^) of the three H3K9me3 HMTs does not induce overt DNA damage, apoptosis, or inflammation in the epidermis within the short experimental timeframe studied. Instead, complete H3K9me3 loss in embryogenesis induces immediate disruption of gene expression programs that regulate key processes required for epidermis morphogenesis and barrier formation. Many of these H3K9me3-regulated genes (e.g., *Trp63, Klf4,* and *Id1*) are well-known indispensable regulators of keratinocyte lineage progression^33,34^, consistent with the observed defects in the triple knockout skin. Thus, H3K9me3 is essential for late-stage mammalian development, supporting organ formation and organismal survival.

The need to delete all three HMTs to fully deplete H3K9me3 underscores partial redundancy and possible cooperativity among Suv39h1, Suv39h2, and Setdb1. Neither *Setdb1* knockout under *K14-Cre* nor *Suv39h1/Suv39h2* double knockout alone produces an epidermal phenotype^9,16^. Only the combined loss of all three HMTs in our study disrupted skin organogenesis, likely due to the broader effects on gene expression programs. Similar synergistic effects have been reported in *Drosophila*, where double mutants for Setdb1 and Suv39h homologs exhibit stronger phenotypes than single mutants^44^. In our study, in addition to de-repressing non-lineage genes (e.g., germline, neuronal, and immune), H3K9me3 loss leads to dysregulation of keratinocyte subtype-specific programs, resulting in mixed cell identities within the lineage. Aberrant TKO cells co-activate gene expression programs normally restricted to distinct keratinocyte cell subtypes, such as basal, differentiated, and hair follicle. Furthermore, even when these keratinocyte sub-types retain their identity, they still show cluster-specific gene dysregulation. These observations could reflect differences in HMT expression (with Suv39h1/2 enriched in basal cells and Setdb1 expressed in both basal and suprabasal keratinocytes), distinct transcription factor context, and indirect effects from altered cell-cell communication. The functional interplay among these HMTs warrants deeper exploration.

While H3K27me3, another repressive histone mark deposited by PRC2, has been more extensively studied during organogenesis^21,48^, its role differs from that of H3K9me3. H3K27me3 depletion in keratinocytes causes mild tissue disorganization, premature barrier formation, and ectopic Merkel cells, yet lineage diversification still occurs, including the formation of hair follicles^49^. By contrast, H3K9me3 loss blocks the formation of keratinocyte derivatives and barrier function. This indicates nonredundant developmental roles between the two marks, in line with their distinct genomic localizations^50^ and differential gene targets. Specifically, H3K27me3 primarily silences terminal differentiation genes^51^, while H3K9me3 controls broader programs including metabolism, ECM organization, cell cycle regulation, as well as non-lineage genes, highlighting the distinct functions of the two marks in tissue development.

We also found that epidermal progenitors are relatively hypomethylated before E12.5, prior to morphogenesis, resembling pre-implantation embryos and embryonic stem cells prior to lineage diversification^7,52,53^. Low H3K9me3 levels have similarly been reported in quiescent tissue stem cells, such as in hair follicles, muscle, and T cells, where chromatin openness supports multipotency and lineage plasticity in wound healing^54–58^. As development proceeds, H3K9me3 accumulation correlates with chromatin restriction and lineage commitment^2,6,19^. Thus, the broad potential of epidermal progenitors before E12.5 might be restricted by elevated and dynamic H3K9me3 thereafter, which would subsequently guide fate specification of epidermal lineage subtypes.

H3K9me3 acts as a transcriptional repressor for both genes and transposons^2,6,59^ (see “Introduction”), but its specific influence on RNA Pol II regulatory steps  has not been addressed in depth. Key Pol II regulatory steps include promoter-recruitment (initiation), largely driven by developmental transcription factors at promoters or enhancers, and pause-release (elongation) from the proximal-promoter site 30–60 bp downstream of TSS, which depends on factors, such as NELF, pTEFb, and BRD4^36,60^. These steps operate through distinct mechanisms and serve different roles in developmental gene control. For instance, some genes initiate Pol II transcription, but halt elongation downstream the TSS, awaiting pause-release signals that enable rapid and synchronous activation across large fields of cells^60^. In cell culture, H3K9 demethylation and histone acetylation promote pause-release^61,62^. However, Pol II dynamics are highly sensitive to tissue dissociation and cell culture conditions^26^, underscoring the need for analysis in the intact tissue environment. Using PReCIS-seq, we profiled transcriptionally engaged Pol II in developing epidermis. We found that H3K9me3 loss enhanced Pol II recruitment at a subset of silent promoters and enhancers. Many genes showed increased initiation without elongation, suggesting a lack of appropriate pause-release signals at these specific genes. While a significant fraction of promoter-recruitment-associated genes harbor H3K9me3 islands on or around their TSS, pause-release-associated genes mostly lacked H3K9me3 at promoters, pointing to indirect regulation by distal enhancers.

Indeed, most genes activated upon H3K9me3 loss in epidermis harbor distal H3K9me3-marked islands overlapping predicted enhancers. We show that many of these enhancers gain de novo divergent Pol II transcription, a hallmark of enhancer activation^37,38,63^. Interestingly, only a subset of TKO-activated enhancers is H3K9me3-marked in the naïve chromatin. The mechanisms conferring specificity to enhancer H3K9me3 deposition is still under investigation. Here, we show that the methylated (but not un-methylated) enhancers activated upon H3K9me3 loss tend to contain specific motifs for POU and NFY transcription factors. Notably, a POU factor, Oct4, which is best known for activating pluripotency genes, can also recruit Setdb1 to silence lineage-inappropriate genes like trophoblast lineage genes in embryonic stem cells^64^. It is possible that keratinocyte-expressed POU and NFY factors (e.g., Oct2, Oct6, and Oct11, Fig. 7) can recruit H3K9me3 HMTs to repress specific enhancers in a context-dependent manner or activate them when HMTs are absent.

Given the well-documented nascent RNA-dependent mechanisms of H3K9me3 heterochromatin nucleation^65^, it is also plausible that eRNAs produced from these enhancers may contribute to H3K9me3 deposition in a sequence-specific negative feedback loop. Furthermore, the recognized contribution of transposable elements to enhancer sequences^66^ may bridge silencing of transposons and developmental enhancers by H3K9me3^66,67^. Remarkably, many enhancers activated in TKO epidermis lack H3K9me3 in the naïve chromatin and may be indirectly regulated by H3K9me3. This may occur via reorganization of long-range chromatin interactions upon H3K9me3 loss, as shown in studies of CTCF and chromatin looping^68^.

In summary, this study reveals that H3K9me3 is essential for epidermis morphogenesis by enabling lineage diversification and repressing inappropriate lineage and non-lineage genes. Mechanistically, H3K9me3 acts by limiting RNA Pol II recruitment and transcription initiation at both promoters and enhancers, with minor effects on pause-release. Its loss destabilizes transcriptional control, blocking the formation of key keratinocyte cell subtypes and impairing the epidermal barrier, ultimately resulting in complete failure of skin development. These findings establish H3K9me3 as a critical developmental epigenetic regulator of mouse organogenesis.

## Methods

### Ethics

All mouse work was performed in accordance with the Cornell University Institutional Animal Care and Use Committee (IACUC) guidelines (protocol no. 2007-0125).

### Mice

Mice were maintained under specific pathogen-free conditions in a temperature- and humidity-controlled facility (22 ± 1 °C, 50 ± 5% humidity) on a 14 h light/10 h dark cycle (lights on at 06:00 and off at 20:00), with ad libitum access to food and water.

The mouse line carrying *Suv39h1*^*flox/flox*^*; Suv39h2*^*KO/KO*^*; Setdb1*^*flox/flox*^ was a kind gift from Dr. Kenneth Zaret^19^.

*K14-Cre* and *K14-CreER*^*T2*^ transgenic mouse lines have both been actively maintained in the Tumbar laboratory and were originally gifted by Elaine Fuchs laboratory (Rockefeller University) and Pierre Chambon laboratory (Collège de France)^29^, respectively. The K14-H2BGFP line was created by Tudorita Tumbar and has been actively maintained in the Tumbar laboratory^69^. The *Polr2b*^*f/f*^*-GFP* line was generated in the Tumbar lab^26^.

Mice are crossed to make specific models described in Fig. 2a and Supplementary Fig. 11a and maintained on a mixed genetic background. Adult mice from PD36-PD72 and embryos from E10.5–E18.5 were used for experiments as described in the figures and figure legends.

To induce knockout for *K14-CreER*^*T2*^*; TKO* embryos, breeding pairs were set up in the late afternoon, and females were checked for mating plug in the following days. The day plug was found is denoted E0.5. Tamoxifen was prepared in corn oil at 10 mg/mL with 5mg/mL progesterone, and injected via i.p. route to pregnant females at a daily dosage of 100 μg/g body weight 3 consecutive days with various schemes starting at E9.5 and ending at E14.5, as indicated in Fig. 2 and Supplementary Data 1. Females were euthanized and embryos collected at various time points as indicated in the results and figure legend.

### Barrier assay

X-gal barrier assay was performed as previously described^70,71^. Briefly, E18.5 embryos were rinsed in PBS and tail snips were collected for genotyping. Then, the embryos were placed into staining solution (1 mg/mL X-gal, 1.3 mM MgCl_2_, 100 mM NaH_2_PO_4_, 3 mM K_3_Fe(CN)_6_, 3 mM K_4_Fe(CN)_6_, 0.01% sodium-deoxycholate, 0.2% NP-40) and incubated for approximately 2 h or until the typical blue color developed, indicative of barrier breach.

### H&E staining

OCT-embedded samples were cryo-sectioned for H&E staining. Sections were fixed in 4% paraformaldehyde for 10 min at room temperature (RT) and then washed in PBS. Sections were then sequentially stained with hematoxylin (Gill’s method, #3, Polysciences) for 5 min and eosin (Eosin-Y intensified, Fisher scientific) for 2 min at RT, followed by mounting in home-made mounting medium (90% glycerol in 1 M NaCO_3_). Images were taken with a Nikon microscope (model ECLIPSE Ni-U) with ZEN v3.0 software.

### Immunofluorescence staining

Immunofluorescence staining (IF) was performed following a standard protocol. Briefly, OCT-embedded samples were cryo-sectioned and fixed in 4% paraformaldehyde for 10 min at room temperature. Sections were then washed in PBS, followed by blocking and permeabilization in blocking buffer (1% BSA, 2% gelatin, 0.2% Triton X-100, 2.5% (v/v) normal goat serum and 2.5% (v/v) normal donkey serum in PBS) for 1 h at RT. Blocking solution was then aspirated, and samples were incubated with primary antibodies at 4 °C overnight. Sections were then washed with PBST (0.1% Triton X-100 in PBS) and incubated with secondary antibodies at RT for 4 h, or at 4 °C overnight. Then, the samples were sequentially washed with PBST and PBS and then mounted with homemade mounting medium, antifade (2.5 mg/mL p-phenylenediamine and 90% glycerol in PBS). Sections were then stored in −20 °C and imaged within 2 weeks. Imaging was done using a fluorescent microscope (Leica DMI6000B) with the Leica Application Suite X software (v3.9.1.28433). All downstream imaging processing and quantification were performed using Fiji (v1.54p)^72^.

Antibodies used for IF include: H3K9me3 (Abcam, cat. ab8898, 1:10,000, used for most experiments), H3K9me3 (Cell Signaling Technology, cat. 5327T, 1:1000, used when co-staining with other rabbit primary antibodies), Krt14 (Biolegend cat. 906004, 1:20,000), Krt10 (Abcam, cat. ab9026, 1:2000), Krt8 (Biolegend cat. 904804, 1:1000), Integrin-α6 (BD Pharmingen, cat. 555734, 1:2000), P-Cadherin (R&D Systems, cat. AF761, 1:1000), Involucrin (Invitrogen, cat. PA5-104453, 1:1000), Laminin(α1) (1:500, Sigma-Aldrich, cat. L9393), BrdU (Abcam, cat. ab6326, 1:1000), cleaved caspase-3 (R&D Systems, cat. AF835, 1:2000), Suv39h1 (Invitrogen, cat. 702443, 1:500), Setdb1 (Protein-tech, cat. 11231-1-AP, 1:1000), Ki67 (Invitrogen, cat. 14-5698-82, 1:5000), HP1α (Invitrogen, cat. MA5-32018, 1:1000), AE13 (ImmuQuest, cat. IQ292, 1:200), γH2Ax (Abcam, cat. ab2893, 1:1000), TNFα (Invitrogen, cat. PA5-19810, 1:200), Cox2 (Cell Signaling Technology, cat. 12282T, 1:500), GFP (Abcam, cat. ab13970, 1:1000)

All antibodies were diluted in blocking buffer before use.

### EdU proliferation assay

EdU (Invitrogen, cat. C10337) was dissolved in PBS at 1 mg/mL working concentration and injected via i.p. route into pregnant females at 15 μg/g body weight 2 h before sacrifice and embryo collection. The embryos were embedded in OCT and cryo-sectioned. EdU staining was performed using Click-iT™ EdU Cell Proliferation Kit (Invitrogen, cat. C10337) according to the manufacturer’s instructions.

### TUNEL assay

TUNEL assay was performed using Click-iT™ Plus TUNEL Assay Kits for In Situ Apoptosis Detection (Invitrogen, cat. C10618) following the manufacturer’s instruction. Both DNase I-treated samples (following procedure provided in Click-iT^TM^ Plus TUNEL Assay Kit) and UVB-irradiated samples (collected 24 h post UVB irradiation at 180 mJ/mm^2^) were used as positive control each time the procedure was performed.

### Whole mount staining

Whole mount staining procedure for embryos was adapted from previously published work^73^. Briefly, dorsal skin was dissected from embryos and cut into 3 mm × 3 mm squares, followed by a brief rinse in PBS. Tissue pieces were then fixed in 4% PFA at 4 °C for 1 h, washed in PBS and then permeabilized and blocked in blocking solution (0.1% Triton X-100, 2% normal goat serum/normal donkey serum, 1% BSA and 0.02% NaN_3_ in PBS) at 4 °C overnight. After blocking, tissue was incubated with primary antibodies for 72 h at 4 °C, followed by washing in 0.1% PBST at 4 °C overnight. Then the tissue was incubated in secondary antibodies for 48 h at 4 °C, again followed by wash in 0.1% PBST overnight at 4 °C. Skin pieces were then sequentially incubated with an ethanol gradient from 50%, 70%, 100% and another 100% at RT, 1 h incubation for each. At the end, tissue was cleared by incubating with ethyl cinnamate (Sigma-Aldrich, 112372-100G) for 1 h at RT. All incubations were performed on a rocker, and all antibodies were diluted in blocking solution with 0.02% NaN_3_.

### Fluorescence-activated cell sorting

The procedure for sample preparation and fluorescence-activated cell sorting (FACS) was adapted from previous studies^74^. Briefly, dorsal skin was dissected from E12.5, E14.5, and E16.5 embryos for preparing single cell suspension. When applicable, the rest of the embryos were kept for genotyping and immunofluorescence to check knockout efficiency. For E12.5 embryos, the epidermis is only a single layer of cells, so larger tissue chunks were dissected from K14-H2BGFP+ embryos for downstream processing. Dorsal skin tissue was digested at 4 °C overnight in collagenase solution (0.1% collagenase type I, 0.1% collagenase type II, 1.25 mM CaCl_2_, 1.25 mM MgCl_2_ in HBSS). The following day, tissue/cells were rinsed with 500 mg/L EDTA once, followed by digestion with trypsin solution (0.25% in Mg^2+^/Ca^2+^ free HBSS) at 37 °C for 10 min. Digestion was stopped by adding 2 folds volume of 15% FBS. Tissue was then dissociated by pipetting, and the resulting single cell suspension was sequentially forced through 70 and 40 μm cell strainers. Cells were washed and eventually resuspended with FACS buffer (5% FBS in Mg^2+^/Ca^2+^ free HBSS). Cells were stained for Cd49f-BV421 (Biolegend cat. 313624) and Epcam-APC (Biolegend cat. 118214) to label basal cells. Propidium Iodide (PI) was used for viability monitoring. Cell sorting was performed using Sony MA900 cell sorter (with v3.1.2 software) at Cornell Biotechnology Resource Center (BRC) FACS facility. FACS data were analyzed with FlowJo v10.10.0 (BD Biosciences).

### CUT&RUN and library preparation

CUT&RUN experiments were performed following the previously published protocol^75^ (also available at protocols.io). Three Cre- Ctrl embryos at E15.5 were processed for FACS to obtain a single-cell suspension of basal cells. Approximately 120,000 cells were sorted from each embryo and divided into two aliquots: 80,000 cells for H3K9me3 assays and 40,000 cells for IgG as controls. Cells were then bound to Concanavalin A beads (Cell Signaling Technology, Cat#93596), incubated with primary antibodies (rabbit αH3K9me3, Abcam, Cat#ab8898; rabbit αIgG, Cell Signaling Technology, Cat#3900), Protein A/G MNase (EpiCypher, Cat#15-1016), underwent chromatin digestion, and the released DNA fragments were extracted using phenol chloroform methods. Then NEBNext Ultra II DNA Library Prep kit (Cat#E7103S) was used to prepare sequencing library, with NEBNext Multiplex Oligos for Illumina (Index Primers Set 1) (Cat# E6440), following the manufacturer’s instructions. Resulting library were then quality controlled by fragment analysis and sequenced on the NovaSeq 6000 platform with 2 × 150 bp paired-end option.

For E12.5, E14.5 and E16.5 samples, CUT&RUN was performed separately with kits from EpiCypher (Cat# 14-1048) following the manufacturer’s instructions. The downstream library preparation is the same as above except that NEBNext Multiplex Oligos for Illumina (Dual Index Primers Set 1) (Cat# E7600S) was used. The library was sequenced on NovaSeq X Plus series.

### PReCIS-seq and library preparation

PReCIS-seq was performed following our published procedure by Chovatiya et al.^26^. Briefly, dorsal skin tissue was dissected from embryos and flash frozen immediately. The rest of embryos were used for genotyping and immunofluorescence to confirm labeling and knockout efficacy. Three embryos of the same sex were pooled together for each sample. The frozen skin tissue was then pulverized into fine powder using the Cellcrusher (available from cellcruhser.com). The douncing procedure described for adult skin was skipped, and the pulverized tissue powder was directly resuspended in NUN buffer. Before further processing, flies (*Drosophila Melanogaster*) were pulverized similarly and added into embryo samples as spike-in to account for sample-to-sample variations, and the amount of fly tissue added was measured to be proportional to skin tissue weights. We then strictly followed the procedure described by Chovatiya et al. for the rest of PReCIS-seq, including chromatin isolation, chromatin fragmentation and run-on, immunoprecipitation with GFP antibody (Abcam cat#ab290), isolation of nascent transcripts, 3′ and 5′ adapter ligation, reverse-transcription, PCR amplification and final clean-up. The final library was quality controlled by fragment analysis and sequenced on NovaSeq 6000 with 2 × 150 bp paired-end option.

### Single-cell RNA-seq and data analysis

Single-cell RNA sequencing was performed by the Cornell BRC genomics facility. Briefly, approximately 10k cells were sorted from each embryo, 2 embryos from each genotype, followed by library preparation using Chromium Single Cell 3′ Reagent Kit v3 from 10x Genomics, targeting 5000 cells per sample. The final library was sequenced on the NovaSeq X platform with 2 × 150 bp paired-end option.

The raw data were mapped to the mouse genome (GRCm39) using Cell Ranger (v9.0.1) from 10x Genomics. All downstream analyses were then performed in R using Seurat (v5.4.0)^76^, including low-quality cell filtering, contaminating cell removal, data scaling, dimensionality reduction, cell clustering and UMAP projection. Contaminating cells were removed based on their established markers, including fibroblast (*Col1a1*, *Pdgfra*), immune cells (*Spi1*, *Ptprc*) and melanocytes (*Dct*, *Mitf*), and the remaining keratinocytes were used for further analyses. Integration of samples (2 Ctrl samples and 2 TKO samples) were performed using Harmony^77^. Marker genes for each cluster were identified using FindConservedMarkers function and correlated with known keratinocyte marker genes to assign meaningful identity. Differentially expressed genes between Ctrl and TKO were identified using FindMarkers function.

### Bulk RNA-seq and data analysis

Bulk RNA-seq was performed by Cornell TREx facility following standard procedures. Briefly, total RNA was extracted from sorted basal cells using TRIzol following manufacturer’s instruction. Quality of RNA was checked by fragment analysis. Depletion of rRNA was then performed using NEBNext rRNA Depletion Kit, followed by library preparation using NEBNext Ultra II RNA Library Prep Kit. Paired-end sequencing was performed on NovaSeq 6000 platform.

The obtained reads were processed with fastp (v0.23.4)^78^ for adapter trimming, after which the trimmed reads were mapped to the GRCm39 genome using STAR (v2.7.11b)^79^. The count matrix obtained were then analyzed with DESeq2 (v1.50.2)^80^ following the standard workflow to find differentially expressed genes.

### CUT&RUN data analysis

The raw data of CUT&RUN were first trimmed with Trimmomatic (v0.39)^81^ and then mapped to the GRCm39 mouse genome using Bowtie2 (v2.5.1)^82^. For visualization in Integrative Genomics Viewer (IGV v2.17.2), bamcoverage from Deeptools (v3.5.5)^83^ was used to create bigwig files with CPM normalization. For peak analysis, Epic2 (v0.0.52)^28^ was used for peak calling, and the generated bed files were imported into R for further analysis. For dynamic peak analysis, Epic2 peaks were called from E12.5, E14.5, and E16.5 samples, which were then merged to create a custom reference. H3K9me3 signal was counted using Deeptools “multiBamSummary” function, and the resulting count table was imported in R for DESeq2 analysis. ChIPseeker v1.46.1 was used for peak type annotation. For methylation pattern cluster analysis, the H3K9me3 signal tag matrix was obtained using Deeptools “ComputeMatrix” function, and then k-means clustering was performed in R. Deeptools was also used for most heatmaps and profile plots, and bedtools (v2.29.2)^84^ was used for distance analysis. For H3K9me3 signal quantification across specific genomic regions, the IgG signal was subtracted from the H3K9me3 signal using bamCompare (replicates merged), and then the map function from bedtools was applied for quantification.

### PReCIS-seq data analysis

The raw data were first trimmed with fastp to remove adapter sequences. Then Bowtie2 was used to remove rRNA reads by mapping the reads onto the rDNA genome. The remaining unmapped reads were then mapped to a mixed mouse/fly genome (GRCm39 and dm6) using STAR. UMI-based deduplication was performed using umitools (v1.1.4)^85^. Deeptools was then used to generate bigwig files for visualization in IGV genome browser, with scaling factor calculated from spike-in read number. For PPC and GBC counting, bam files were imported into R using BRGenomics (v1.12.0)^86^ with import_bam_PROseq, and subsequently counted using getCountsByRegions. Differential pausing analysis and differential expression analysis were both performed using DESeq2.

For enhancer analysis, dREG (v20200515)^39^ was used to call divergently transcribed elements as dREG peaks. The peak files were then imported into R for downstream analysis. Peaks overlapping promoter region (based on Refseq) were removed from all analyses. The file for cCRE was downloaded from ENCODE. Bedtools was used for counting H3K9me3 signal over dREG peaks. Motif analysis was performed using Homer (v4.11)^87^.

### Gene ontology analysis

Gene ontology (GO) analysis on gene groups of interest was performed using clusterProfiler (v4.18.2)^88^. Top unique GO terms were selected for visualization, and the full GO term lists were reported in Supplementary Data 2. GO term similarity analysis was performed using simplifyEnrichment (v2.4.1)^89^.

### Statistics and reproducibility

Statistical analyses were performed using GraphPad Prism (v10.6.1) or R (version 4.5.2). All TKO samples were validated for H3K9me3 depletion by IF staining before used for any experiments. All experiments were repeated with at least three biological replicates, and representative data are shown. For sequencing experiments, at least one female and one male sample were used for both the Ctrl and TKO groups to minimize sex-related effects. Unpaired Student’s *t* test was used to compare two groups, and one-way analysis of variance (ANOVA) was used for multiple comparisons of three or more groups. *P*-value (adjusted *p*-values if involves multiple comparisons) smaller than 0.05 was considered statistically significant and reported in figures and figure legends.

### Reporting summary

Further information on research design is available in the Nature Portfolio Reporting Summary linked to this article.

## Supplementary information

Supplementary Information

Description of Additional Supplementary Files

Supplementary Data 1

Supplementary Data 2

Supplementary Data 3

Supplementary Data 4

Supplementary Data 5

Supplementary Data 6

Reporting Summary

Transparent Peer Review File

## Source data

Source Data

## Data Availability

All genomic data, including bulk RNA-seq, scRNA-seq, CUT&RUN and PReCIS-seq data, have been deposited in NCBI’s Gene Expression Omnibus (GEO)^90^ and are freely accessible through GEO Series accession number GSE300774. Single-cell RNA-seq data for Setdb1 knockout^16^ is available from GEO via GSE233240. Source data are provided with this paper.

## Code availability

Scripts for custom code used for data analyses and figure generation in this study are available at GitHub (https://github.com/ChrisBai-Epi/H3K9me3_EpiDevelopment_NatComm2026) and archived at Zenodo (10.5281/zenodo.19358131). This repository corresponds to the version of code used to generate all results presented in this study.
