# Supplementary material for: H3K9me3 controls epidermis morphogenesis by regulating RNA Pol II dynamics at developmental promoters and enhancers
Source: Nat Commun. 2026 May 15;17:6485. doi: 10.1038/s41467-026-73308-5 (PMC13377180; doi:10.1038/s41467-026-73308-5)
Supplement: Supplementary file 1 — Supplementary Information [file 41467_2026_73308_MOESM1_ESM.pdf]

Supplementary Information

H3K9me3 controls epidermis morphogenesis by regulating RNA Pol II dynamics at developmental promoters and enhancers

Chris Ke Bai (白珂)[1], Gopal Chovatiya[1], Emily Janine Pollack[1,2], Yu-Ching Liao[1], Ashley Nayeon Kim[1] and Tudorita Tumbar[1,#]

[1] Department of Molecular Biology and Genetics, Cornell University, Ithaca, NY, USA

[2] Present address: Department of Pathology, Yale School of Medicine, New Haven, CT, USA

[#] Correspondence: tt252@cornell.edu

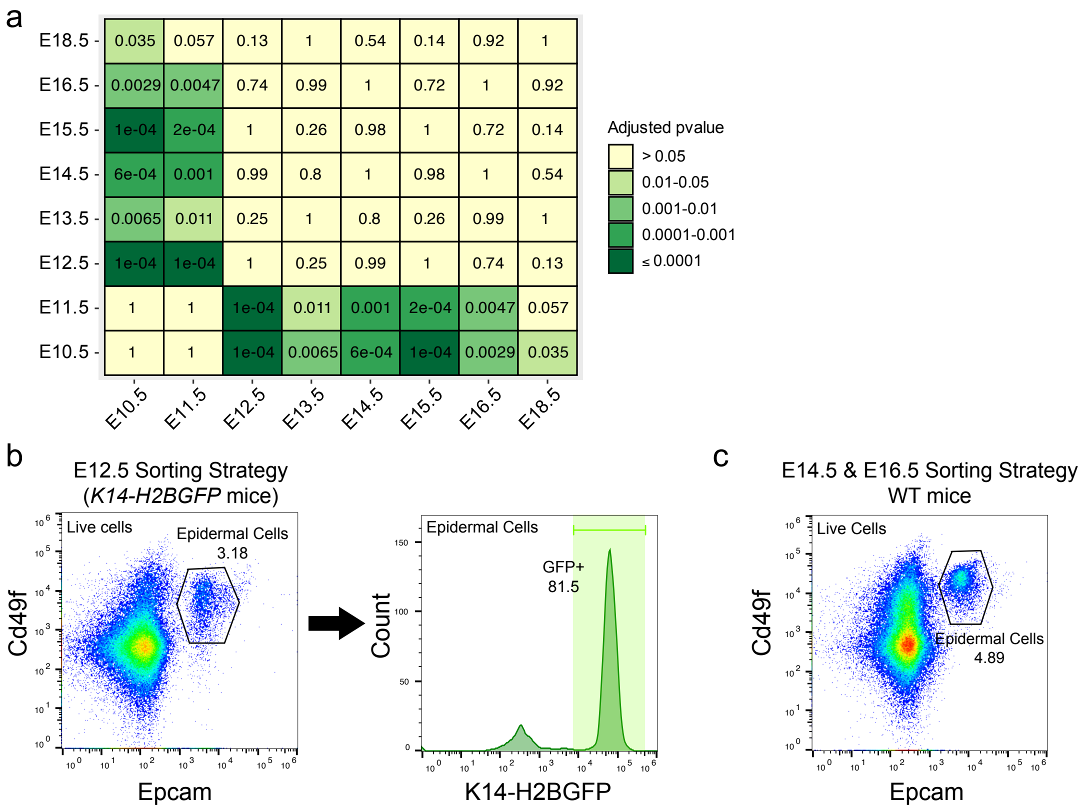

d

Stages	Peak Counts	Peak Size (bp)				
		Min	Q1	Median	Q3	Max
E12.5	53660	500	4500	6500	10000	1094000
E14.5	53909	500	4000	6500	10000	1816500
E16.5	37793	500	6000	9000	15000	1255000

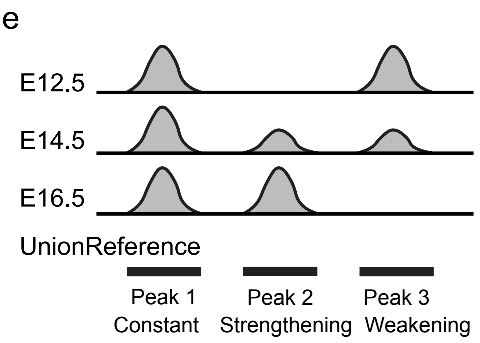

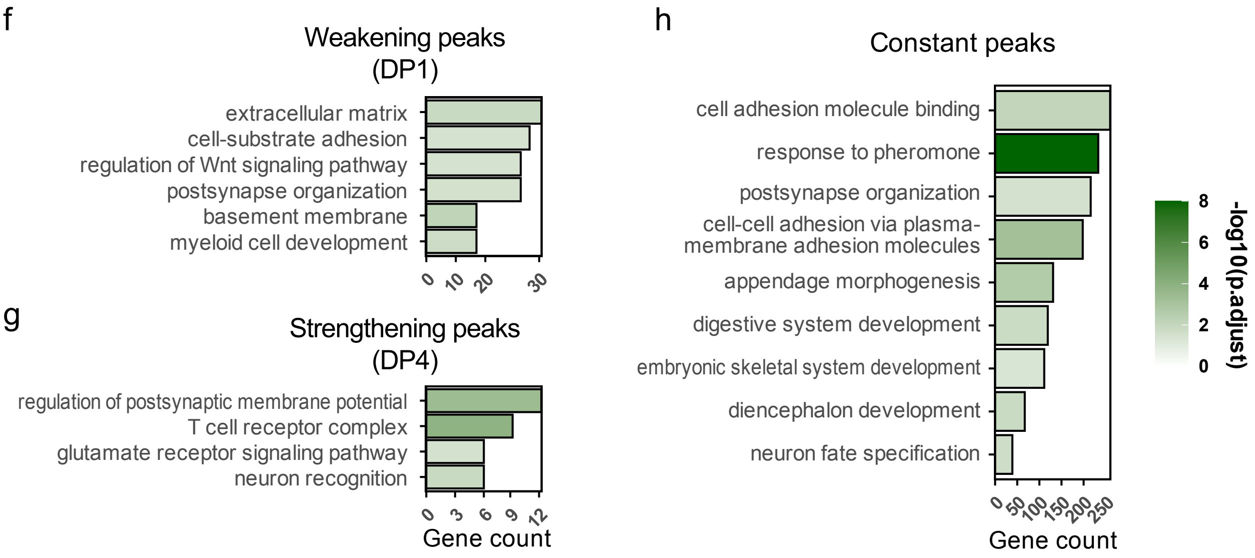

Supplementary Fig. 1 | Characterizing H3K9me3 landscape during epidermis development by IF and CUT&RUN.

a Statistical result for all pairwise comparisons between H3K9me3 IF signals from different developmental stages shown in Fig. 1c. P-values are adjusted by Tukey's method. **b** FACS strategy for isolating E12.5 epidermal cells used for CUT&RUN. **c** FACS strategy for isolating E14.5 and E16.5 epidermal cells used for CUT&RUN. Epidermal cells were gated as singlets (FSC/SSC gated), live (propidium iodide negative) cells positive for all indicated markers. **d** Five-number statistical summary of peaks called by Epic2, for E12.5, E14.5 and E16.5 samples. Q1, the first quartile; Q3, the third quartile. **e** Schematics illustrating constant, strengthening and weakening CUT&RUN peaks. Union reference is obtained by combining called peaks from all 3 time points. **f-h** Selected GO terms enriched in genes associated with dynamic peaks: DP1 (f), DP2 (g) and constant (h). Gene-peak association is defined if H3K9me3 peak(s) overlaps gene body or ±10kb flanking region of a gene. GO analysis was performed using clusterProfiler based on a one-sided hypergeometric test with Benjamini–Hochberg correction for multiple comparisons. Full lists of enriched GO terms are reported in Supplementary Data 2.

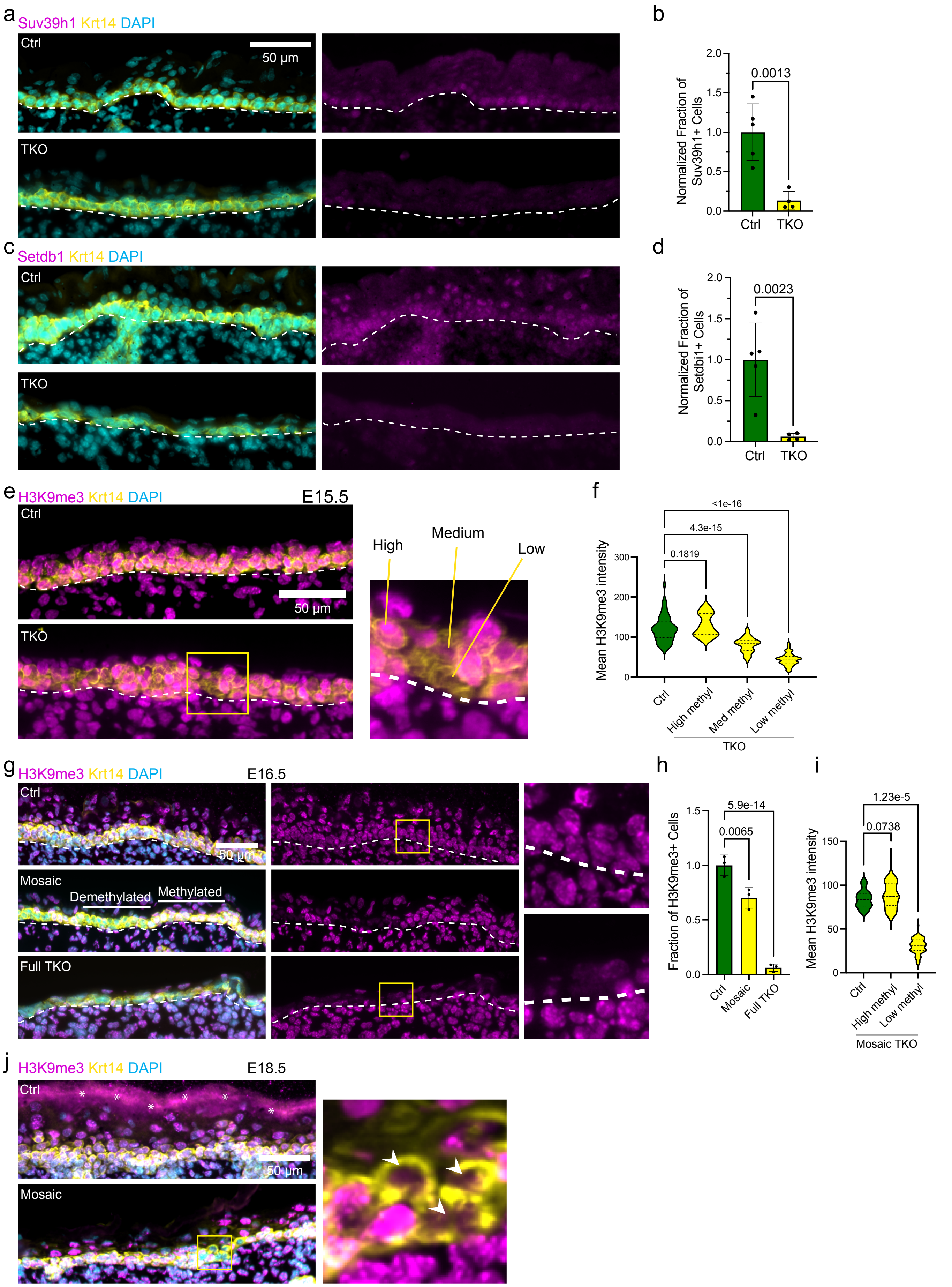

a
Suv39h1 Krt14 DAPI
Ctrl
50 µm
TKO
b
Normalized Fraction of Suv39h1+ Cells
0.0013
2.0
1.5
1.0
0.5
0.0
Ctrl
TKO
c
Setdb1 Krt14 DAPI
Ctrl
TKO
d
Normalized Fraction of Setdbi1+ Cells
0.0023
2.0
1.5
1.0
0.5
0.0
Ctrl
TKO
e
H3K9me3 Krt14 DAPI
E15.5
Ctrl
50 µm
TKO
High
Medium
Low
f
Mean H3K9me3 intensity
<1e-16
4.3e-15
0.1819
300
200
100
0
Ctrl
High methyl
Med methyl
Low methyl
TKO
g
H3K9me3 Krt14 DAPI
E16.5
Ctrl
50 µm
Mosaic
Demethylated
Methylated
Full TKO
h
Fraction of H3K9me3+ Cells
5.9e-14
0.0065
1.5
1.0
0.5
0.0
Ctrl
Mosaic
Full TKO
i
Mean H3K9me3 intensity
1.23e-5
0.0738
150
100
50
0
Ctrl
High methyl
Low methyl
Mosaic TKO
j
H3K9me3 Krt14 DAPI
E18.5
Ctrl
50 µm
Mosaic

Supplementary Fig. 2 | Induced TKO ablated HMTs and depleted H3K9me3 in mosaic patterns.

a Immunofluorescence (IF) staining showing that Suv39h1 was depleted across basal layer (Krt14+ cells) at E16.5. Note that Suv39h1+ is preferentially expressed in basal cells in Ctrl. **b** Fractions of Suv39h1+ nuclei among all DAPI+ nuclei. TM E9.5-11.5; n=5 Ctrl and n=4 TKO embryos. **c** IF staining showing successful Setdb1 depletion across basal layer at E16.5. Note that Setdb1 was ubiquitously expressed in both basal and suprabasal cells in Ctrl. **d** Fraction of Setdb1+ nuclei among all DAPI+ nuclei. TM E9.5-11.5; n=5 Ctrl and n=4 TKO embryos. **e** IF staining showing that H3K9me3 depletion is incomplete in basal cells at E15.5. Insets highlight H3K9me3-high, -medium and -low cells. **f** Quantificartion for nuclear intensity of H3K9me3 signal. TM E10.5-12.5; N=62, 38, 47, 74 cells for the four groups, respectively. **g** IF staining of E16.5 embryos showing that H3K9me3 was globally depleted in all basal cells (Full TKO) or in a subset of cells (Mosaic). Insets highlight H3K9me3 depletion in basal cells. **h** Fractions of H3K9me3+ nuclei among all DAPI+ basal nuclei. N=3 biologically independent embryos per group; TM from E9.5-11.5 for mosaic samples; TM from E10.5-12.5 for full TKO samples. **i** Quantification of nuclear H3K9me3 intensity showing ‘all-or-none’ pattern of H3K9me3 depletion in mosaic samples. N=60, 60, 55 cells for the three groups, respectively. See Fig. 2e for quantification of epidermis thickness. **j** IF staining of E18.5 mosaic embryos showing decreased epidermal thickness and lack of cornified envelope. Cornified envelope is visible as auto-fluorescent regions in magenta channel (asterisks). Inset highlights a mosaic region. Arrow heads indicate H3K9me3-depleted cells. TM E10.5-12.5. n=3 Ctrl and n=5 Mosaic embryos. See Fig. 2f for quantifications of epidermis thickness. Dashed lines mark the basement membrane. All images are representative. Unpaired t-test was performed for b, d, and two-tailed p values are reported. One-way ANOVA with multiple comparisons was performed for f, h, i, and p values were corrected for multiple comparisons using the Dunnett method. Data are presented as mean ± s.d.

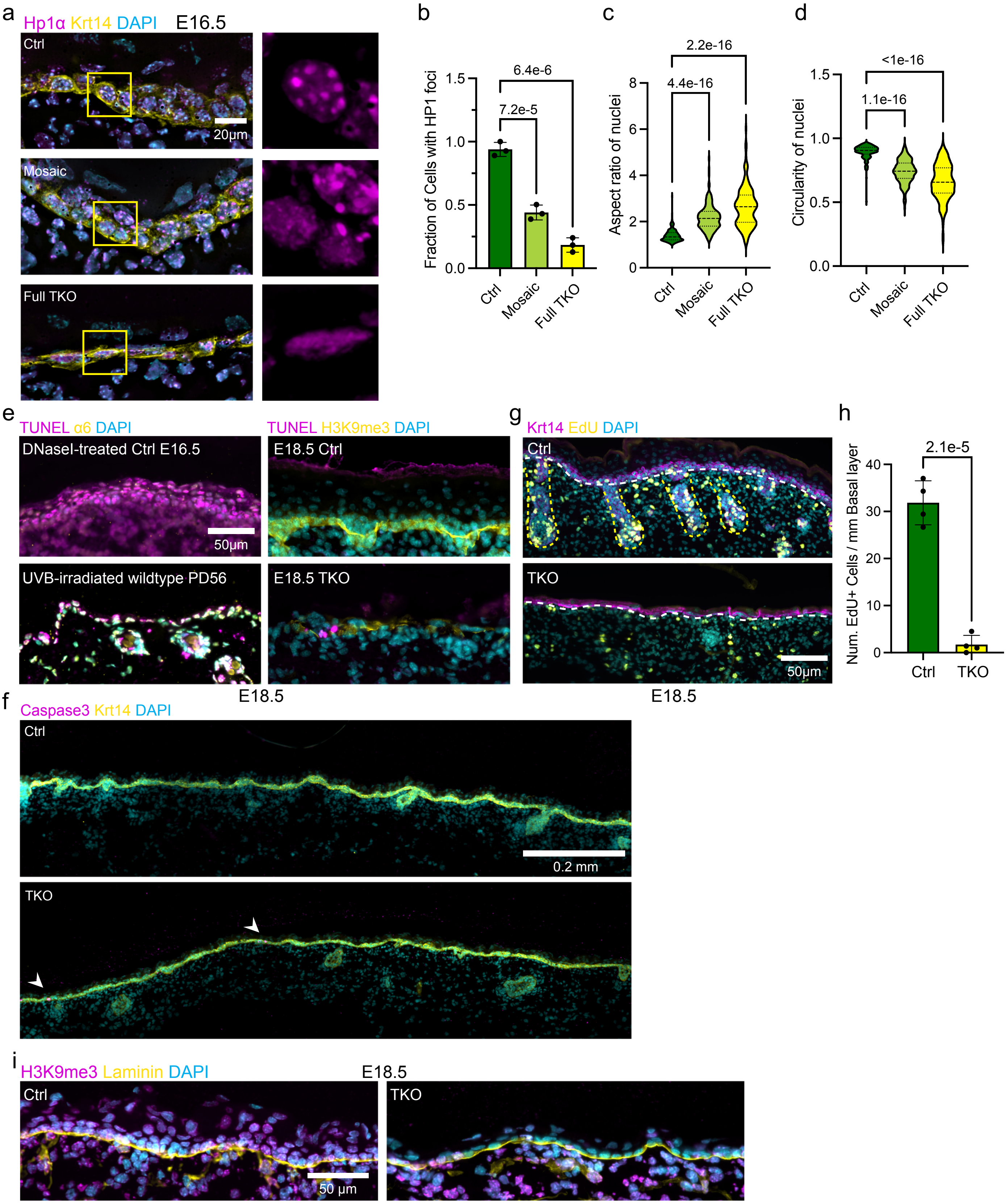

a
Hp1α Krt14 DAPI
E16.5
Ctrl
20µm
Mosaic
Full TKO
b
Fraction of Cells with HP1 foci
6.4e-6
7.2e-5
1.5
1.0
0.5
0.0
Ctrl
Mosaic
Full TKO
c
Aspect ratio of nuclei
2.2e-16
4.4e-16
8
6
4
2
0
Ctrl
Mosaic
Full TKO
d
Circularity of nuclei
<1e-16
1.1e-16
1.5
1.0
0.5
0.0
Ctrl
Mosaic
Full TKO
e
TUNEL α6 DAPI
DNaseI-treated Ctrl E16.5
50µm
UVB-irradiated wildtype PD56
TUNEL H3K9me3 DAPI
E18.5 Ctrl
E18.5 TKO
E18.5
g
Krt14 EdU DAPI
Ctrl
TKO
50µm
E18.5
h
Num. EdU+ Cells / mm Basal layer
2.1e-5
40
30
20
10
0
Ctrl
TKO
f
Caspase3 Krt14 DAPI
Ctrl
0.2 mm
TKO
i
H3K9me3 Laminin DAPI
E18.5
Ctrl
50 µm
TKO

Supplementary Fig. 3 | H3K9me3 loss disrupted heterochromatin and arrested cell cycle with minimal DNA damage and cell death.

a IF staining for Hp1a (magenta) illustrating loss of Hp1a foci in E16.5 TKO samples. Images were captured as a series of 63x z-stacks and deconvolution was then performed to eliminate the out of focus blur. **b-d** Quantification of 20x images stained for Hp1a showing: **b** Hp1a+ cell fraction decreased in TKO. N=3 embryos per group; **c, d** nuclear shapes are flatter and more elongated in TKO, TM E9.5-11.5, Ctrl, n = 180 cells; Mosaic, n=150 cells; Full TKO, n=150 cells. Aspect ratio and circularity values were obtained with Fiji. **e** TUNEL assay on E18.5 Ctrl and TKO samples and positive control samples. See Fig. 3b for quantifications. **f** IF staining for active Caspase3 (magenta) across E16.5 back skin showing the sparsity of apoptotic cells. A series of 10x images were stitched together to show longer stretches. See Fig. 3d for quantifications. **g-h** IF staining for EdU (yellow) and quantification showing proliferation arrest in E18.5 TKO epidermis. White dashed lines mark the basement membrane. Yellow dashed lines outline hair follicles. TM E12.5-14.5; n=4 embryos per group. **i** IF staining for laminin (yellow) showing that basal membrane integrity is well maintained in E16.5 epiermis despite full depletion of H3K9me3. TM E9.5-11.5; n=3 embryos per group. See main Fig. 3k for images at E18.5. All images are representative. Unpaired t-test was performed for h, and the two-tailed p value is reported. One-way ANOVA with multiple comparison was performed for b-d, and p values were corrected for multiple comparisions using the Dunnett method. Data are presented as mean ± s.d.

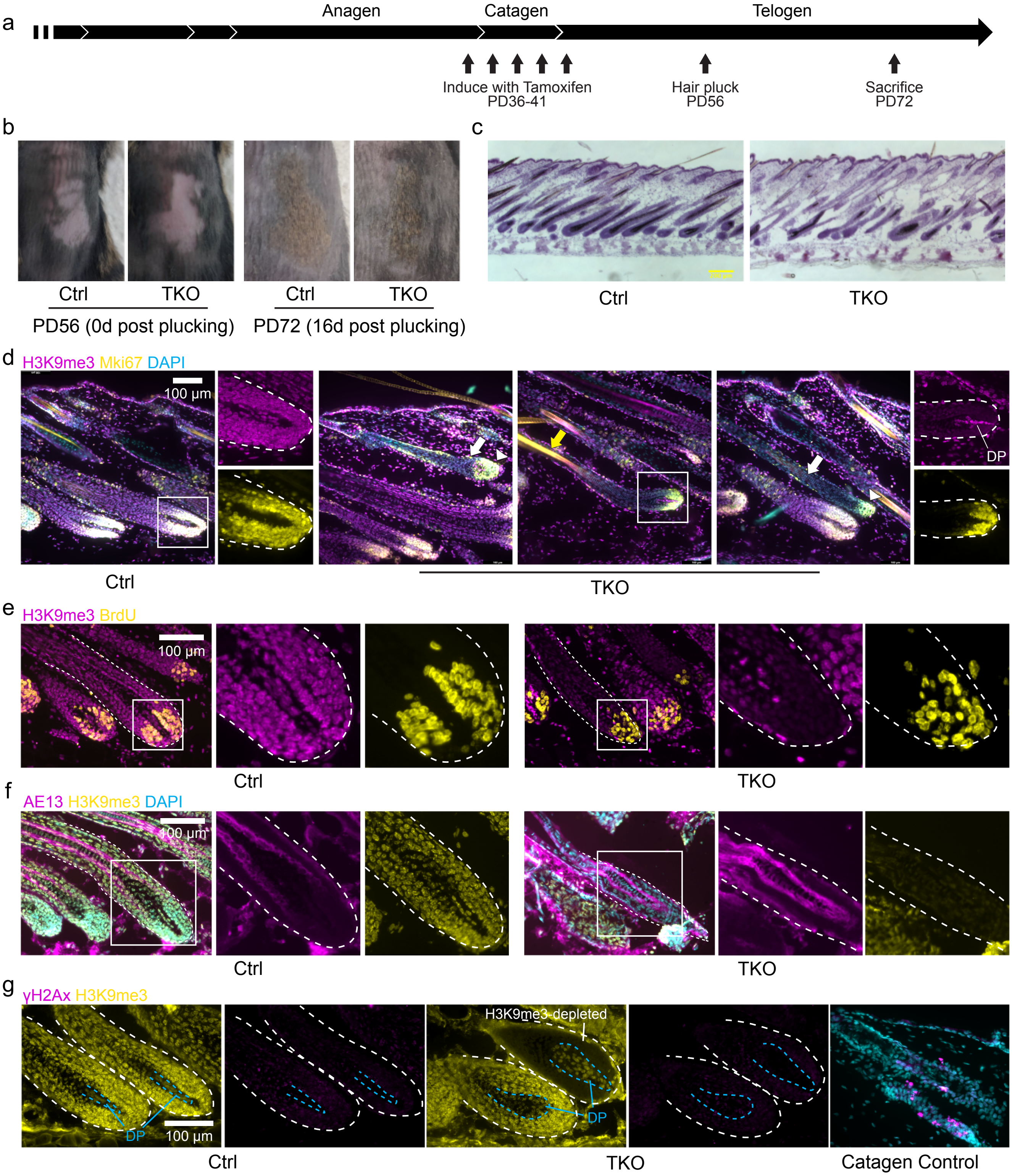
a
Anagen
Catagen
Telogen
Induce with Tamoxifen
PD36-41
Hair pluck
PD56
Sacrifice
PD72
b
Ctrl
TKO
Ctrl
TKO
PD56 (0d post plucking)
PD72 (16d post plucking)
c
Ctrl
TKO
d
H3K9me3 Mki67 DAPI
100 µm
DP
Ctrl
TKO
e
H3K9me3 BrdU
100 µm
Ctrl
TKO
f
AE13 H3K9me3 DAPI
100 µm
Ctrl
TKO
g
γH2Ax H3K9me3
H3K9me3-depleted
DP
100 µm
DP
Ctrl
TKO
Catagen Control

Supplementary Fig. 4 | H3K9me3 depletion in adult skin led to skewed hair follicle regeneration

a Experimental scheme showing the timeline of tamoxifen induction, hair plucking, and sample collection. **b** Pictures showing grossly normal hair regeneration after plucking. **c** H&E staining showing hair follicles with mildly irregular appearance in TKO. **d** IF staining for H3K9me3 (magenta) showing H3K9me3-depleted hair follicles (white arrows) are smaller and thinner, with Ki67+ proliferating cells strongly represented in the matrix (arrowhead). Yellow arrow points a hair shaft produced by H3K9me3-depleted hair follicles. Insets show single-channel representations of H3K9me3-depleted matrix. **e** IF staining showing BrdU+ cells in matrix progenitor cells, including in H3K9me3-depleted hair follicles. Matrix regions in white boxes are enlarged, and single-channel images were shown. **f** IF staining of AE13 (magenta) and H3K9me3 (yellow) showing the presence of hair shaft lineage when H3K9me3 is lost. Matrix regions in white boxes are enlarged, and single-channel images are shown. **g** IF staining of γH2Ax (magenta) and H3K9me3 (yellow) showing that H3K9me3 loss did not increase DNA damage in hair follicle matrix (white dashed lines). Dermal papillae (DP) are marked by cyan dashed lines, which are not targeted by $K14CreER^{T2}$ for TKO. Catagen follicles serve as a positive control for γH2Ax staining. N=4 pairs of Ctrl-TKO littermates from four different litters that were independently induced, plucked, collected and assayed and representative images are shown.

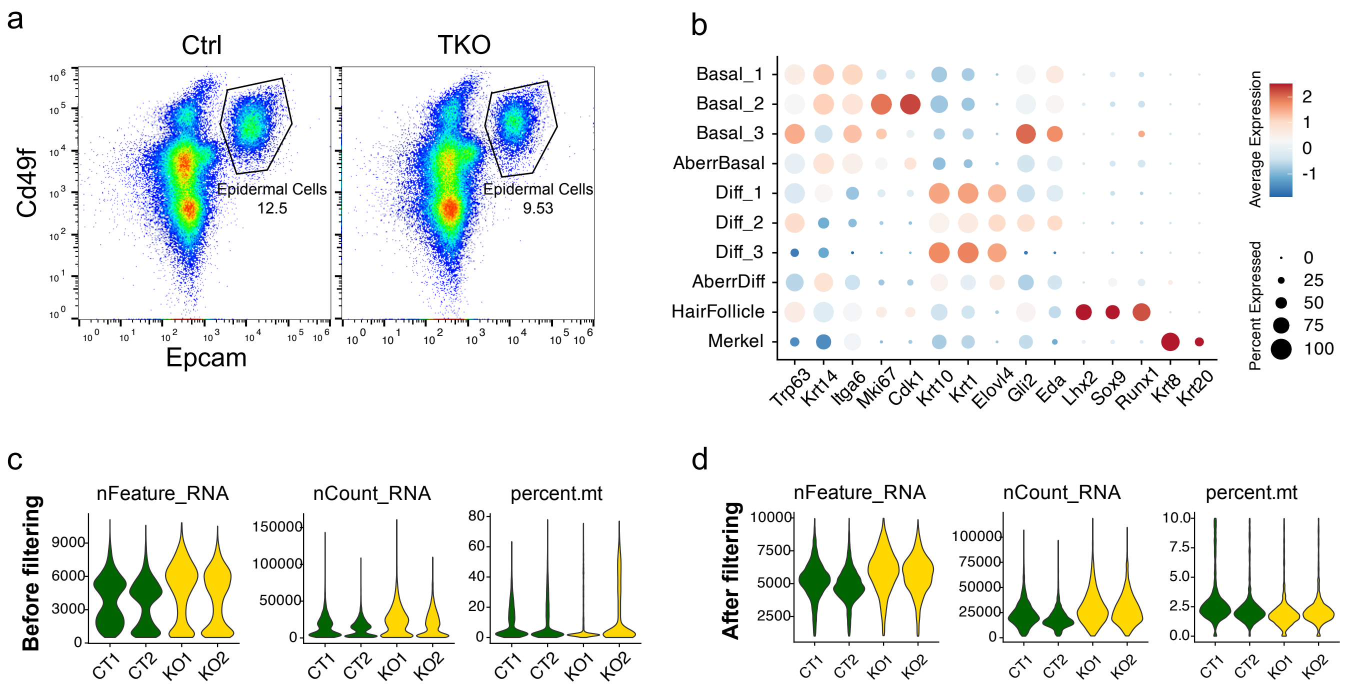
a
Ctrl
TKO
Cd49f
Epcam
Epidermal Cells
12.5
Epidermal Cells
9.53
b
Basal_1
Basal_2
Basal_3
AberrBasal
Diff_1
Diff_2
Diff_3
AberrDiff
HairFollicle
Merkel
Trp63
Krt14
Itga6
Mki67
Cdk1
Krt10
Krt1
Elovl4
Gli2
Eda
Lhx2
Sox9
Runx1
Krt8
Krt20
Average Expression
Percent Expressed
c
Before filtering
nFeature_RNA
nCount_RNA
percent.mt
CT1
CT2
KO1
KO2
d
After filtering
nFeature_RNA
nCount_RNA
percent.mt

Supplementary Fig. 5 | Quality control and pre-filtering of scRNA-seq data

a FACS scheme for isolating epidermal cells (Epcam+/Cd49f+) at E16.5 for single cell RNA sequencing. **b** Dot plot showing the marker genes used for assigning cell identity to keratinocyte clusters. **c, d** The number of total features (nFeature_RNA), total read counts (nCount_RNA) and percentage of mitochondrial features (percent.mt) for each sample before (c) and after (d) low quality cell filtering.

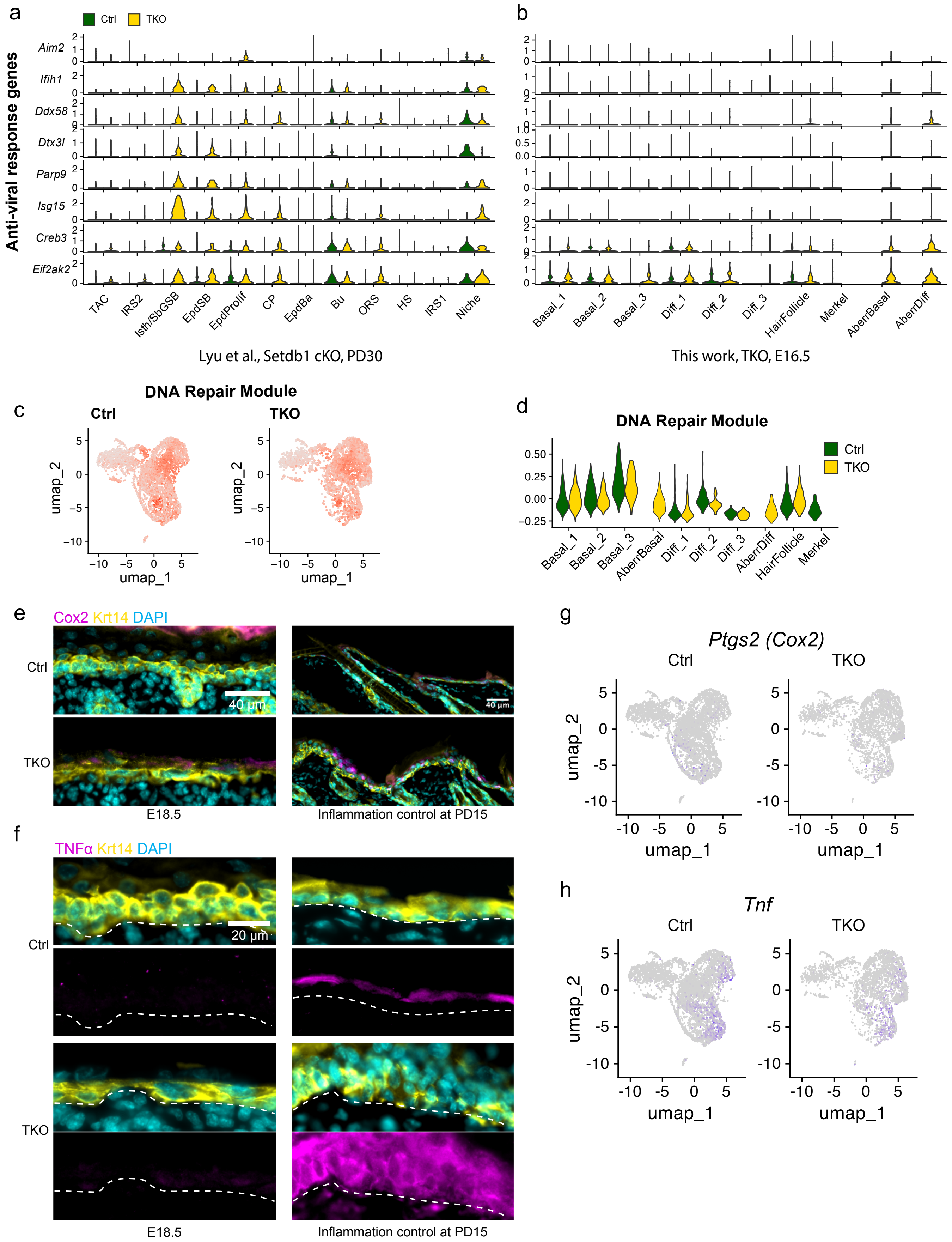

a
Ctrl
TKO
Anti-viral response genes
Aim2
Ifih1
Ddx58
Dtx3l
Parp9
Isg15
Creb3
Eif2ak2
TAC
IRS2
Isth/SbGSB
EpdSB
EpdProlif
CP
EpdBa
Bu
ORS
HS
IRS1
Niche
Lyu et al., Setdb1 cKO, PD30
b
Basal_1
Basal_2
Basal_3
Diff_1
Diff_2
Diff_3
HairFollicle
Merkel
AberrBasal
AberrDiff
This work, TKO, E16.5
c
DNA Repair Module
Ctrl
TKO
umap_2
umap_1
d
DNA Repair Module
Ctrl
TKO
AberrBasal
AberrDiff
HairFollicle
Merkel
e
Cox2 Krt14 DAPI
Ctrl
TKO
40 μm
E18.5
Inflammation control at PD15
f
TNFα Krt14 DAPI
Ctrl
TKO
20 μm
E18.5
Inflammation control at PD15
g
Ptgs2 (Cox2)
Ctrl
TKO
umap_2
umap_1
h
Tnf
Ctrl
TKO
umap_2
umap_1

Supplementary Fig. 6 | H3K9me3 depletion induced anti-viral response in postnatal but not embryonic skin.

a, b Violin plots showing that anti-viral response genes were widely activated in postnatal day 30 (PD30) Setdb1 single knockout (Lyu et al., GSE233240)[1], but very limited in the TKO E16.5 embryos. **c, d** Feature plot and violin plot showing that DNA repair module is not significantly different between Ctrl and TKO shared populations. Aberrant populations show expressions comparable with Ctrl basal cells. Genes for defining DNA repair module are obtained from GO database using keyword “DNA damage response”. **e, f** IF staining showing that inflammation markers, TNFα and Cox2, are not active in embryonic skin. Images were representative; n=3 embryos per group. Positive inflammation control samples are from skin of surviving escaper mosaic TKO pups induced by K14-Cre, collected at postnatal day 15 (PD15). **g, h** Feature plot from scRNA-seq data at E16.5 showing that Tnf and Ptgs2 (Cox2) are not significantly upregulated in TKO compared to Ctrl.

a

(Dai and Segre, 2004; Liu et al., 2013)

Gene	Function
Trp63	Master regulator of epidermal maintenance and stratification.
Klf4	Required for proper terminal differentiation to form cornified envelope
Irf6	Regulate proliferation-differentiation switch of basal cells
Ovol1	Required for proliferation exit of epidermal progenitor cells
Id1	Maintaining basal cell identity and inhibit precocious differentiation
Foxn1	Stimulates early differentiation but suppresses terminal differentiation
Myc	Regulate epidermal proliferation; Transgenic activation stimulates differentiation to epidermal and sebaceous lineages
Gli2	Controls epidermal cell proliferation downstream of Shh signaling; Important for hair follicle develop-ment
Chuk	Also known as Ikka; Loss of function in developing epidermis leads to hyper-proliferation and dysregulated differentiation

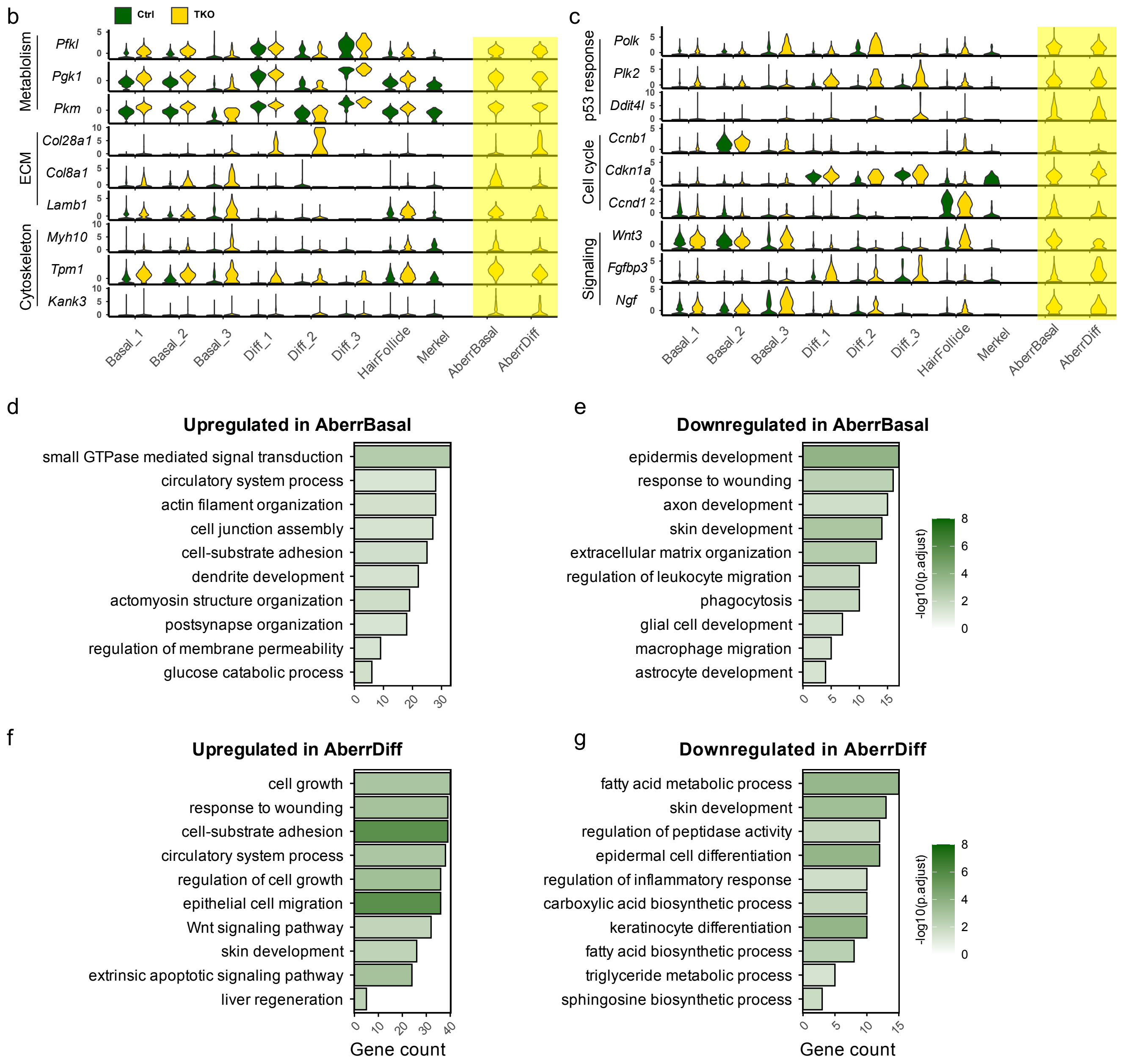

Supplementary Fig. 7 | TKO dysregulated genes from scRNAseq data are important for cell fate control.

a Genes dysregulated in TKO scRNA-seq clusters that are known from previous functional studies to be important for epidermal development[2,3]. **b, c** Violin plots showing the dysregulation of genes relating to processes important for epidermal cell function. Yellow regions highlight gene expression from aberrant populations. **d-g** Selected GO terms enriched in genes dysregulated in AberrBasal and AberrDiff. GO analysis was performed using clusterProfiler based on a one-sided hypergeometric test with Benjamini–Hochberg correction for multiple comparisons. See Fig. 4f for SimplifyEnrichment analysis that clusters similar GO terms. Full lists of enriched GO terms are reported in Supplementary Data 2.

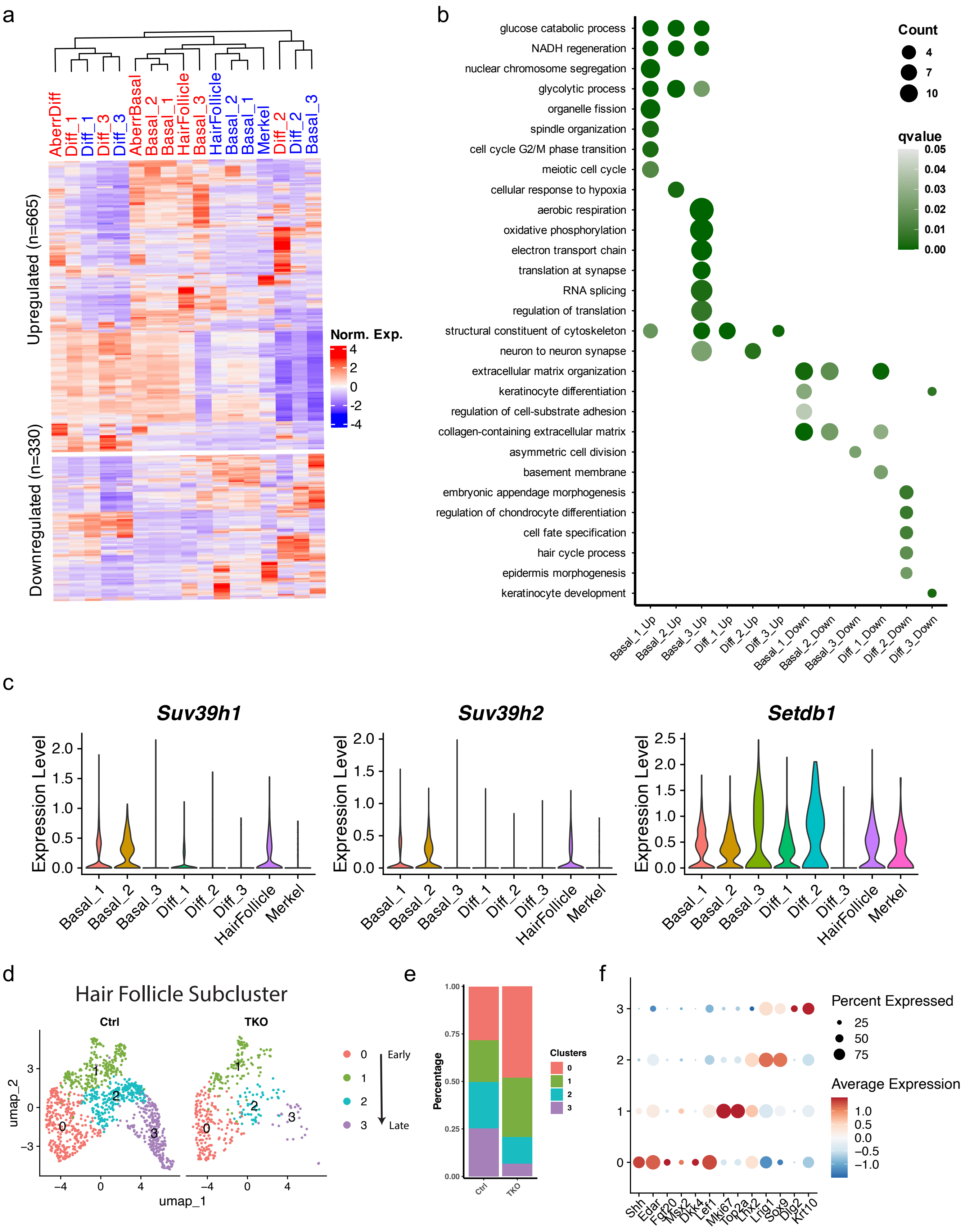

a
Upregulated (n=665)
Downregulated (n=330)
Norm. Exp.
AberrDiff
Diff_1
Diff_1
Diff_3
Diff_3
AberrBasal
Basal_2
Basal_1
HairFollicle
Basal_3
HairFollicle
Basal_2
Basal_1
Merkel
Diff_2
Diff_2
Basal_3
b
glucose catabolic process
NADH regeneration
nuclear chromosome segregation
glycolytic process
organelle fission
spindle organization
cell cycle G2/M phase transition
meiotic cell cycle
cellular response to hypoxia
aerobic respiration
oxidative phosphorylation
electron transport chain
translation at synapse
RNA splicing
regulation of translation
structural constituent of cytoskeleton
neuron to neuron synapse
extracellular matrix organization
keratinocyte differentiation
regulation of cell-substrate adhesion
collagen-containing extracellular matrix
asymmetric cell division
basement membrane
embryonic appendage morphogenesis
regulation of chondrocyte differentiation
cell fate specification
hair cycle process
epidermis morphogenesis
keratinocyte development
Basal_1_Up
Basal_2_Up
Basal_3_Up
Diff_1_Up
Diff_2_Up
Diff_3_Up
Basal_1_Down
Basal_2_Down
Basal_3_Down
Diff_1_Down
Diff_2_Down
Diff_3_Down
Count
qvalue
c
Suv39h1
Suv39h2
Setdb1
Expression Level
Basal_1
Basal_2
Basal_3
Diff_1
Diff_2
Diff_3
HairFollicle
Merkel
d
Hair Follicle Subcluster
Ctrl
TKO
umap_1
umap_2
Early
Late
e
Percentage
Clusters
Ctrl
TKO
f
Percent Expressed
Average Expression
Shh
Edar
Fgf20
Msx2
Dkk4
Lef1
Mki67
Top2a
Lhx2
Lrig1
Sox9
Dlg2
Krt10

Supplementary Fig. 8 | Transcriptome of keratinocyte lineages in TKO are dysregulated from scRNA-seq data.

a Heatmap showing differentially expressed genes in TKO vs Ctrl cell clusters. Top labeling denotes clusters from TKO in red and Ctrl in blue. **b** Dot plot showing selected subset of GO terms enriched in TKO dysregulated genes shown in a. GO analysis was performed using clusterProfiler based on a one-sided hypergeometric test with Benjamini–Hochberg correction for multiple comparisons. Full lists of enriched GO terms are reported in Supplementary Data 2. **c** Violin plots showing the expression of H3K9me3 histone methyltransferases in Ctrl samples. **d, e** Subcluster analyses of hair follicle population in Ctrl and TKO showing that hair follicle lineage cells were skewed towards early-stage morphogenesis (cluster 0 and 1). **f** Dot plot showing marker genes for the four subclusters of hair follicle populations shown in panel d.

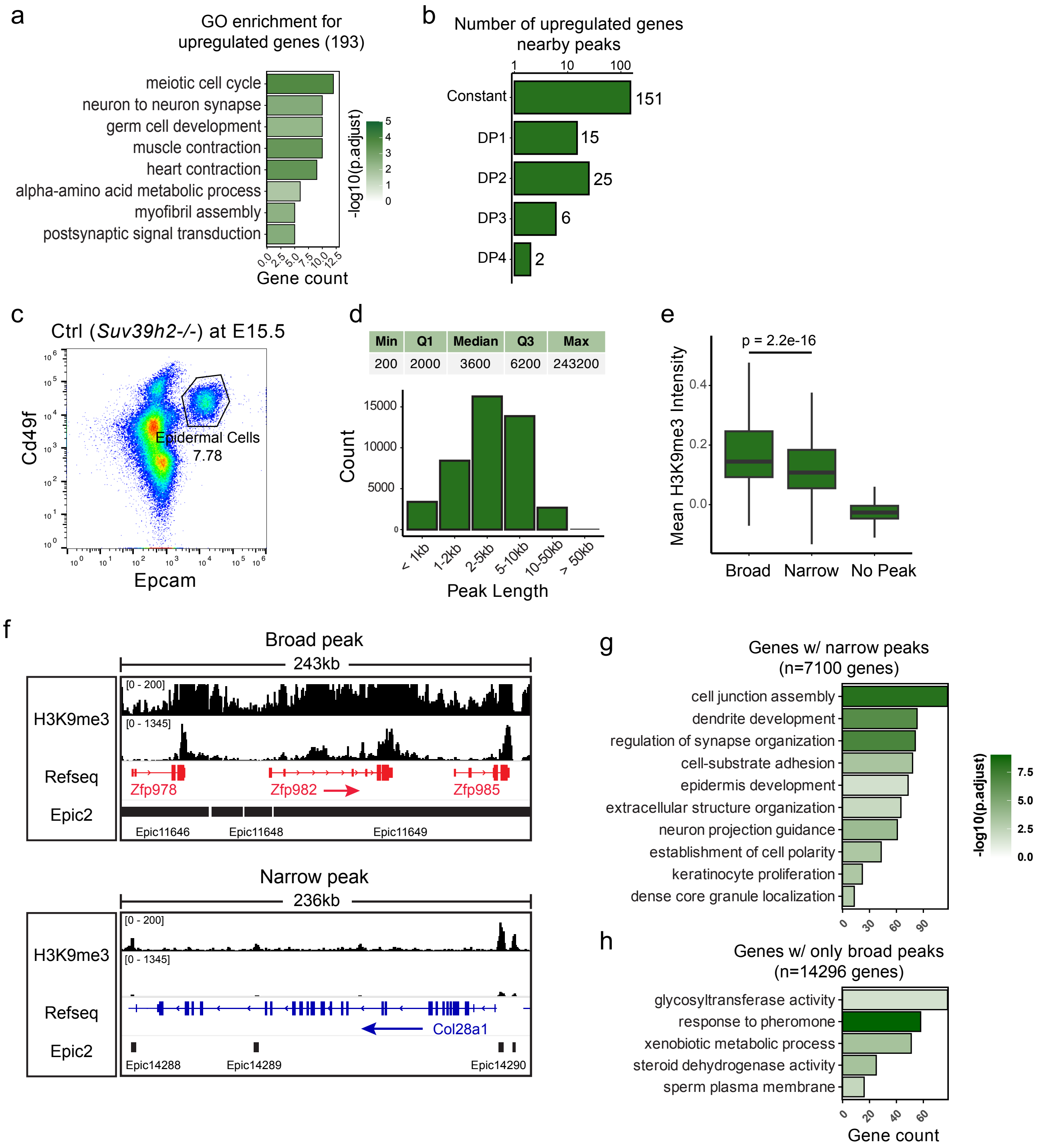
a
GO enrichment for upregulated genes (193)
meiotic cell cycle
neuron to neuron synapse
germ cell development
muscle contraction
heart contraction
alpha-amino acid metabolic process
myofibril assembly
postsynaptic signal transduction
-log10(p.adjust)
Gene count
b
Number of upregulated genes nearby peaks
Constant 151
DP1 15
DP2 25
DP3 6
DP4 2
c
Ctrl (Suv39h2-/-) at E15.5
Cd49f
Epcam
Epidermal Cells 7.78
d
Min Q1 Median Q3 Max
200 2000 3600 6200 243200
Count
Peak Length
< 1kb
1-2kb
2-5kb
5-10kb
10-50kb
> 50kb
e
p = 2.2e-16
Mean H3K9me3 Intensity
Broad
Narrow
No Peak
f
Broad peak
243kb
H3K9me3
Refseq
Epic2
[0 - 200]
[0 - 1345]
Zfp978
Zfp982
Zfp985
Epic11646
Epic11648
Epic11649
Narrow peak
236kb
Col28a1
Epic14288
Epic14289
Epic14290
g
Genes w/ narrow peaks (n=7100 genes)
cell junction assembly
dendrite development
regulation of synapse organization
cell-substrate adhesion
epidermis development
extracellular structure organization
neuron projection guidance
establishment of cell polarity
keratinocyte proliferation
dense core granule localization
-log10(p.adjust)
h
Genes w/ only broad peaks (n=14296 genes)
glycosyltransferase activity
response to pheromone
xenobiotic metabolic process
steroid dehydrogenase activity
sperm plasma membrane
Gene count

Supplementary Fig. 9 | Bulk RNA-seq and genome-wide profiling of H3K9me3 landscapes by CUT&RUN

a Selected subset of GO terms enriched in upregulated genes from bulk RNA-seq at E16.5, reflecting the function of TKO-dysregulated mRNAs in the epidermal lineage. **b** Barplot showing the number of TKO-upregulated genes (bulk RNA-seq) associated with each H3K9me3 dynamic peak type during development. **c** FACS strategy for isolating E15.5 epidermal cells (Epcam+ Cd49f+) from Ctrl embryos. Note that CUT&RUN at the E15.5 sample time point in Ctrl more accurately defines the naïve H3K9me3 landscape prior to phenotype characterization at E16.5 (used for bulk RNA-seq). **d** Size distribution for Epic2 peaks called from E15.5 Ctrl samples. Q1, the first quartile; Q3, the third quartile. **e** Box plot showing that broad CUT&RUN peaks have stronger signal than narrow peaks. Narrow peaks are defined as peaks smaller than 2000bp, which is the first quartile number in d. n=11781 narrow peaks and n=32738 broad peaks. 'No Peak' represents randomly shuffled regions not overlapping any called Epic2 peaks, n=44519 regions. Box plots show the median (center line), interquartile range (box; 25th–75th percentiles), and whiskers extending to the minimum and maximum values no further than 1.5× the interquartile range. Statistical significance was assessed using two-sided unpaired t-tests. **f** IGV browser tracks showing examples of H3K9me3 in forms of large domains (top) or small islands (bottom). H3K9me3 tracks with two scales (0-200 and 0-1345) are used to accommodate the large signal intensity differences from broad and narrow peaks while illustrating the pattern. **g, h** Selected subset of GO terms enriched in genes associated with narrow peaks (g) or with only broad peaks (h) from E15.5 Ctrl CUT&RUN, highlighting that narrow peaks are more commonly associated with developmental and lineage-related genes. GO analysis was performed using clusterProfiler based on a one-sided hypergeometric test with Benjamini–Hochberg correction for multiple comparisons.

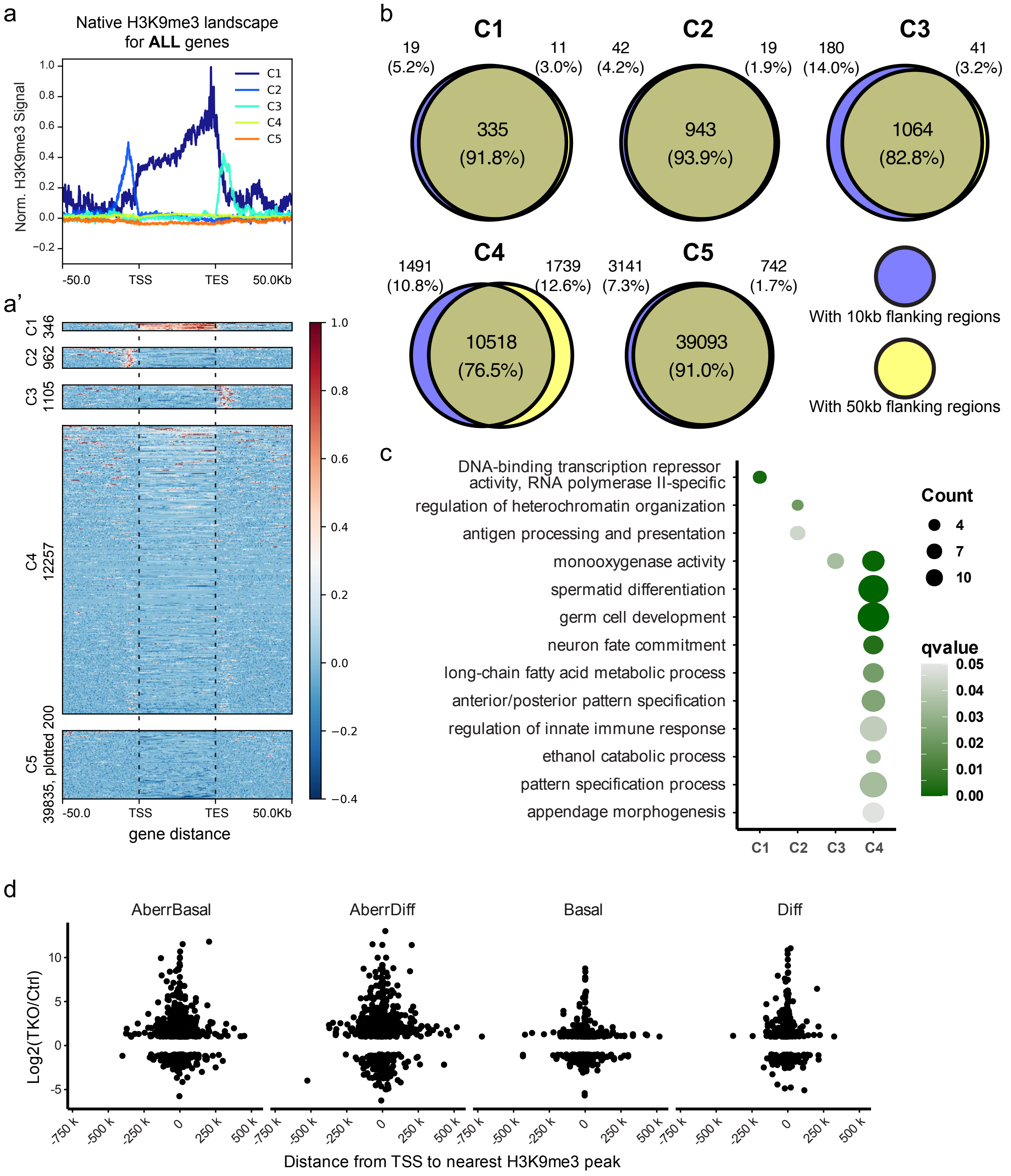
a
Native H3K9me3 landscape
for ALL genes
Norm. H3K9me3 Signal
C1
C2
C3
C4
C5
-50.0
TSS
TES
50.0Kb
a'
C1 346
C2 962
C3 1105
C4 12257
C5 39835, plotted 200
gene distance
b
C1
19 (5.2%)
335 (91.8%)
11 (3.0%)
C2
42 (4.2%)
943 (93.9%)
19 (1.9%)
C3
180 (14.0%)
1064 (82.8%)
41 (3.2%)
C4
1491 (10.8%)
10518 (76.5%)
1739 (12.6%)
C5
3141 (7.3%)
39093 (91.0%)
742 (1.7%)
With 10kb flanking regions
With 50kb flanking regions
c
DNA-binding transcription repressor activity, RNA polymerase II-specific
regulation of heterochromatin organization
antigen processing and presentation
monooxygenase activity
spermatid differentiation
germ cell development
neuron fate commitment
long-chain fatty acid metabolic process
anterior/posterior pattern specification
regulation of innate immune response
ethanol catabolic process
pattern specification process
appendage morphogenesis
Count
4
7
10
qvalue
0.05
0.04
0.03
0.02
0.01
0.00
d
AberrBasal
AberrDiff
Basal
Diff
Log2(TKO/Ctrl)
Distance from TSS to nearest H3K9me3 peak

Supplementary Fig. 10 | H3K9me3 associates with and regulates lineage genes.

a, a' Metaplot (a) and heatmap (a') showing naïve H3K9me3 landscape from Ctrl E15.5 epidermis at gene loci with ±50kb flanking regions. Gene body regions are scaled to 50kb for visualization. Numbers immediately left of panels in a' represent total number of genes in each cluster. 1000 genes are randomly selected from C1-C4 and 200 genes selected from C5 for visualization. **b** Venn diagram showing H3K9me3-assciated gene clusters obtained from analysis using 50±kb flanking regions compared with similar analysis using ±10kb flanking regions. Note the large overlap between the two groups of genes. **c** Dot plot showing selected GO terms enriched in genes from each cluster in main Fig. 5b-b', highlighting development and lineage-related terms. GO analysis was performed using clusterProfiler based on a one-sided hypergeometric test with Benjamini–Hochberg correction for multiple comparisons. Full lists of enriched GO terms are reported in Supplementary Data 2. **d** Dot plots showing the relationship between gene expression fold change (log2 transformed, TKO vs Ctrl, scRNA-seq data) and TSS-H3K9me3-peak distance. Note that TSS of upregulated genes tend to cluster nearer to H3K9me3 peaks. Basal refers to Basal_1 to Basal_3 clusters and Diff refers to Diff_1 to Diff_3 cluster (main Fig. 4a)

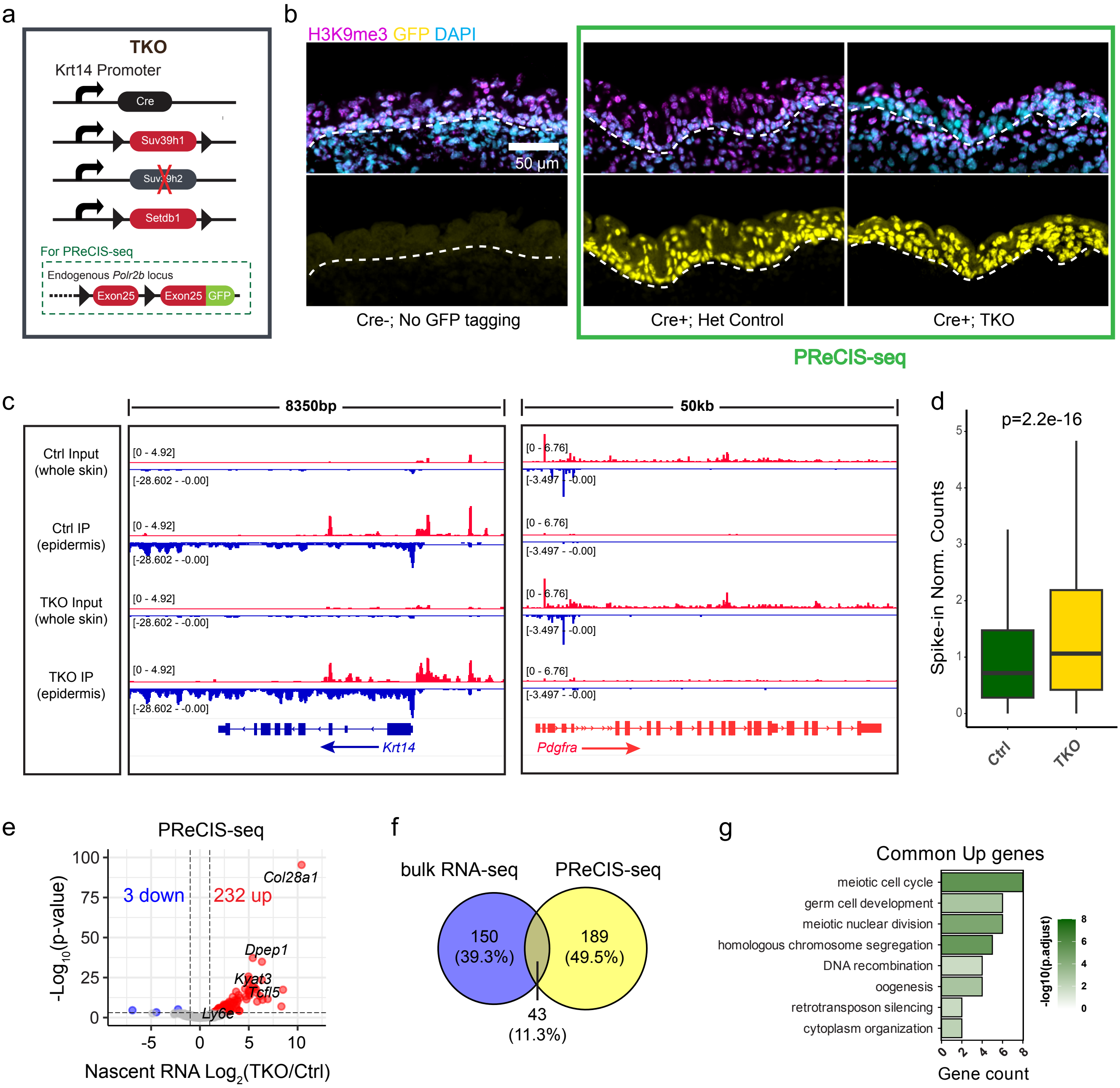

a
TKO
Krt14 Promoter
Cre
Suv39h1
Setdb1
For PReCIS-seq
Endogenous Polr2b locus
Exon25
Exon25
GFP
b
H3K9me3 GFP DAPI
50 μm
Cre-; No GFP tagging
Cre+; Het Control
Cre+; TKO
PReCIS-seq
c
8350bp
50kb
Ctrl Input (whole skin)
Ctrl IP (epidermis)
TKO Input (whole skin)
TKO IP (epidermis)
[0 - 4.92]
[-28.602 - -0.00]
[0 - 6.76]
[-3.497 - -0.00]
Krt14
Pdgfra
d
p=2.2e-16
Spike-in Norm. Counts
Ctrl
TKO
e
PReCIS-seq
3 down
232 up
Col28a1
Dpep1
Kyat3
Tcfl5
Ly6e
-Log10(p-value)
Nascent RNA Log2(TKO/Ctrl)
f
bulk RNA-seq
PReCIS-seq
150 (39.3%)
189 (49.5%)
43 (11.3%)
g
Common Up genes
meiotic cell cycle
germ cell development
meiotic nuclear division
homologous chromosome segregation
DNA recombination
oogenesis
retrotransposon silencing
cytoplasm organization
-log10(p.adjust)
Gene count

Supplementary Fig. 11 | PReCIS-seq profiles transcriptionally engaged RNA Pol II activity in mouse epidermis via inducible GFP-tagging

a Schematics showing the construct of *K14Cre; TKO; Rpol2$^{f/f}$-GFP* line for PReCIS-seq. Other elements, including exons, stop codons and polyadenylation signals, are omitted for simplicity. See Chovatiya et al.[4] for more details. **b** IF staining confirming that epidermal cells were specifically labeled with GFP (yellow) and that H3K9me3 (magenta) was reduced in TKO but not in heterozygous control skin. Images are representative. All embryos used for PReCIS-seq were individually validated for GFP and H3K9me3. n=6 embryos per group. **c** IGV tracks of *Krt14* (epithelial) and *Pdgfra* (mesenchymal) loci showing that PReCIS-seq captures engaged RNA Pol II activity specifically from epidermal cells. **d** Spike-in normalized read counts showing that TKO had globally increased nascent transcription. Counts for n=13201 genes are analyzed. Box plots show the median (center line), interquartile range (box; 25th–75th percentiles), and whiskers extending to the minimum and maximum values no further than 1.5× the interquartile range. Statistical significance was assessed using two-sided unpaired t-tests. **e** Volcano plot showing differentially expressed (DE) genes from E16.5 PReCIS-seq data. Total reads mapped to full transcripts are analyzed. DE analysis was performed using DESeq2 based on a two-sided Wald test, with P values adjusted for multiple comparisons using the Benjamini–Hochberg method. **f** Venn diagram showing limited overlap in upregulated genes obtained from bulk RNA-seq data and PReCIS-seq data. **g** Select GO terms enriched in common upregulated genes from f. GO analysis was performed using clusterProfiler based on a one-sided hypergeometric test with Benjamini–Hochberg correction for multiple comparisons.

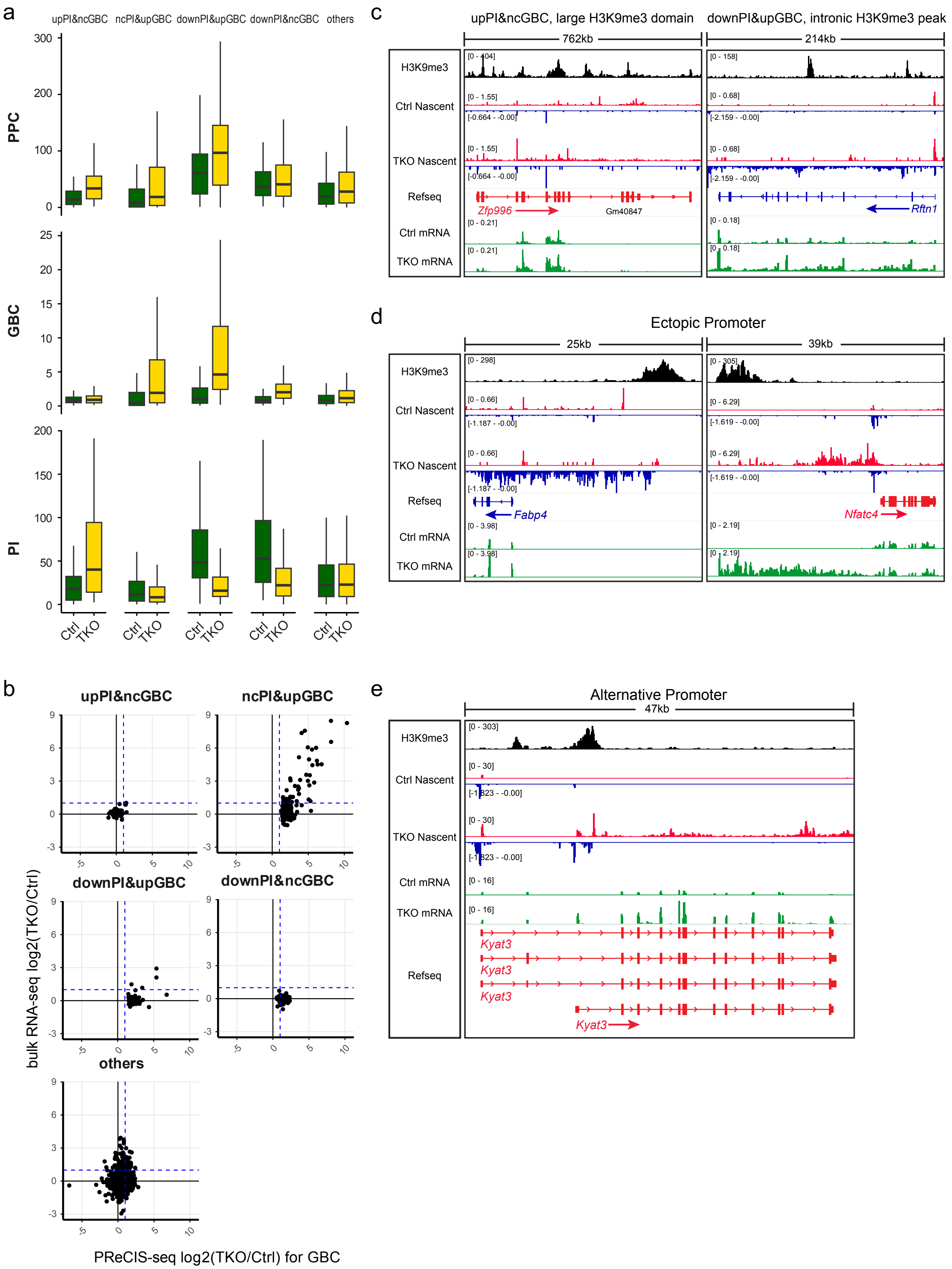

a
upPI&ncGBC ncPI&upGBC downPI&upGBC downPI&ncGBC others
PPC
GBC
PI
Ctrl TKO
b
upPI&ncGBC
ncPI&upGBC
downPI&upGBC
downPI&ncGBC
others
bulk RNA-seq log2(TKO/Ctrl)
PReCIS-seq log2(TKO/Ctrl) for GBC
c
upPI&ncGBC, large H3K9me3 domain
downPI&upGBC, intronic H3K9me3 peak
762kb
214kb
H3K9me3
Ctrl Nascent
TKO Nascent
Refseq
Ctrl mRNA
TKO mRNA
Zfp996
Gm40847
Rftn1
d
Ectopic Promoter
25kb
39kb
Fabp4
Nfatc4
e
Alternative Promoter
47kb
Kyat3

Supplementary Fig. 12 | H3K9me3 loss results in increased nascent transcription at specific genes

a Box plot showing promoter proximal counts (PPC), gene body counts (GBC) and pausing index (PI) for different gene categories from main Fig. 6. Counts are normalized with spike-in. Box plots show the median (center line), interquartile range (box; 25th–75th percentiles), and whiskers extending to the minimum and maximum values no further than 1.5× the interquartile range. **b** Dot plot showing the mature mRNA (bulk RNA-seq) expression fold changes (log2-transformed) vs nascent RNA level changes for different gene categories. **c** IGV tracks showing examples of upPI&ncGBC and downPI&upGBC genes. See also main Fig. 6e for other examples. **d, e** IGV tracks showing examples of genes activated from ectopic promoter (*Fabp4*, *Nfatc4*) or alternative promoter (*Kyat3*).

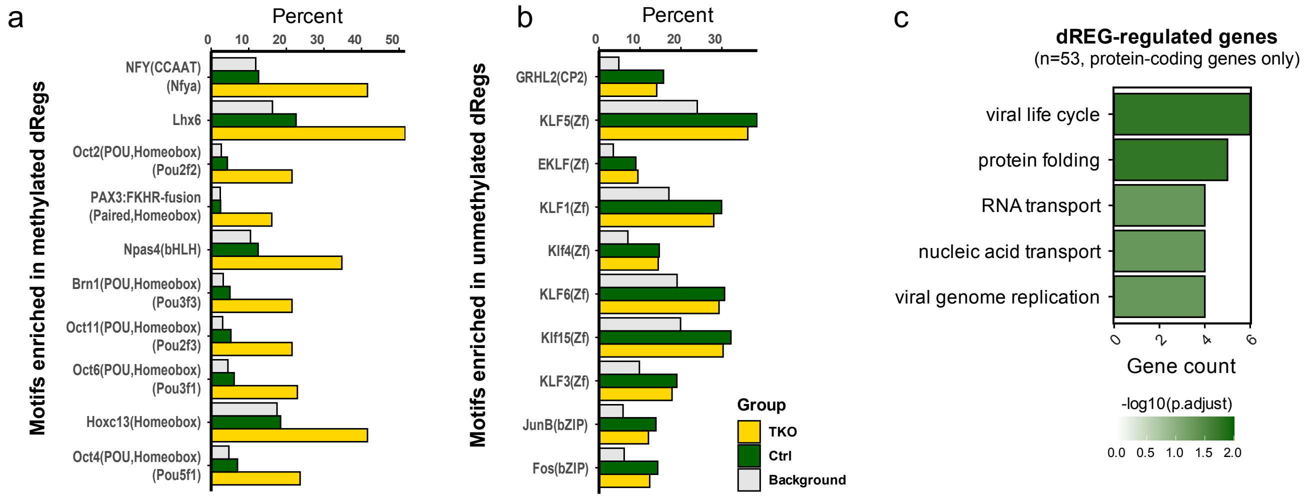

d

Genes within ±10kb vicinity of TKO-activated dREGs
(protein coding genes only)

Gene	Category	Regulated Processes
Mlf2	Transcription co-factor	Oncogen, leukemogenesis, metastasis
Hnrnpa2b1	RNA binding	Cancer progression, RNA stability
Ddx6	RNA helicase	P-body, neuronal differentiation, embryogenesis
Pin1	Protein isomerease	Bone cell differentation, germ cell development, signaling
Irx2	Transcription factor	Embryonic development, pattern formation
Med13	Mediator	
Atp5g3	ATP synthase	Energy metabolism
Pgrmc1	Receptor	Hormone signaling
Bhlhe40	Transcription factor	Circadian rhythm, immune cell differentiation
Col28a1	Collagen	Basement membrane compo-nent
Tlk1	Kinase	Chromatin assembly, DNA replication
Elmod1	GTPase activator	Ciliogenesis, protein traffic
Fgfbp1	Signaling	Angiogenesis, metastasis
Ppia	Signaling	Adipogenesis, osteogenesis
Eepd1	Endonuclease	DNA repair
Chmp1b	ESCRT-III complex	Endocytosis
Anxa1	Phospholipid binding	Anti-inflammatino, immune moedulation, hair follicle devel-opment
Nrarp	Notch Signaling	Neural crest development, angiogenesis

Genes are dysregulated from PReCIS-seq data. n=18 out of 53.

e

Genes regulated by TSS region H3K9me3
(protein coding genes only)

Gene	Category	Regulated Processes
Atf7ip	Setdb1 oo-factor	Hematopoiesis, osteogenesis, spermatogenesis, etc.
Tcfl5	Transcription factor	Spermatogenesis
Catsperg1	Ion channel	Sperm-specific expresion
Nnat[a]	Proteolipid	Neuronal differentiation, preadi-pocyte differentiation, etc.
Bglap3[a]	Osteocalcin family	Osteogenesis
Csta2	Cystatin A family	Cornified envelope formation, epidermis development
Trmt10c[b]	Methyltransferase	RNA modification
Senp7[b]	DeSUMOylase	Neuronal differentiation, immune regulation
Zfp951[b]	Transcription factor	
Impg2[b]	Extracellular matrix	Reginogenesis
Tasor[b]	HUSH complex component	H3K9me3 regulation, pluripoten-cy regulation
Pcdhga10[a] *Pcdhga9*[a] *Pcdhgb1*[a] *Pcdhga4*[a] *Pcdhgb5*[a] *Pcdhga8*[a] *Pcdhga7*[b] *Pcdhgb7*[b] *Pcdhga3*[b]	Protocadherin family	Neuronal development
Sh3g13 *Kbtbd12*[a]	–	Not reported

a: Genes only upregulated from bulk RNA-seq data
b: Genes only upregulated from PReCIS-seq data

Supplementary Fig. 13 | TKO-activated enhancers associated with specific TF motifs and genes important for cellular functions.

a, b Bar plots showing the percentage of motif-containing dREGs (TKO-activated) for factors in Fig. 7. Percentages were obtained from tables generated by HOMER when using default background. **c** Selected GO terms enriched in TKO-dysregulated genes (in Fig. 6b) nearby TKO-activated dREGs in Fig. 7b. GO analysis was performed using clusterProfiler based on a one-sided hypergeometric test with Benjamini–Hochberg correction for multiple comparisons. **d** Literature-supported function of genes regulated by TKO-activated dREGs. **e** Literature-supported function of genes regulated by TSS region (±500bp) H3K9me3.

References

1. Lyu, Y. *et al.* Stem cell activity-coupled suppression of endogenous retrovirus governs adult tissue regeneration. *Cell* **187**, 7414-7432.e26 (2024).
2. Dai, X. & Segre, J. A. Transcriptional control of epidermal specification and differentiation. *Curr. Opin. Genet. Dev.* **14**, 485–491 (2004).
3. Liu, S., Zhang, H. & Duan, E. Epidermal Development in Mammals: Key Regulators, Signals from Beneath, and Stem Cells. *Int. J. Mol. Sci.* **14**, 10869–10895 (2013).
4. Chovatiya, G. *et al.* Cell-type-specific RNA polymerase II activity maps in intact tissues provide a gateway to mammalian gene regulatory mechanisms in vivo. *Dev. Cell* **61**, 434-451.e8 (2026).
