# Supplementary material for: H3K9me3 controls epidermis morphogenesis by regulating RNA Pol II dynamics at developmental promoters and enhancers
Source: Nat Commun. 2026 May 15;17:6485. doi: 10.1038/s41467-026-73308-5 (PMC13377180; doi:10.1038/s41467-026-73308-5)
Supplement: Supplementary file 2 — Description of Additional Supplementary Files [file 41467_2026_73308_MOESM2_ESM.pdf]

## Description of Additional Supplementary Files

**File Name: Supplementary Data 1**
**Description:** Information of all embryo samples used in this study.

**File Name: Supplementary Data 2**
**Description:** Full tables of enriched GO terms for all GO analyses in this study. Each sheet contains one table and is named according to its related figure panels.

**File Name: Supplementary Data 3**
**Description:** Differentially expressed genes for all scRNA-seq clusters.

**File Name: Supplementary Data 4**
**Description:** Differentially expressed genes from E16.5 bulk RNA-seq data.

**File Name: Supplementary Data 5**
**Description:** Cluster assignments for H3K9me3 landscape nearby genes.

**File Name: Supplementary Data 6**
**Description:** Differentially expressed genes, differentially paused genes and pausing category assignment with PReCIS-seq data.
