# Supplementary material for: H3K9me3 controls epidermis morphogenesis by regulating RNA Pol II dynamics at developmental promoters and enhancers
Source: Nat Commun. 2026 May 15;17:6485. doi: 10.1038/s41467-026-73308-5 (PMC13377180; doi:10.1038/s41467-026-73308-5)
Supplement: Supplementary file 9 — Reporting Summary [file 41467_2026_73308_MOESM9_ESM.pdf]

nature portfolio

Corresponding author(s): Tudorita Tumbar

Last updated by author(s): Apr 1, 2026

Reporting Summary

Nature Portfolio wishes to improve the reproducibility of the work that we publish. This form provides structure for consistency and transparency in reporting. For further information on Nature Portfolio policies, see our Editorial Policies and the Editorial Policy Checklist.

Statistics

For all statistical analyses, confirm that the following items are present in the figure legend, table legend, main text, or Methods section.

n/a	Confirmed	
☐	☒	The exact sample size (*n*) for each experimental group/condition, given as a discrete number and unit of measurement
☐	☒	A statement on whether measurements were taken from distinct samples or whether the same sample was measured repeatedly
☐	☒	The statistical test(s) used AND whether they are one- or two-sided<br>*Only common tests should be described solely by name; describe more complex techniques in the Methods section.*
☒	☐	A description of all covariates tested
☐	☒	A description of any assumptions or corrections, such as tests of normality and adjustment for multiple comparisons
☐	☒	A full description of the statistical parameters including central tendency (e.g. means) or other basic estimates (e.g. regression coefficient) AND variation (e.g. standard deviation) or associated estimates of uncertainty (e.g. confidence intervals)
☐	☒	For null hypothesis testing, the test statistic (e.g. *F*, *t*, *r*) with confidence intervals, effect sizes, degrees of freedom and *P* value noted<br>*Give P values as exact values whenever suitable.*
☒	☐	For Bayesian analysis, information on the choice of priors and Markov chain Monte Carlo settings
☒	☐	For hierarchical and complex designs, identification of the appropriate level for tests and full reporting of outcomes
☒	☐	Estimates of effect sizes (e.g. Cohen's *d*, Pearson's *r*), indicating how they were calculated

Our web collection on statistics for biologists contains articles on many of the points above.

Software and code

Policy information about availability of computer code

Data collection	Data collection methods are thoroughly described in the Method section. The following softwares were involved in data collection.<br>FACS sorting: SONY LE-MA900 Cell Sorter Software v3.1.2<br>Fluorescence microscopy: LAS Application Suite X v3.9.1.28433 (Leica Microsystems); ZEN v3.0 (Carl Zeiss)
Data analysis	Data analysis pipelines are thoroughly described in the Method section. The following softwares or algorithms were in involved in data analyses.<br>FACS analysis: FlowJo, v10.10.0 (BD Biosciences)<br>Image analysis: Fiji v1.54p<br>Statistical analysis: Graphpad Prism v10.6.1<br>R and R packages: R v4.5.2; RStudio 2025.09.2; Seurat v5.4.0; DESeq2 v1.50.2; BRGenomics v1.12.0; ChIPseeker v1.46.1; clusterProfiler v4.18.2; simplifyEnrichment v2.4.1;<br>Linux softwares: dREG v20200515; bowtie2 v2.5.1; fastp v0.23.4; trimmomatic v0.39; UMI-tools v1.1.4; CellRanger v9.0.1; STAR v2.7.11b; Epic2 v0.0.52; deepTools v3.5.5; BEDtools v2.29.2; Homer v4.11<br>Genomic data visualization: Integrated Genomics Viewer (IGV v2.17.2)<br><br>Scripts for custom code used for data analyses and figure generation in this study are available at GitHub (https://github.com/ChrisBai-Epi/H3K9me3_EpiDevelopment_NatComm2026) and archived at Zenodo (DOI: 10.5281/zenodo.19358131).

For manuscripts utilizing custom algorithms or software that are central to the research but not yet described in published literature, software must be made available to editors and reviewers. We strongly encourage code deposition in a community repository (e.g. GitHub). See the Nature Portfolio guidelines for submitting code & software for further information.

Data

Policy information about availability of data

All manuscripts must include a data availability statement. This statement should provide the following information, where applicable:

- Accession codes, unique identifiers, or web links for publicly available datasets
- A description of any restrictions on data availability
- For clinical datasets or third party data, please ensure that the statement adheres to our policy

All genomic data including bulk RNA-seq, scRNA-seq, CUT&RUN and PReCIS-seq data have been deposited in NCBI's Gene Expression Omnibus (GEO) and are freely accessible through GEO Series accession number GSE300774 [https://www.ncbi.nlm.nih.gov/geo/query/acc.cgi?acc=GSE300774].
Single cell RNA-seq data for Setdb1 knockout is available from GEO via GSE233240 [https://www.ncbi.nlm.nih.gov/geo/query/acc.cgi?acc=GSE233240].
Source data are provided with this paper.

Research involving human participants, their data, or biological material

Policy information about studies with human participants or human data. See also policy information about sex, gender (identity/presentation), and sexual orientation and race, ethnicity and racism.

Reporting on sex and gender	*Use the terms sex (biological attribute) and gender (shaped by social and cultural circumstances) carefully in order to avoid confusing both terms. Indicate if findings apply to only one sex or gender; describe whether sex and gender were considered in study design; whether sex and/or gender was determined based on self-reporting or assigned and methods used.<br>Provide in the source data disaggregated sex and gender data, where this information has been collected, and if consent has been obtained for sharing of individual-level data; provide overall numbers in this Reporting Summary. Please state if this information has not been collected.<br>Report sex- and gender-based analyses where performed, justify reasons for lack of sex- and gender-based analysis.*
Reporting on race, ethnicity, or other socially relevant groupings	*Please specify the socially constructed or socially relevant categorization variable(s) used in your manuscript and explain why they were used. Please note that such variables should not be used as proxies for other socially constructed/relevant variables (for example, race or ethnicity should not be used as a proxy for socioeconomic status).<br>Provide clear definitions of the relevant terms used, how they were provided (by the participants/respondents, the researchers, or third parties), and the method(s) used to classify people into the different categories (e.g. self-report, census or administrative data, social media data, etc.)<br>Please provide details about how you controlled for confounding variables in your analyses.*
Population characteristics	*Describe the covariate-relevant population characteristics of the human research participants (e.g. age, genotypic information, past and current diagnosis and treatment categories). If you filled out the behavioural & social sciences study design questions and have nothing to add here, write "See above."*
Recruitment	*Describe how participants were recruited. Outline any potential self-selection bias or other biases that may be present and how these are likely to impact results.*
Ethics oversight	*Identify the organization(s) that approved the study protocol.*

Note that full information on the approval of the study protocol must also be provided in the manuscript.

Field-specific reporting

Please select the one below that is the best fit for your research. If you are not sure, read the appropriate sections before making your selection.

[x] Life sciences [ ] Behavioural & social sciences [ ] Ecological, evolutionary & environmental sciences

For a reference copy of the document with all sections, see nature.com/documents/nr-reporting-summary-flat.pdf

Life sciences study design

All studies must disclose on these points even when the disclosure is negative.

Sample size	The sample size was determined based on common practice of the field. We tried to minimize unnecessary animal use while maintaining sufficient statistical power.
Data exclusions	Data from unsuccessful knockout samples were excluded because they are irrelevant to the overarching goal of the study. The exclusion criteria was pre-established: samples were excluded if staining for H3K9me3 did not show any depletion.
Replication	All experimental data were reliably reproduced in multiple independent experiments as indicated in the figure legends.
Randomization	Comparisons were made between samples with different genotypes. No randomization was needed.
Blinding	The experimental groups were set up according to genotypes of mice (Ctrl vs TKO). Investigators were not blind to group allocation. But all experiments were performed to both groups at the same time with the same procedure. No additional covariates were introduced.

Reporting for specific materials, systems and methods

We require information from authors about some types of materials, experimental systems and methods used in many studies. Here, indicate whether each material, system or method listed is relevant to your study. If you are not sure if a list item applies to your research, read the appropriate section before selecting a response.

Materials & experimental systems

n/a	Involved in the study	
☐	☒	Antibodies
☒	☐	Eukaryotic cell lines
☒	☐	Palaeontology and archaeology
☐	☒	Animals and other organisms
☒	☐	Clinical data
☒	☐	Dual use research of concern
☒	☐	Plants

Methods

n/a	Involved in the study	
☒	☐	ChIP-seq
☐	☒	Flow cytometry
☒	☐	MRI-based neuroimaging

Antibodies

Antibodies used	Primary antibodies for IF staining: H3K9me3 (Abcam, cat. ab8898, 1:10,000, used for most experiments), H3K9me3 (Cell Signaling Technology, cat. 5327T, 1:1000, used when co-staining with other rabbit primary antibodies), Krt14 (Biolegend cat. 906004, 1:20,000), Krt10 (Abcam, cat. ab9026, 1:2000), Krt8 (Biolegend cat. 904804, 1:1000), Integrin-α6 (BD Pharmingen, cat. 555734, 1:2000), P-Cadherin (R&D Systems, cat. AF761, 1:1000), Involucrin (Invitrogen, cat. PA5-104453, 1:1000), Laminin(α1) (1:500, Sigma-Aldrich, cat. L9393), BrdU (Abcam, cat. ab6326, 1:1000), Cleaved Caspase-3 (R&D Systems, cat. AF835, 1:2000), Suv39h1 (Invitrogen, cat. 702443, 1:500), Setdb1 (Protein-tech, cat. 11231-1-AP, 1:1000), Ki67 (Invitrogen, cat. 14-5698-82, 1:5000), HP1α (Invitrogen, cat. MA5-32018, 1:1000), AE13 (ImmuQuest, cat. IQ292, 1:200), γH2Ax (Abcam, cat. ab2893, 1:1000), TNFα (Invitrogen, cat. PA5-19810, 1:200), Cox2 (Cell Signaling Technology, cat. 12282T, 1:500), GFP (Abcam, cat. ab13970, 1:1000)<br>Secondary antibodies for IF staining: Donkey Anti-Chicken IgY H&L (FITC) (1:1000, abcam, ab63507), Goat anti-rabbit IgG (Alexa Fluor 594) (1:1000, Jackson ImmunoResearch Labs, 111-585-003), Donkey anti-Rat IgG (H+L) (FITC) (1:1000, Jackson ImmunoResearch Labs, 712-095-153), Donkey anti-goat IgG(H+L) (Alexa Fluor 568) (1:1000, abcam, ab175477), Gaot anti-mouse IgG (H+L) (1:1000, Invitrogen, A-11020), Goat anti-Chicken IgY (H+L) (1:1000, Invitrogen A-11041), Goat anti-rabbit IgG (H+L) (Cy5) (1:1000, Jackson ImmunoResearch 111-175-144)<br>For FACS: Cd49f-BV421 (Biolegend cat. 313624), Epcam-APC (Biolegend cat. 118214)<br>For CUT&RUN: rabbit αH3K9me3, Abcam, Cat#ab8898; rabbit αIgG, Cell Signaling Technology, Cat#3900<br>For PReCIS-seq: αGFP (Abcam cat#ab290)
Validation	All antibodies were commercially sourced. We relied on the validation statements by manufacturers and published literature work citing those antibodies.

Animals and other research organisms

Policy information about studies involving animals; ARRIVE guidelines recommended for reporting animal research, and Sex and Gender in Research

Laboratory animals	We use mice (Mus musculus) that are maintained on C57BL/6 or mixed strain background. The following mouse lines are involved:<br>C57BL/6 wild type line.<br>K14-CreERT2 line is actively maintained in Tumbar laboratory and was originally generated by Pierre Chambon laboratory.<br>Suv39h1 f/f; Suv39h2-/-; Setdb1 f/f triple-targeted line was gifted by Kenneth Zaret laboratory.<br>K14-Cre line is actively maintained in the Tumbar laboratory and was originally generated by Elaine Fuchs laboratory.<br>K14-H2BGFP line was generated by Tudorita Tumbar and has been actively maintained in Tumbar laboratory.<br>Polr2b f/f-GFP line was created by Gopal Chovatiya from the Tumbar laboratory.<br>Mice are crossed as described in figures and main texts. Embryos from E10.5-E18.5 were collected.
Wild animals	The study did NOT involve wild animals
Reporting on sex	Animal sex is not considered a primary variable in this study. Findings in this study apply to both sex.<br>All staining experiments were conducted on mouse embryos without knowledge of sex<br>All sequencing experiments include both males and females as replicates
Field-collected samples	The study did not involve samples collected from the field
Ethics oversight	All the mouse work was performed in accordance with the Cornell University Institutional Animal Care and Use Committee (IACUC)

Note that full information on the approval of the study protocol must also be provided in the manuscript.

Plants

Seed stocks

Report on the source of all seed stocks or other plant material used. If applicable, state the seed stock centre and catalogue number. If plant specimens were collected from the field, describe the collection location, date and sampling procedures.

Novel plant genotypes

Describe the methods by which all novel plant genotypes were produced. This includes those generated by transgenic approaches, gene editing, chemical/radiation-based mutagenesis and hybridization. For transgenic lines, describe the transformation method, the number of independent lines analyzed and the generation upon which experiments were performed. For gene-edited lines, describe the editor used, the endogenous sequence targeted for editing, the targeting guide RNA sequence (if applicable) and how the editor was applied.

Authentication

Describe any authentication procedures for each seed stock used or novel genotype generated. Describe any experiments used to assess the effect of a mutation and, where applicable, how potential secondary effects (e.g. second site T-DNA insertions, mosiacism, off-target gene editing) were examined.

Flow Cytometry

Plots

Confirm that:

- [x] The axis labels state the marker and fluorochrome used (e.g. CD4-FITC).
- [x] The axis scales are clearly visible. Include numbers along axes only for bottom left plot of group (a 'group' is an analysis of identical markers).
- [x] All plots are contour plots with outliers or pseudocolor plots.
- [x] A numerical value for number of cells or percentage (with statistics) is provided.

Methodology

Sample preparation

Dorsal skin was dissected from E12.5, E14.5 and E16.5 embryos for preparing single cell suspension. For E12.5 embryos, epidermis is only a single layer of cells so larger tissue chunks were dissected from K14-H2BGFP+ embryos for downstream processing. Dorsal skin tissue was digested at 4°C overnight in collagenase solution (0.1% collagenase type I, 0.1% collagenase type II, 1.25mM CaCl2, 1.25mM MgCl2 in HBSS). The following day, tissue/cells were rinsed with 500mg/L EDTA once, followed by digestion with trypsin solution (0.25% in Mg2+/Ca2+ free HBSS) at 37°C for 10min. Digestion was stopped by adding 2 folds volume of 15% FBS. Tissue was then dissociated by pipetting, and the resulting single cell suspension was sequentially forced through 70µm and 40µm cell strainers. Cells were washed and eventually resuspended with FACS buffer (5% FBS in Mg2+/Ca2+ free HBSS).

Instrument

SONY MA900

Software

SONY MA900 application

Cell population abundance

Cells were sorted using the 'Normal' option which balanced purity and yield. Target cell population represents > 90% of total sorted cells, which was examined by running post-sort cells through FACS.

Gating strategy

All events were sequentially gated for cells (FSC/SSC), Singlets (FSC-h/FSC-A), live cells (Propidium Iodide) and epidermal cells (Epcam+/Cd49f+). Gating for GFP+ population was additionally applied to E12 epidermal cells sorting.
Boundaries between positive and negative populations were set according to single color staining and unstained negative controls.

- [x] Tick this box to confirm that a figure exemplifying the gating strategy is provided in the Supplementary Information.
