# Supplementary material for: H3K9me3 controls epidermis morphogenesis by regulating RNA Pol II dynamics at developmental promoters and enhancers
Source: Nat Commun. 2026 May 15;17:6485. doi: 10.1038/s41467-026-73308-5 (PMC13377180; doi:10.1038/s41467-026-73308-5)
Supplement: Supplementary file 10 — Transparent Peer Review File [file 41467_2026_73308_MOESM10_ESM.pdf]

nature portfolio

Peer Review File

H3K9me3 controls epidermis morphogenesis by regulating RNA Pol II dynamics at developmental promoters and enhancers

Corresponding Author: Professor Tudorita Tumbar

This file contains all reviewer reports in order by version, followed by all author rebuttals in order by version.

Version 0:

Reviewer comments:

Reviewer #1

(Remarks to the Author)
In the submitted manuscript, entitled “H3K9me3 controls epidermis morphogenesis by regulating RNA Pol II dynamics at key developmental promoters and enhancers”, Bai and colleagues investigate the role of the histone mark H3K9me3 in organogenesis, specifically in the mouse epidermis. The authors first establish the presence of H3K9me3 during epidermal development and identify approximately 45,000 peaks bound by H3K9me3 in basal keratinocytes at E15.5, a subset of which overlap with cis-regulatory regions. Using a triple knockout (TKO) of all three H3K9me3 histone methyltransferases (HMTs)—Suv39h1, Suv39h2, and Setdb1—in an epithelial-specific manner, the authors demonstrate defects in skin morphogenesis, including impaired epidermal stratification, hair follicle formation, and sensory Merkel cell specification. These findings are supported by extensive immunofluorescence analyses examining lineage diversification and barrier function.
They further show that these defects are not due to changes in chromatin organization, DNA damage, or apoptosis, but instead result from decreased progenitor proliferation and compromised maintenance of basement membrane architecture. The authors also demonstrate that H3K9me3 regulates epidermal lineage diversification by repressing non-lineage genes. Finally, they show that loss of H3K9me3 impairs RNA Pol II recruitment to promoters at select targets and leads to aberrant activation of enhancers in the developing epidermis.
While the role of H3K9me3 in constitutive heterochromatin and transposable element silencing is well established, this study highlights its critical function in late development by revealing its role in epidermal lineage diversification. Overall, the data presented provide compelling evidence for the importance of H3K9me3 in epidermal morphogenesis. However, there are still a few major points that need to be addressed.

Major Issues
1. Although authors clearly demonstrate that H3K9me3 is expressed throughout the epidermis development, the precise expression pattern should be shown for each day since during this time, there are many developmental progresses happening for skin morphogenesis. Along the same line, Western Blot for the levels of H3K9me3 would be a better quantification. The authors also fail to explain why there is a reduction in H3K9me3 levels from E12.5 on.
2. It is unclear why the authors pick E15.5 to perform H3K9me3 Cut&Run for the naïve H3K9me3 landscape. A better characterization of H3K9me3 signals over time during skin morphogenesis might help selecting more timepoints. Understanding how the H3K9me3 landscape changes during E12.5-E18.5 would be useful to understand the dynamics of this histone mark and its role in lineage diversification. Authors also find that some of the H3K9me3 peaks appeared as discrete peaks near or within gene bodies. From the data, it seems these peaks are weaker than broad domains. If this is true, a further analysis of these discrete peaks would be crucial. Are these peaks at or near genes important for skin development?
3. To understand the role of H3K9me3 in epidermis development, the authors use epithelial specific TKO that is Cre-based and inducible via tamoxifen. Their regimen is 3 doses of tamoxifen on days E10.5, E11.5 and E12.5. Why are these days picked? A further characterization of the mouse model is required to understand the dynamics of H3K9me3. Are the effects being observed a cumulative one? Is one of the developmental stages more important than the rest for H3K9me3? Upon activation of the Cre, how is each step of lineage diversification affected? Having earlier time points characterized would be vital to answer these questions.
• When authors characterize the TKO model in Fig2, they look at cell numbers and epidermal thickness. They need to

demonstrate that this is not due to sectioning. Flow analysis would be a good addition here. Especially, when it comes to Merkel cell numbers per section. Also, Fig2L is uninterpretable.
• In Extended Data Fig2e, upon TKO it seems that there is a higher H3K9me3 signal in the dermis. Are the H3K9me3 signal in epidermis affecting dermal signal? Why is there such a difference in the signal? This needs to be addressed.
4. To demonstrate that the H3K9me3 loss doesn't induce major genomic instability, the authors look at HP1a staining, which is lost. But they say since there are still Dapi dense regions, DNA compaction is retained. This needs to be further evaluated. Is there a true compaction? What are the percentage of it? Also, the nuclei of TKO epidermis look more squished, a close-up image is required. Further analysis of these gross effects needs to be studied.
5. Authors conclude that H3K9me3 loss mainly affects the progenitor proliferation. In Fig3e, in TKO, EdU signal in dermis is also lost. Why is that? This needs to be addressed. Are failures in lineage diversification in epidermis affecting the dermis development?
6. The authors claim that H3K9me3-depleted cells fail to maintain basement membrane signaling functions, but they don't show any data to support such a claim.
7. It is not clear why the authors picked E16.5 for their single cell studies. Along the same lines, they prefer to do bulk RNA-seq in E15.5. Why are they switching their time points for different experiments? There is no clear explanation for this.
8. The authors show that a substantial part of the upregulated genes upon H3K9me3 loss are directly repressed by this mark, while the others are not. Are these two groups of genes enriched for certain GO-terms? Or are they important for different stages of development?
9. The authors further analyze the role of H3K9me3 in epidermis morphogenesis by using PReCIS-seq and look at Pol II regulatory steps. They find two groups of genes that show direct promoter repression versus distal enhancer repression. What are the differences between these two groups of genes? Which group is required for the lineage diversification? Why does H3K9me3 specifically target this small set of genes directly at the promoter? Are these genes activated again later development given that the H3K9me3 signals decrease over time? Or could these genes be required upon inflammation or so?
10. The authors find that the methylated versus unmethylated enhancers that are activated upon H3K9me3 loss have different TF domains. Is there an importance to this? Do authors see differences in these TF levels?
Minor Issues
1. Overall, the image quality of immunofluorescence in Fig2 can be improved. Especially, a close-up for Fig2j would be beneficial. And Fig2l needs to be improved, and addition of arrows might be helpful to orient the reader.
2. Overall, how the GO-terms are shown can be improved (in Fig1h and Fig4h). From this depiction, it is impossible to know p-values associated with GO-terms.
3. When IGV tracks are shown, H3K9me3 is set to have signal ranging 0-200, but in some instances, this cuts the tip of the peaks. The authors should increase the range to show the full peaks (Fig5f-g, Fig6e).
4. In Fig6c, in the second graph, the box and the description needs to be aligned.
5. In line 359, it should be "direct repression", not "direction".

Reviewer #2

(Remarks to the Author)
In this manuscript, authors studied the role of H3K9me3 in epidermal development using a mouse model with triple depletion of the three major H3K9me3 histone methyltransferases, Suv39h1, Suv39h2, and Setdb1. Although the important role of H3K9me3 in the process is not surprising, a comprehensive analyses is currently lacking in the field. Authors showed that this epigenetic modification is pivotal for normal keratinocyte differentiation, hair follicle development and Merkel cell specification. Extra mechanistic insights came from the experiments using a novel method PReCIS-seq developed in the group to dissect the function of H3K9me3 in transcriptional initiation. The study is well performed and manuscript is clearly written. Some comments here mean to help authors to improve their manuscript and to draw solid conclusions.
Major comments:
1. In the first section (row 135), authors concluded, 'Together, these data show that global H3K9me3 levels increase during epidermal development'. How did authors draw this conclusion? In figure 1b and 1c, H3K9me3 increases between E10.5 and E12.5 when the simple epithelial of the ectoderm commits to epidermal lineage. However, between E12.5 and E18.5 during epidermal stratification, H3K9me3 goes down. Authors should be more precise describing the results. They also performed H3K9me3 Cut&Run at E15.5, to study genome-wide localization and gene regulation. They could consider to perform the Cut&Run also for earlier time points, like E12.5, to study the differential regulation.
2. In different parts of the manuscript, authors performed clustering analysis of H3K9me3, Fig.1f', Fig. 5b' and Fig. 6d, using 10kb upstream of the promoter, and 10kb downstream of the gene. This gives rise to many regions without H3K9me3 signals, and thus some part of the clustering is not meaningful. At the same time, authors also concluded that H3K9me3 can cover enhancers, an important part of the mechanism. It would be much better to use larger regions around the gene, e.g., 50-100kb, to capture regulation of gene expression with H3K9me3 signals. This would help them to interpret differentially regulated genes. Or do this, in addition to 10kb distance, to compare the data.
3. In bulk RNA-seq analysis, 193 upregulated genes were detected in H3K9m3 loss epidermis. In contrast, using PReCIS-seq to detect nascent RNA, 451 genes were detected as 'dysregulated due to H3K9me3 loss'. In figure 6, it seems that upregulated has a smaller number, but it is not fully clear. Authors should describe these in a consistent way, so that readers can easily interpret the data. In this part of the work, authors focused on the mechanism of transcription initiation, but did not mention the functions of genes that are differentially regulated. Are they the same as those detected in the regular bulk RNA-seq? What is the overlap? This would also be nice to discuss.
4. For the single-cell RNA-seq analysis, authors selected basal cells from the epidermis to perform the analysis? What is the rationale and consideration of doing this? Since single-cell RNA-seq can detect different cell states, it would be good to also detect the cell state of differentiated cells, unless the authors have a particular reason to do this.

Minor comments:
Authors mentioned that analysis pipelines are only available upon request. It would be much better to deposit these on GitHub or similar domains, to share them. This is important for transparency and reproducibility of the study.

Reviewer #3

(Remarks to the Author)
Bai et al. performed triple knockout of Su39h1, Suv39h2 and Setdb1 in vivo to investigate the role of H3K9me3 in the development of mouse epidermis after the onset of keratin 14 expression. They found that the triple knockout impairs epidermis morphogenesis by disrupting progenitor proliferation, the diversification of cell fates, and stratification of the interfollicular epidermis, without causing significant DNA damage and apoptosis. Using single cell RNAseq, they identified an aberrant basal and suprabasal populations in the triple-knockout epidermis. Those aberrant populations differentially express genes related to epidermal morphogenesis and non-keratinocyte lineages. Using PReCIS-seq, the authors attempted to determine the mechanisms by which H3K9me3 regulates the differentially expressed genes and concluded that H2K9me3 mainly represses PolII initiation at promoters and enhancers rather than regulating the pause-release at promoters.

Overall, the manuscript is well constructed and provides valuable insights into the role of H3K9me3 in epidermal morphogenesis. The only concern is that the PReCIS-seq method is not published in a peer-reviewed journal and therefore has not been evaluated by disinterested experts. If the PReCIS-seq paper is already peer-reviewed and accepted but not yet published, then it is fine to rely on this method. If not, the authors may consider supporting their results using more traditional methods, such as CUT&RUN with differently phophorylated Rpb1 antibodies to validate both transcription initiation sites and pause release sites.

Otherwise, I only have minor comments:
1. Show dots for all bar plots.
2. Figure 1c: The comparison was done between E10.5 and E18.5 only. Interesting to see other comparisons. Is the decrease after E12.5 significant?
3. Figure 1h: may be better to highlight complete terms instead of showing words
4. Extended Data Fig. 2: it may be better to discuss mosaic H3K9me3 loss before Merkle cells and hair follicles development
5. Figure 3: Report the number of samples for each panel
6. How many mice per group were used for scRNAseq?
7. Figure 4c: it’s not clear what are the genes plotted in the heatmap. Marker genes for normal groups?
8. Figure 4e: text says, “Numerous genes previously implicated … were mis-regulated in TKO aberrant clusters”, but clusters other than the two TKO aberrant clusters are highlighted in the figure. It is confusing as the readers are not sure what cell types the authors want to highlight.
9. Figure 4d, e, f, g: the violin plots are so tight that it’s hard to tell which violin belongs to which.
10. Figure 6c: since PI is related to the ratio of PPC and GBC, it will be helpful to write out changes of PPC in the figure too.

Version 1:

Reviewer comments:

Reviewer #1

(Remarks to the Author)
In the submitted manuscript, entitled “H3K9me3 controls epidermis morphogenesis by regulating RNA Pol II dynamics at key developmental promoters and enhancers”, Bai and colleagues all the major and minor issues that were raised. The authors successfully demonstrate the dynamic role of the histone mark, H3K9me3, during epidermis morphogenesis, which is essential for lineage diversification, and established H3K9me3 as a critical determinant of skin development. There are only two minor comments, but otherwise this study is ready to be published in Nature Communications.
Minor comments
1) In line 191, it says “Supplementary Fig. 4c, d” but it should be “Supplementary Fig. 3c, d”.
2) In Fig4f, the authors show the Go-term analysis of differentially expressed genes between AberrBasal vs Basal and AberrDiff vs Differentiating populations. However, this panel would benefit from reorganization. For example, the heatmap is not annotated, I assume they are Basal 1-3, Differentiating 1-3, and HF, and Merkel cells. But this is not written. So either proper labeling, or a simplified version would be a lot easier to digest for the readers.

Reviewer #2

(Remarks to the Author)
In this manuscript, authors performed new experiments and revised some less clear descriptions in the previous version to address my questions. The experiments were well done. Especially, the results of the quantification of H3K9me3 staining and the time-course Cut&Run of H3K9me3 convincingly showed the levels and the roles of H3K9me3 controlling the lineage commitment during epidermal development. Their additional analysis and more clear description on H3K9 signals, single-cell analysis and the PReCIS-seq also addressed my concerns.

I do not have additional questions.

Reviewer #3

(Remarks to the Author)
The authors have addressed my concerns.

Open Access This Peer Review File is licensed under a Creative Commons Attribution 4.0 International License, which permits use, sharing, adaptation, distribution and reproduction in any medium or format, as long as you give appropriate credit to the original author(s) and the source, provide a link to the Creative Commons license, and indicate if changes were made.
In cases where reviewers are anonymous, credit should be given to 'Anonymous Referee' and the source.
The images or other third party material in this Peer Review File are included in the article’s Creative Commons license, unless indicated otherwise in a credit line to the material. If material is not included in the article’s Creative Commons license and your intended use is not permitted by statutory regulation or exceeds the permitted use, you will need to obtain permission directly from the copyright holder.
To view a copy of this license, visit https://creativecommons.org/licenses/by/4.0/

We thank all reviewers for reviewing our paper and for their constructive feedback and comments. We have addressed all the reviewers' comments with further clarification and rationale, text edits, additional experiments and further data analysis. We believe these revisions significantly strengthened the quality of the manuscript and we appreciate the reviewer's insight on this subject. The new experiments include H3K9me3 landscape characterization by IF staining with addition developmental time points, new H3K9me3 CUT&RUN datasets at 3 developmental time points, additional staining and quantifications of existing and additional samples from different induction schemes to strengthen previously weaker points, as well as new data analysis to address additional question posed by reviewers. Details of the revision are described in the following point-by-point responses.

Reviewer #1 (Remarks to the Author):

In the submitted manuscript, entitled "H3K9me3 controls epidermis morphogenesis by regulating RNA Pol II dynamics at key developmental promoters and enhancers", Bai and colleagues investigate the role of the histone mark H3K9me3 in organogenesis, specifically in the mouse epidermis. The authors first establish the presence of H3K9me3 during epidermal development and identify approximately 45,000 peaks bound by H3K9me3 in basal keratinocytes at E15.5, a subset of which overlap with cis-regulatory regions. Using a triple knockout (TKO) of all three H3K9me3 histone methyltransferases (HMTs)—Suv39h1, Suv39h2, and Setdb1—in an epithelial-specific manner, the authors demonstrate defects in skin morphogenesis, including impaired epidermal stratification, hair follicle formation, and sensory Merkel cell specification. These findings are supported by extensive immunofluorescence analyses examining lineage diversification and barrier function.
They further show that these defects are not due to changes in chromatin organization, DNA damage, or apoptosis, but instead result from decreased progenitor proliferation and compromised maintenance of basement membrane architecture. The authors also demonstrate that H3K9me3 regulates epidermal lineage diversification by repressing non-lineage genes. Finally, they show that loss of H3K9me3 impairs RNA Pol II recruitment to promoters at select targets and leads to aberrant activation of enhancers in the developing epidermis.

While the role of H3K9me3 in constitutive heterochromatin and transposable element silencing is well established, this study highlights its critical function in late development by revealing its role in epidermal lineage diversification. Overall, the data presented provide compelling evidence for the importance of H3K9me3 in epidermal morphogenesis.

We thank reviewer #1 for their comprehensive summary and overall positive evaluation of our work.

However, there are still a few major points that need to be addressed.

Major Issues

1. Although authors clearly demonstrate that H3K9me3 is expressed throughout the epidermis development, the precise expression pattern should be shown for each day since during this time, there are many developmental progresses happening for skin morphogenesis. Along the same line, Western Blot for the levels of H3K9me3 would be a better quantification. The authors also fail to explain why there is a reduction in H3K9me3 levels from E12.5 on.

To address this comment, we added additional time points along development, E10.5-E18.5, and quantified H3K9me3 levels. It is technically extremely challenging, if not nearly impossible, to obtain enough pure epidermal cells in sufficient amounts for Western blots from early embryos, which are very small with a single-layered ectoderm. To improve our quantification methods the IF signal level in the epidermis was normalized against nearby dermal cells, to account for sample-to-sample and technical variations, and careful pairwise statistical analysis of the data was also performed. The results confirm that H3K9me3 is increased in the epidermis at E12.5 relative to E10.5 and E11.5 and statistical analysis demonstrate that the levels are maintained in the epidermis thereafter, without a significant decrease (Fig. 1b, c, Supplementary Fig. 1a).

It is also worth noting that our current genetic tools (K14-Cre/ER) do not efficiently target early developmental stages when K14 is absent or weakly expressed; the H3K9me3 loss occurs during lineage diversification after E12.5, when H3K9me3 levels are already high and stable.

2. It is unclear why the authors pick E15.5 to perform H3K9me3 Cut&Run for the naïve H3K9me3 landscape. A better characterization of H3K9me3 signals over time during skin morphogenesis might help selecting more timepoints. Understanding how the H3K9me3 landscape changes during E12.5-E18.5 would be useful to understand the dynamics of this histone mark and its role in lineage diversification.

This is a very good point that require further explanation and additional experiments. Our initial rationale was that morphological changes in the triple knockout (TKO) skin at E16.5 are caused by gene expression changes that are initiated at the chromatin level

at the prior developmental stage. With this in mind we performed CUT&RUN at E15.5 to define the H3K9me3 naïve landscape of epidermis (in Cre- Ctrl embryos) that would influence the mature mRNA expression changes observed at E16.5 in TKO. To respond to the reviewer's comment, we now add CUT&RUN H3K9me3 landscapes from wild type embryos at E12.5, E14.5 and E16.5 (Fig. 1d-h and Supplementary Fig. 1). The data defines the H3K9me3 landscapes in the epidermis at different developmental stages and identifies substantial changes from E14.5 to E16.5, which corresponds to basal-suprabasal cell diversification (Fig. 1b). GO analysis demonstrates that the dynamic H3K9me3 peaks are associated with organ morphogenesis, epidermis development, and cell fate commitment, among other processes. This data reveals the dynamic nature of H3K9me3 during skin development, which we believe is a substantial and highly relevant addition to our study. We thank the reviewer for their excellent suggestion.

Authors also find that some of the H3K9me3 peaks appeared as discrete peaks near or within gene bodies. From the data, it seems these peaks are weaker than broad domains. If this is true, a further analysis of these discrete peaks would be crucial. Are these peaks at or near genes important for skin development?

Following this suggestion, we defined narrow peaks as peaks smaller than 2000bp, which is the first quartile of all peak size distribution (Supplementary Fig. 9d). These peaks are indeed weaker than broad domains (Supplementary Fig. 9e). We analyzed genes harboring narrow peaks within 10kb vicinity and found that these genes are enriched for development-related GO terms such as epidermis development, cell-substrate adhesion, establishment of cell polarity, etc. On the other hand, genes harboring broad peaks were not enriched for any GO terms clearly associated with development. (Supplementary Fig. 9g, h)

3. To understand the role of H3K9me3 in epidermis development, the authors use epithelial specific TKO that is Cre-based and inducible via tamoxifen. Their regimen is 3 doses of tamoxifen on days E10.5, E11.5 and E12.5. Why are these days picked? A further characterization of the mouse model is required to understand the dynamics of H3K9me3. Are the effects being observed a cumulative one? Is one of the developmental stages more important than the rest for H3K9me3? Upon activation of the Cre, how is each step of lineage diversification affected? Having earlier time points characterized would be vital to answer these questions.

We indeed have begun this project seven years ago by trying various induction schemes at different time points and various collection timepoints, attempting to maximize the efficiency of our knockouts. Since we knew that the KO was inefficient in adult skin, we always employed 2-3 injection to increase the efficiency, which is

additive. We now provide a summary schematic of the different inductions tested (Fig. 2a). Specific information is provided in a new supplementary table with detailed information (including litter ID, induction scheme, KO efficiency, time of collection, etc.) for all the tested induction schemes (Supplementary Data 1) and in figure legends (Fig. 2, Fig. 3, and Supplementary Fig. 2-3).

In brief, most of our samples were unfortunately mosaic (not full) knockouts irrespective of the induction scheme, making collection of samples meaningful for analysis extremely challenging. Provided reasonably efficient depletion, the phenotypes observed in the different induction schemes were similar. We now quantify the epidermis thickness at two developmental time points (E16.5 and E18.5) from several different induction schemes to make this point (Fig. 2e, f). Regarding earlier time points, H3K9me3 depletion is often incomplete by E15.5 but more advanced by E16.5 (Supplementary Fig. 2e-j), which may indicate gradual loss that require several days to complete. These difficulties make it challenging to assess in more details the phenotypes of H3K9me3 “loss” at early time points after induction.

For all these reasons, we favored earlier induction with late collection time to ensure enough time for complete H3K9me3 depletion and for full manifestation of the phenotype.

• When authors characterize the TKO model in Fig2, they look at cell numbers and epidermal thickness. They need to demonstrate that this is not due to sectioning. Flow analysis would be a good addition here. Especially, when it comes to Merkel cell numbers per section.

For cell number or epidermal thickness related quantification (e.g., Fig. 2c-f), each data point, represents one embryo and is an average value from at least 5 images from three separate embryo sections. As clearly seen in the data from multiple mice, variation between different sections were much smaller than differences between different genotypes. Therefore, we are confident with the accuracy of this quantification, which is done in accordance with the state-of-the art of the skin field.

Due to difficulties in obtaining additional TKO embryos with good H3K9me3 loss efficiency (see our point above) in the limited time for revisions, we are unable to provide additional FACS data on Merkel cells. However, we respectfully point out the scRNA-seq data of such FACS isolated cells indeed demonstrate that Merkel cells are missing (Fig. 4a, b), which corroborate our IF staining experiments and quantification with Merkel cell markers (Fig. 2k, l). In addition, we provide staining for neurofilament (NF) together with Merkel cell staining to illustrate more convincingly the formation (and lack off in TKO) of the touch dome to which Merkel cells contribute (Fig. 2m).

Also, Fig2L is uninterpretable.

We have enhanced Fig2L (updated Fig. 2i) and provided additional visual aids to make data more interpretable. To corroborate this point, we included additional IF data showing hair follicle defects in sagittal sections (Fig. 2h).

• In Extended Data Fig2e, upon TKO it seems that there is a higher H3K9me3 signal in the dermis. Are the H3K9me3 signal in epidermis affecting dermal signal? Why is there such a difference in the signal? This needs to be addressed.

On careful inspection of the data, we have not observed a consistent increase of H3K9me3 in the dermis of TKO embryos. Instead, we sometimes observed variations in the epidermal and/or dermal signal from sample to sample and even from image to image, which reflect compound biological heterogeneity and technical variations. For example, certain populations in the dermis have extremely high H3K9me3 level compared to others (e.g., Fig. 1b, E18.5 sample). Therefore, to account for such variations, we either quantified the fraction of H3K9me3+ cells in the epidermis (e.g., Supplementary Fig. 2g) or normalized their levels to the underlining dermal signals (Fig. 1b, c). Accurately quantifying the dermal H3K9me3 would require additional internal controls to account for technical variations, and while interesting, we feel it is not justified given the already extensive nature of our characterization of this mouse model. To reduce confusion, we replaced the original Extended Data Fig. 2e image with one that shows dermal H3K9me3 TKO levels comparable to control, which represents most observed cases (Supplementary Fig. 2g).

4. To demonstrate that the H3K9me3 loss doesn't induce major genomic instability, the authors look at HP1a staining, which is lost. But they say since there are still Dapi dense regions, DNA compaction is retained. This needs to be further evaluated. Is there a true compaction? What are the percentage of it? Also, the nuclei of TKO epidermis look more squished, a close-up image is required. Further analysis of these gross effects needs to be studied.

We thank the reviewer for making this important point. We now provide quantification for the nuclei shape as required (Supplementary Fig. 3c, d). Regarding the role of H3K9me3 in heterochromatin maintenance, we point out that a cell culture study has already demonstrated that decompaction of the heterochromatin requires not only TKO for H3K9me3 but also knockout of three additional enzymes that are responsible for H3K9me1/me2[1]. In the study[1], the authors performed FISH with probes targeting major satellite repeat (MSR) for better demonstration. In our case, we are not confident that simple quantification of DAPI foci alone would accurately assess the level of DNA compaction. Additional in-depth characterization of this question makes the focus of an

entirely different study and is not possible here given our focus on organogenesis and already the extensive nature of our study. Therefore, we decided to remove the claim that heterochromatin is still compacted and only show HP1a dispersal in our study (Supplementary Fig. 3a, b).

5. Authors conclude that H3K9me3 loss mainly affects the progenitor proliferation. In Fig3e, in TKO, EdU signal in dermis is also lost. Why is that? This needs to be addressed. Are failures in lineage diversification in epidermis affecting the dermis development?

Given the extensive epithelial-to-mesenchymal crosstalk between epidermis and dermis, dermis development is likely indirectly affected, especially since many signaling genes are dysregulated in the epidermis, according to our scRNAseq of TKO epidermal cells. However, on inspection of EdU and Ki67 images we did not find sufficient support from our current data for the claim. In most cases the apparent decrease in EdU signal in the dermis that the reviewer noted is a secondary effect of the reduced number of hair follicle placodes or pegs, since hair morphogenesis is severely impaired in the TKO. To avoid distraction from the main points, we have replaced the images with those showing more comparable dermal EdU (Fig. 3e; Supplementary Fig. 3g)

6. The authors claim that H3K9me3-depleted cells fail to maintain basement membrane signaling functions, but they don't show any data to support such a claim.

We have provided data showing both defects in basement membrane organization by IF (Fig. 3k), as well as dysregulation of GO terms (e.g., cell-substrate adhesion, extracellular matrix organization, Supplementary Fig. 7d-g) and specific genes (e.g. Lamb1, Col28a1, Supplementary Fig. 7b) relevant for basement membrane organization and progenitor cell attachment. Epidermal progenitor attachment and basement membrane organization are well-recognized regulators of basal cell fate in the epidermis[2]. Disruption of these functions by gene knockouts of essential proteins, such as laminins and integrins, leads to severe defects in epidermis morphogenesis in mice[3]. In humans, genetic mutations in factors that lead to disruption of the basement membrane leads to severe blistering skin disease, known as epidermolysis bullosa[4]. We have before omitted this important information that is not general knowledge for the non-skin biologist, but now we make this rationale clear in the context of our data, with appropriate citations.

7. It is not clear why the authors picked E16.5 for their single cell studies. Along the same lines, they prefer to do bulk RNA-seq in E15.5. Why are they switching their time points for different experiments? There is no clear explanation for this.

We performed both scRNA-seq and bulk RNA-seq on E16.5 samples. However, the CUT&RUN data presented in the previous version were obtained from E15.5 samples. The rationale has been discussed in the response to comment 2, but briefly again we believed the mature mRNA we detect at E16.5 is more a reflection of the prior landscape of naïve chromatin at E15.5. In addition, as discussed above we now add additional CUT&RUN time points, including at E16.5 (Fig. 1 and Supplementary Fig. 1).

8. The authors show that a substantial part of the upregulated genes upon H3K9me3 loss are directly repressed by this mark, while the others are not. Are these two groups of genes enriched for certain GO-terms? Or are they important for different stages of development?

This is an important question that we indeed missed in our initial analysis. This question is closely related to the next comment (#9), and enhancer-mediated regulation is discussed in more detail in Fig. 7, so we decided to fully address them altogether. Briefly here, we now include additional analysis on the lists of genes directly repressed by H3K9me3 at promoters, vs genes regulated by TKO-activated dREGs (or enhancers). Unfortunately, when split this way, GO analysis does not show any significant GO terms, likely due to the small gene numbers, especially for promoter-regulated genes. Thus, we instead provide the list of genes regulated by either mechanism along with their known functions from literature (Supplementary Fig. 13c-e).

9. The authors further analyze the role of H3K9me3 in epidermis morphogenesis by using PReCIS-seq and look at Pol II regulatory steps. They find two groups of genes that show direct promoter repression versus distal enhancer repression. What are the differences between these two groups of genes? Which group is required for the lineage diversification? Why does H3K9me3 specifically target this small set of genes directly at the promoter? Are these genes activated again later development given that the H3K9me3 signals decrease over time? Or could these genes be required upon inflammation or so?

We have included further analysis of genes repressed by H3K9me3 at promoter vs enhancer. The results indicate that both groups contribute to lineage diversification (Supplementary Fig. 13c-e).

How H3K9me3 is targeted to promoters of specific genes remains unclear and require extensive future investigation; it may be related to binding of specific TFs that interact with HMTs or other recruitment mechanisms. Given the extent of our study and the time constrains for revisions, this in-depth mechanistic question is beyond the scope of the current manuscript. We now add a clear statement about this important future direction in the discussion.

10. The authors find that the methylated versus unmethylated enhancers that are activated upon H3K9me3 loss have different TF domains. Is there an importance to this? Do authors see differences in these TF levels?

We showed in Fig. 7j that TFs that bind to motifs enriched in the aberrantly activated methylated enhancers are normally highly expressed and do not change expression upon TKO. Thus, TFs that are normally purposed for epidermal lineage development are likely redirected to non-physiological enhancers, if H3K9me3 is not properly established.

As far as unmethylated enhancers, they also contain motif of TF that are normally highly expressed and do not change upon TKO. For these, there are likely indirect effects that lead to aberrant activation that are not trivial to pinpoint. We have already discussed these considerations in the previous version of the manuscript, but we now make it more clear in text.

Minor Issues

1. Overall, the image quality of immunofluorescence in Fig2 can be improved. Especially, a close-up for Fig2j would be beneficial. And Fig2l needs to be improved, and addition of arrows might be helpful to orient the reader.

We have enhanced original Fig2j (updated Fig. 2k), 2l (updated Fig. 2i) images with additional visual aids as suggested. Plus, we have stained for Merkel cells and neurofilament at E18.5 (Fig. 2m) and provided sagittal section images for hair follicle phenotype (Fig. 2h) to strengthen the data presentation.

2. Overall, how the GO-terms are shown can be improved (in Fig1h and Fig4h). From this depiction, it is impossible to know p-values associated with GO-terms.

We have replaced original Fig1h with a dot-plot-style GO representation where the size of dot reflects gene counts and color represents q values (Supplementary Fig. 10c). While we decided to keep the original Fig4h (updated Fig. 4f), we now provide bar graph in supplement data for specific GO terms (Supplementary Fig. 7d-g). All new GO analysis results are presented in either bar plots or dot plots, which shows adjusted p-values and gene counts. Additionally, the full GO analysis results were provided in Supplementary Data 2.

3. When IGV tracks are shown, H3K9me3 is set to have signal ranging 0-200, but in some instances, this cuts the tip of the peaks. The authors should increase the range to show the full peaks (Fig5f-g, Fig6e).

As suggested, we now use adaptive auto-scaling for most IGV browser tracks. But for tracks that meant to show broad domains, auto-scaling creates misleading impressions.

For these, we use both 0-200 scale as well as auto-scaling (Fig. 5f; Supplementary Fig. 9f) for complete depiction.

4. In Fig6c, in the second graph, the box and the description needs to be aligned.

We have better aligned the text with the box for a better presentation. We intentionally use different font size to indicate the magnitude of change, for example, with bigger font for ‘High recruitment’ and smaller font for ‘Medium recruitment’.

5. In line 359, it should be “direct repression”, not “direction”.
We have corrected the typo.

Reviewer #2 (Remarks to the Author):

In this manuscript, authors studied the role of H3K9me3 in epidermal development using a mouse model with triple depletion of the three major H3K9me3 histone methyltransferases, Suv39h1, Suv39h2, and Setdb1. Although the important role of H3K9me3 in the process is not surprising, a comprehensive analyses is currently lacking in the field. Authors showed that this epigenetic modification is pivotal for normal keratinocyte differentiation, hair follicle development and Merkel cell specification. Extra mechanistic insights came from the experiments using a novel method PReCIS-seq developed in the group to dissect the function of H3K9me3 in transcriptional initiation. The study is well performed and manuscript is clearly written. Some comments here mean to help authors to improve their manuscript and to draw solid conclusions.

We thank reviewer #2 for their positive evaluation and useful comments.

Major comments:

1. In the first section (row 135), authors concluded, 'Together, these data show that global H3K9me3 levels increase during epidermal development'. How did authors draw this conclusion? In figure 1b and 1c, H3K9me3 increases between E10.5 and E12.5 when the simple epithelial of the ectoderm commits to epidermal lineage. However, between E12.5 and E18.5 during epidermal stratification, H3K9me3 goes down. Authors should be more precise describing the results. They also performed H3K9me3 Cut&Run at E15.5, to study genome-wide localization and gene regulation. They could consider to perform the Cut&Run also for earlier time points, like E12.5, to study the differential regulation.

We thank the reviewer for this constructive feedback. The decreasing trend of H3K9me3 from E12.5, E16.5 to E18.5 is not statistically significant. We now have provided H3K9me3 staining data and related quantification on additional time points and have modified the text to be more precise in describing H3K9me3 changes across development. (Fig. 1b, c; Supplementary Fig. 1a)

In addition, following the reviewer's suggestion, we performed CUT&RUN profiling at 3 additional time points: E12.5, E14.5, and E16.5, showing dynamic regulation of H3K9me3. especially between the E14.5 and E16.5. The dynamic peaks were associated with genes important for development and lineage regulation. These data are now included as new Fig. 1d-h and Supplementary Fig. 1b-h.

2. In different parts of the manuscript, authors performed clustering analysis of H3K9me3, Fig.1f', Fig. 5b' and Fig. 6d, using 10kb upstream of the promoter, and 10kb downstream of the gene. This gives rise to many regions without H3K9me3 signals, and thus some part of the clustering is not meaningful. At the same time, authors also concluded that H3K9me3 can cover enhancers, an important part of the mechanism. It would be much better to use larger regions around the gene, e.g., 50-100kb, to capture regulation of gene expression with H3K9me3 signals. This would help them to interpret differentially regulated genes. Or do this, in addition to 10kb distance, to compare the data.

This is a very good suggestion. We have performed the same cluster analysis with 50kb gene-flanking regions and surprisingly find that H3K9me3 primarily occupy ~10kb upstream (for C2) or ~10kb downstream (for C3) from genes. The genes classified into each cluster thus overlaps significantly with the results obtained with 10kb flanking regions (Supplementary Fig. 10a-b). It is unclear why H3K9me3 is preferentially confined within this window. As expected, the most affected cluster is C4: many genes (n=1739) become C4 as more distal H3K9me3 peaks are included with 50kb flanks. On the other hand, however, some genes (n=1491) fall out from C4 because the expanding regions effectively 'shrink' the relevance of contribution from smaller narrow peaks, which makes them C5. Therefore, we decide to keep the original analysis with 10kb and provide the 50kb analysis in supplement.

3. In bulk RNA-seq analysis, 193 upregulated genes were detected in H3K9m3 loss epidermis. In contrast, using PReCIS-seq to detect nascent RNA, 451 genes were detected as 'dysregulated due to H3K9me3 loss'. In figure 6, it seems that upregulated has a smaller number, but it is not fully clear. Authors should describe these in a consistent way, so that readers can easily interpret the data. In this part of the work, authors focused on the mechanism of transcription initiation, but did not mention the functions of genes that are differentially regulated. Are they the same as those detected in the regular bulk RNA-seq? What is the overlap? This would also be nice to discuss.

We thank the reviewer for pointing this out. 451 represents genes from all four categories (upPI&ncGBC, ncPI&upGBC, downPI&upGBC, downPI&ncGBC), and they are all 'dysregulated' regarding engaged RNA Pol II activity. Some are dysregulated at promoter-proximal region alone and not at the gene body regions, as indicated by "ncGBC= no change gene body counts". On the other hand, 'upregulated genes' mostly have significant gene body transcriptional change. If analyzed using full transcript, only 232 genes are 'upregulated genes' at the nascent transcription level (Supplementary Fig. 11e). We have modified the text to better describe these results.

As suggested, we now added a comparison of ‘upregulated genes’ obtained with PReCIS-seq data vs RNA-seq data (Supplementary Fig. 11f-g). We do find a certain level of overlap, but many upregulated genes are not shared between the two methods. Theoretically, RNA-seq measures steady-state mature mRNA levels – a reflection of both preceding transcription levels and RNA stability. PReCIS-seq measures ongoing transcription or the distribution of transcriptionally engaged Pol II on the DNA. These differences, compounded with intrinsic technological biases, may contribute to poor overlap between the methods. PReCIS-seq is uniquely positioned for mechanistic insights that relate to RNA Pol II promoter-recruitment vs pause release, and hence we employed it here to expand on our analysis beyond steady-state mRNAs.

4. For the single-cell RNA-seq analysis, authors selected basal cells from the epidermis to perform the analysis? What is the rationale and consideration of doing this? Since single-cell RNA-seq can detect different cell states, it would be good to also detect the cell state of differentiated cells, unless the authors have a particular reason to do this.

This is a very good point, and it exposed a labeling error that we have now corrected. We intended to sort all Epcam+ cells, but according to the FACS result (Supplementary Fig. 5a), Epcam+ cells (marks epithelial cell) have mid-to high- α6 levels, meaning they are enriched in basal and immediate supra-basal cells, although we also capture some differentiating cell populations (Fig. 4a). It is possible that the suprabasal cells are more resistant to dissociation by the collagenase-Trypsin strategy and are under-represented here. We have now corrected the term ‘basal cells’ with ‘epidermal cells’ in the text.

Minor comments:
Authors mentioned that analysis pipelines are only available upon request. It would be much better to deposit these on GitHub or similar domains, to share them. This is important for transparency and reproducibility of the study.

We thank the reviewer for this feedback. We did not develop any original algorithms, and we performed all analyses using basic R packages and published packages (e.g., Seurat for scRNA-seq, DESeq2 for bulk RNA-seq, BRGenomics for PReCIS-seq, etc.). We have provided detailed description of our analysis pipeline in Method section, we anticipate readers would not have any problem reproducing the analysis in the study using the information we provided.

That said, we deposited all scripts containing the custom codes used in our analyses to Github and we have changed the ‘Code availability’ section accordingly.

Reviewer #3 (Remarks to the Author):

Bai et al. performed triple knockout of Su39h1, Suv39h2 and Setdb1 in vivo to investigate the role of H3K9me3 in the development of mouse epidermis after the onset of keratin 14 expression. They found that the triple knockout impairs epidermis morphogenesis by disrupting progenitor proliferation, the diversification of cell fates, and stratification of the interfollicular epidermis, without causing significant DNA damage and apoptosis. Using single cell RNAseq, they identified an aberrant basal and suprabasal populations in the triple-knockout epidermis. Those aberrant populations differentially express genes related to epidermal morphogenesis and non-keratinocyte lineages. Using PReCIS-seq, the authors attempted to determine the mechanisms by which H3K9me3 regulates the differentially expressed genes and concluded that H2K9me3 mainly represses PolII initiation at promoters and enhancers rather than regulating the pause-release at promoters.

Overall, the manuscript is well constructed and provides valuable insights into the role of H3K9me3 in epidermal morphogenesis. The only concern is that the PReCIS-seq method is not published in a peer-reviewed journal and therefore has not been evaluated by disinterested experts. If the PReCIS-seq paper is already peer-reviewed and accepted but not yet published, then it is fine to rely on this method. If not, the authors may consider supporting their results using more traditional methods, such as CUT&RUN with differently phophorylated Rpb1 antibodies to validate both transcription initiation sites and pause release sites.

We thank reviewer #3 for their encouraging comments. Our PReCIS-seq method and its validation is now published in *Developmental Cell* (Chovatiya et al., 2025)[5], which should be a strong warrant for the quality of this work.

Otherwise, I only have minor comments:

1. Show dots for all bar plots.

We have added dots for bar plots with fewer than 10 data points and replaced bar plots with larger sample size with box-and-whisker or violin plots.

2. Figure 1c: The comparison was done between E10.5 and E18.5 only. Interesting to see other comparisons. Is the decrease after E12.5 significant?

We have added new time points and provided test results for all pair-wise comparisons. The changes after E12.5 are not significant (Fig. 1b, c; Supplementary Fig. 1a).

3. Figure 1h: may be better to highlight complete terms instead of showing words

We now use classical bar plots or dot plots to present GO analysis results (e.g., Fig. 1g-h, Supplementary Fig. 10c), excepting Fig4h, for which we add bar plot representations in supplement data (Supplementary Fig. 7d-g).

4. Extended Data Fig. 2: it may be better to discuss mosaic H3K9me3 loss before Merkle cells and hair follicles development

We have made a major change for this part of figures with additional data and incorporated this suggestion.

5. Figure 3: Report the number of samples for each panel

Done.

6. How many mice per group were used for scRNAseq?

We have two embryos per group for scRNA-seq, which can be known from QC data (Supplementary Fig. 5c, d). The detailed information including the number of biological replicates for all genomic data is also reported in the Methods section.

7. Figure 4c: it's not clear what are the genes plotted in the heatmap. Marker genes for normal groups?

The genes plotted for Fig. 4c are genes aberrantly co-expressed in aberrDiff population that are normally separate markers of other normal keratinocyte sub-populations. More specifically, marker genes are first obtained for all "normal" cell clusters, while excluding aberrDiff. Then these marker genes are intersected with top 5000 expressed genes from aberrDiff.

To avoid misperception that aberrDiff only aberrantly co-expresses genes from other keratinocyte subpopulations, we also included top 500 aberrDiff specific genes not expressed by other keratinocyte subpopulations. All these genes together make a unique 'AberrDiff Gene Signature'. We have now explained this more clearly in the figure legend for Fig. 4c.

8. Figure 4e: text says, "Numerous genes previously implicated … were mis-regulated in TKO aberrant clusters", but clusters other than the two TKO aberrant clusters are highlighted in the figure. It is confusing as the readers are not sure what cell types the authors want to highlight.

We thank the reviewer for pointing this out. As seen in the figure, those genes highlighted with yellow box were dysregulated in all clusters from TKO sample (filled with yellow color in the graph), not only in the two aberrant populations. In other words, even the clusters that are not "aberrant" - because they largely keep their original

identity - display some changes in gene expression. We have modified that text to better deliver this message.

9. Figure 4d, e, f, g: the violin plots are so tight that it’s hard to tell which violin belongs to which.

We have adjusted the dimension of the violin plots and re-ordered the clusters to make it clearer. Due to the new space constraints, two of the four panels (original Fig. 4f, g) are moved to supplementary data (updated Supplementary Fig. 7b, c).

10. Figure 6c: since PI is related to the ratio of PPC and GBC, it will be helpful to write out changes of PPC in the figure too.

We tried plotting PPC vs GBC change in a way similar with Fig. 6b, but we noticed that some genes, while not significantly changed for either PPC or GBC, significantly increase PI in TKO, due to combined changes to the other two parameters. This is theoretically and mathematically valid. In other words, genes with upregulated PI could have either upregulated PPC or statistically unchanged PPC. Therefore, we do not think it is helpful to provide the single PPC change label. With that said, we do have box-and-whisker plots in supplement to show normalized values of PPC, GBC and PI for each gene category (Supplementary Fig. 12a). We hope this addressed the reviewer’s concern.

References

1. Montavon, T. *et al.* Complete loss of H3K9 methylation dissolves mouse heterochromatin organization. *Nat. Commun.* **12**, 4359 (2021).
2. Rousselle, P., Laigle, C. & Rousselet, G. The basement membrane in epidermal polarity, stemness, and regeneration. *Am. J. Physiol.-Cell Physiol.* **323**, C1807–C1822 (2022).
3. Raghavan, S., Bauer, C., Mundschau, G., Li, Q. & Fuchs, E. Conditional Ablation of β1 Integrin in Skin: Severe Defects in Epidermal Proliferation, Basement Membrane Formation, and Hair Follicle Invagination. *J. Cell Biol.* **150**, 1149–1160 (2000).
4. Georges-Labouesse, E. *et al.* Absence of integrin α6 leads to epidermolysis bullosa and neonatal death in mice. *Nat. Genet.* **13**, 370–373 (1996).
5. Chovatiya, G. *et al.* Cell-type-specific RNA polymerase II activity maps in intact tissues provide a gateway to mammalian gene regulatory mechanisms in vivo. *Dev. Cell* **61**, 434-451.e8 (2026).

2nd Response to reviewers' comments on the revised manuscript

There were two minor points from Reviewer 1, which we addressed below.

Reviewer #1 (Remarks to the Author):

In the submitted manuscript, entitled "H3K9me3 controls epidermis morphogenesis by regulating RNA Pol II dynamics at key developmental promoters and enhancers", Bai and colleagues all the major and minor issues that were raised. The authors successfully demonstrate the dynamic role of the histone mark, H3K9me3, during epidermis morphogenesis, which is essential for lineage diversification, and established H3K9me3 as a critical determinant of skin development. There are only two minor comments, but otherwise this study is ready to be published in Nature Communications.

Minor comments

1) In line 191, it says "Supplementary Fig. 4c, d" but it should be "Supplementary Fig. 3c, d".

We have corrected the mistake.

2) In Fig4f, the authors show the Go-term analysis of differentially expressed genes between AberrBasal vs Basal and AberrDiff vs Differentiating populations. However, this panel would benefit from reorganization. For example, the heatmap is not annotated, I assume they are Basal 1-3, Differentiating 1-3, and HF, and Merkel cells. But this is not written. So either proper labeling, or a simplified version would be a lot easier to digest for the readers.

We thank the reviewer for pointing this out. The heatmap represents a similarity matrix for the 359 GO terms enriched, so both rows and columns contain the same set of 359 GO terms, with similar terms clustered together. We have labeled this more clearly in the figure and provided more detailed description in the legend.

Reviewer #2 (Remarks to the Author):

In this manuscript, authors performed new experiments and revised some less clear descriptions in the previous version to address my questions. The experiments were well done. Especially, the results of the quantification of H3K9me3 staining and the time-course Cut&Run of H3K9me3 convincingly showed the levels and the roles of H3K9me3 controlling the lineage commitment during epidermal development. Their additional analysis and more clear description on H3K9 signals, single-cell analysis and the PReCIS-seq also addressed my concerns.

I do not have additional questions.

Thank you!

Reviewer #3 (Remarks to the Author):
The authors have addressed my concerns.
Thank you!
